# Method to assess the potential magnitude of terrestrial European avian population reductions from ingestion of lead ammunition

**DOI:** 10.1371/journal.pone.0273572

**Published:** 2022-08-29

**Authors:** Carolyn B. Meyer, Timothy A. Walker, Alex B. Francisco, Emily B. Morrison, Joseph S. Meyer

**Affiliations:** 1 Arcadis, Highlands Ranch, Colorado, United States of America; 2 Arcadis, Portland, Oregon, United States of America; 3 Arcadis, Concord, California, United States of America; 4 Arcadis, Cranberry, Maryland, United States of America; 5 Applied Limnology Professionals LLC, Golden, Colorado, United States of America; U.S. Geological Survey, UNITED STATES

## Abstract

Current estimates of terrestrial bird losses across Europe from ingestion of lead ammunition are based on uncertain or generic assumptions. A method is needed to develop defensible European-specific estimates compatible with available data that does not require long-term field studies. We propose a 2-step method using carcass data and population models. The method estimates percentage of deaths diagnosed as directly caused by lead poisoning as a lower bound and, as an upper bound, the percentage of possible deaths from sublethal lead poisoning that weakens birds, making them susceptible to death by other causes. We use these estimates to modify known population-level annual mortality. Our method also allows for potential reductions in reproduction from lead shot ingestion because reductions in survival and reproduction are entered into population models of species with life histories representative of the most groups of susceptible species. The models estimate the sustainability and potential population decreases from lead poisoning in Europe. Using the best available data, we demonstrate the method on two taxonomic groups of birds: gallinaceous birds and diurnal raptors. The direction of the population trends affects the estimate, and we incorporated such trends into the method. Our midpoint estimates of the reduction in population size of the European gallinaceous bird (< 2%) group and raptor group (2.9–7.7%) depend on the species life history, maximum growth rate, population trend, and if reproduction is assumed to be reduced. Our estimates can be refined as more information becomes available in countries with data gaps. We advocate use of this method to improve upon or supplement approaches currently being used. As we demonstrate, the method also can be applied to individual species of concern if enough data across countries are available.

## Introduction

Across the European Union, ECHA [1] estimates that hunting may disperse approximately 14,000 tonnes of lead gunshot and bullets (including slugs) in the terrestrial environment each year, leading to mortality of birds that ingest the lead ammunition. Researchers have studied impacts of such lead ingestion on birds in the field and laboratory [2–5], but much is still unknown about the magnitude of losses for terrestrial bird populations. Although Andreotti et al. [6] and Mateo [7] have estimated the magnitude of the waterfowl population deaths across Europe from lead ingestion, the effects in terrestrial bird populations is still highly uncertain because the method used for waterfowl cannot be easily applied to terrestrial birds. To obtain waterfowl estimates, both studies applied the estimates of added annual mortality from lead shot ingestion that Bellrose [8] provided in a North American waterfowl-tracking study to European waterfowl population size estimates. However, this method cannot currently be applied to terrestrial birds because important information currently is unavailable including: (1) long-term mortality tracking studies of terrestrial birds dosed and un-dosed with lead shot that would be equivalent to the Bellrose [8] waterfowl study, and (2) information required for the method on shooting bias and intestine residence time [9, 10]. Additionally, the method requires percentage of hunter-shot or captured birds with lead shot or bullet ingestion, which is available only for a few countries in Europe (Tables A and B in S1 Appendix). Pain et al. [9] used the Bellrose method in the UK to estimate losses of raptor and gallinaceous birds using professional judgment to fill in the data gaps (e.g., they assumed losses of over 200,000 pheasants and red-legged partridges in the UK).

In addition to lead shot, toxicity from lead bullet or slug fragments in prey or hunter-discarded offal consumed by raptors is of particular concern [11, 12]. Recently, researchers have evaluated effects of lead ammunition ingestion on eagle populations in the United States of America (USA) using tissue concentrations in carcasses combined with population modeling, but those methods either required relatively complete records of number of all bird deaths by lead poisoning in the study area (eastern USA [11]) or extensive banding and transmitter tracking data (western USA [13]). Because of this lack of information across countries in Europe, ECHA [1] was unable to develop a quantitative or qualitative estimate of the percentage of terrestrial European carnivorous birds with lead poisoning, and they developed only a highly uncertain estimate of 1% decrease in terrestrial game birds from lead shot ingestion. The data they cited to support the 1% estimate were mostly in the UK, and they mixed different statistics of mortality that are not comparable, such as percentage of necropsies (found dead birds) diagnosed as dying of lead shot ingestion and percent of a population of living birds dying of lead shot ingestion after accounting for background annual mortality that would occur anyway. Those two measures are often assumed to be the same but are not, and neither fully incorporates population dynamics (but see Green et al. [12], which recently developed a different method to assess losses in various raptor species using clinical liver thresholds and population modeling).

This paper describes an alternative 2-step approach to ECHA’s approach for estimating terrestrial bird population viability and reductions across Europe that does not require long-term studies of dosing effects, nor information on hunting bias or intestine residence time. Because ECHA’s objective was to estimate bird losses across Europe for all species combined, we demonstrate a method that can do the same but in a more robust manner and focusing only on two taxonomic groups. The first step of the method evaluates the percentage of carcasses that died from lead ammunition ingestion using existing European necropsy and pathology reports or available field tracking data with transmitters, and then converts that percentage into annual mortality rates. The second step uses population modeling that incorporates population dynamics to evaluate changes in population growth rate and size based on carcasses, as we did in Meyer et al. [14] and is being used in more recent studies of lead poisoning in raptors [11–13, 15]. Use of population modeling improves upon the commonly-used simplistic approach of assuming population sizes are decreased by the additional annual mortality. In addition to lethal poisoning, our method estimates potential mortality and reproductive effects from sublethal poisoning, usually not explicitly addressed [11–13, 15] or addressed only qualitatively in lead ingestion evaluations [1, 9]. Although our method still contains some uncertainty in estimated bird losses, the approach is more systematic and less qualitative than approaches that have been used for game birds in the European Union in ECHA [1] and for the United Kingdom (UK) in Pain et al. [9, 10]. Our proposed approach using models calibrated to actual population trends could result in more-informed management decisions for protecting and sustaining European avian populations.

Mortality from lead ammunition ingestion can be direct or indirect. Direct mortality is reported when lead ingestion is diagnosed as the cause of death, as discussed in Meyer et al. [14]. Indirect mortality is when reports attribute the death to another proximate cause but lead poisoning was the ultimate cause because it sublethally weakened the bird, increasing its susceptibility to death from the direct, proximate cause of death [16]. Some field studies suggest such sublethal effects are present [17–21], while others support that birds with sublethal lead levels are not predisposed to other causes of mortality [22]. Nonetheless, one or both types of mortality can be incorporated into the first step of our approach. If the bird has sublethal exposure and does not die, reproduction could also be impaired. This effect, although uncertain as to whether it exists in the field [22], can be quantitatively included in the first step and then combined with mortality in the second, modeling step.

Our 2-step approach is demonstrated on two taxonomic groups of species highly vulnerable to lead ammunition ingestion: (1) gallinaceous birds that obtain lead shot while foraging for seeds or grit and (2) diurnal carnivorous and scavenger raptors that feed in uplands and obtain lead shot or bullet fragments mostly from ingesting tissue of crippled hunted prey or carrion [4]. Only terrestrial-feeding diurnal raptors known to be susceptible to ingesting lead ammunition were included (hereafter, referred to as raptors), because nocturnal raptors often feed on rodents that do not have embedded shot and likely are less susceptible than diurnal raptors [7, 23–25]. Our demonstration focuses on exposure of diurnal raptors and gallinaceous birds to lead shot or bullet ingestion from upland soils of managed and unmanaged hunting areas across Europe but exclude shooting ranges.

As ECHA [1] did, we first estimated losses across species after aggregating the data into two groups composed of 6 game bird species and 13 carnivorous species known to be exposed to lead poisoning. Unlike the ECHA approach, our method also initially aggregates the data (1) by country, to weight effects by a country’s lead ammunition ingestion exposure relative to other countries, and (2) by population size of the species in each country. Our method also can be adapted to provide estimates for individual species of concern that have sufficient data, and we provide examples for four species: grey partridge (*Perdix perdix*), common buzzard (*Buteo buteo*), red kite (*Milvus milvus*), and bearded vulture (*Gypaetus barbatus*). When samples collected are relatively unbiased, aggregating the data provides an analysis representative of the avian species composition and abundance in Europe, weighted toward more abundant species. Splitting the data by species reduces sample sizes, increasing uncertainty in exposure, but evaluates differing exposures by species, which can be compared to the aggregated result. The main objective of this paper is to demonstrate a method to evaluate impacts of lead ammunition poisoning in Europe. This paper is not intended to definitively quantify reduction in the bird population from this stressor but rather demonstrate the method, and identify the additional data needed to improve on estimates using this method.

## Demonstration of the method

### Step 1: Estimating change in mortality and reproductive rates

The first step of the method assesses percentages of terrestrial bird carcasses collected in the field that died from lead ammunition ingestion or were sublethally exposed to the extent that susceptibility to death is enhanced or reproduction is impaired. Species of the two taxonomic groups selected were species resident or wintering in Europe that terrestrially feed (no wetland or waterfowl feeding). Species feeding mainly on insects or small mammals were excluded, as were migratory species that only breed in European countries (because these groups are less likely to be exposed to ammunition from European hunters.). Only countries or islands where a species is a year-round resident in Europe were included when tallying deaths and population sizes. Raptors were included if they had fledged from the nest. We conducted an extensive search of the peer-reviewed wildlife literature and necropsy- or liver-concentration studies or reports to obtain the information, as described below and in more detail in the S1 Appendix.

#### Change in mortality

**Fig 1:**
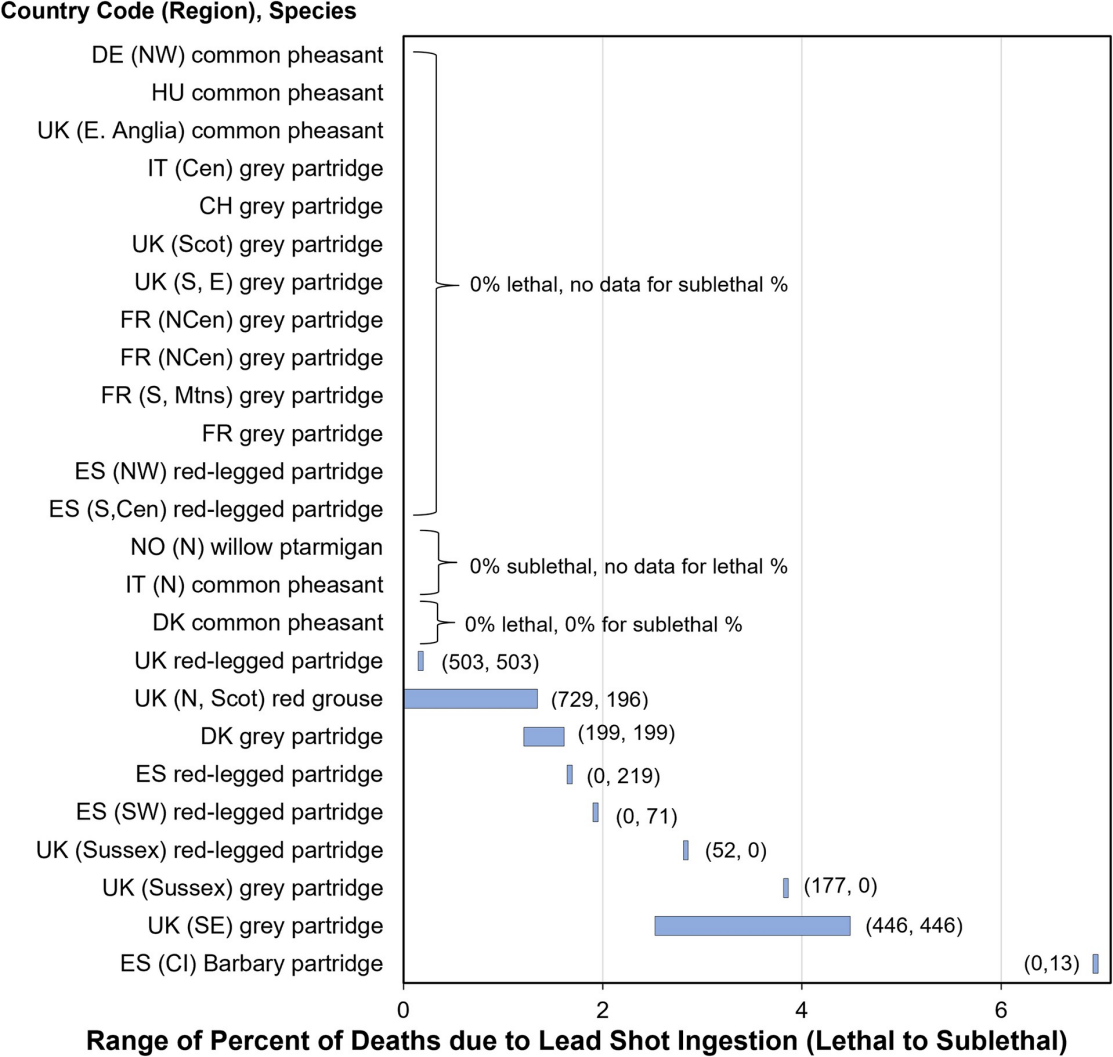
Estimated range of percentage of deaths due to lead shot ingestion in gallinaceous birds. Bars show range, where lower end is based on poisoning diagnosis as lethal and upper end includes all possible deaths ultimately from sublethal exposure based on ingesting one shot or subclinical liver concentrations. Pipe symbols (short bars) represent one number because sublethal or lethal estimates were unavailable. Scot = Scotland, Cen = central, CI = Canary Islands. Other abbreviations are European country codes and, in parenthesis, compass directions. Sample sizes of estimates (lethal, sublethal) are shown in parentheses.

**Fig 2:**
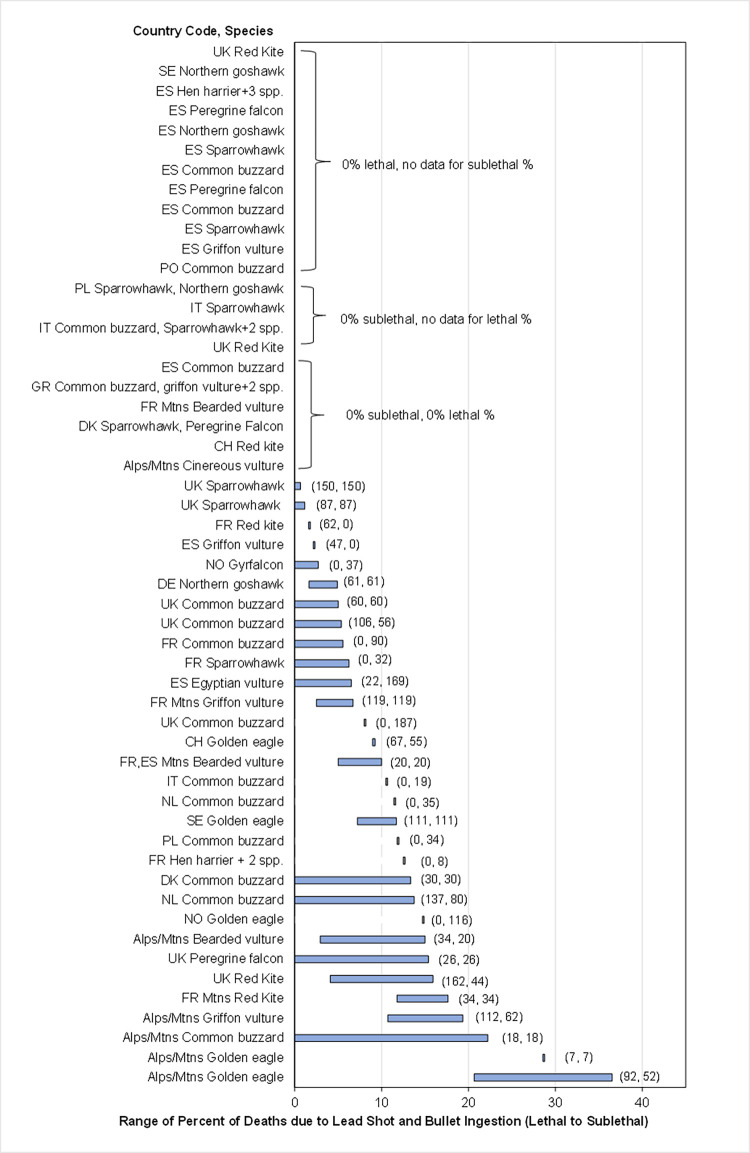
Estimated range of percentage of deaths due to lead ammunition ingestion in raptors (includes lead shot and bullets). Fig 1 describes bars, symbols, and abbreviations.

To estimate effects of lead ammunition ingestion on mortality, first the percentage of all found carcasses that died annually from lead ingestion was estimated from a compilation of data from many pathology or telemetry studies (S1 and S2 Tables). This focus on carcasses differs from the Bellrose [8] waterfowl method that estimated the proportion of living birds and did not estimate the proportion of dead birds that had ingested lead. The proportion of deaths comes from data on both direct and indirect causes of death by lead ingestion (Figs 1 and 2). The direct deaths are based on a pathologist’s diagnosis of lead poisoning and provide a lower-bound estimate because (1) some necropsy diagnoses may have missed lead poisoning as a cause and (2) they exclude deaths that had sublethal exposure and died directly of another cause. The indirect deaths are based on exceedance of subclinical liver lead concentrations and provide an upper estimate because they do not use a professional diagnosis and assume deaths even from another cause ultimately were from lead poisoning. The actual proportion of lead-caused deaths may be in the middle of the bounded range.

The studies that we included to estimate the proportion of direct deaths from lead poisoning were those that reported the cause of death for a relatively unbiased sample of carcasses (not restricted to those with suspected poisoning). These studies categorized the obvious proximate causes of deaths (e.g., powerline collision, predation, mowed over) and included a necropsy and sometimes a toxicological analysis for either all or a subset in which the cause of death was not clear. Carcasses too scavenged to identify cause of death were not included. Typically, if poisoning was suspected based on necropsy results, a toxicological analysis was performed, but these studies did not need to report or evaluate tissue lead concentrations or lead shot or bullet fragments in the gizzard or stomach to be included. The direct deaths rely solely on a researcher’s published causes of death or a veterinarian’s or pathologist’s reported diagnosis. Signs used in diagnoses of lead poisoning in gallinaceous birds include extreme emaciation combined with green staining and necrosis of the gizzard and gut with marked symptoms of anemia [26]; for raptors, signs can include gross and microscopic lesions, green staining around the vent, dilated gallbladder, and contracted talons [15, 27]. Sampling bias for “found” birds can affect the results as some carcasses with accidental deaths may be more easily found in human-accessible areas near roads or powerlines.

The proportion of deaths when indirect deaths are included (upper bound) is the proportion “ultimately” dying from lead poisoning. We based “ultimate” deaths (includes both lethal and sublethal deaths) generally on subclinical (2 to < 6 ppm wet weight, see Table A in S1 Appendix) or greater lead concentrations in liver tissues (see S1 Appendix for exceptions) or, when such data were lacking for gallinaceous birds, on proportion of gizzards containing at least one lead shot. Bone was not used because it reflects chronic exposure that may have occurred in the past and is not as correlated to death as liver concentrations [2, 28, 29]. One lead shot in the gizzard was chosen as a sublethal threshold for terrestrial game birds because pathologists have reported the direct cause of death was lead shot ingestion for 38% of grey partridges found with one to three lead shot in the gizzard in the UK [26]. Not until 4 or more lead shot were in the gizzard were 100% diagnosed as dying of lead shot poisoning. Possibly, some of these partridges that died of lead poisoning with one observed lead shot in the gizzard had consumed more lead shot that was not detectable by the time of the necropsy. Others may have actually only consumed 1 lead shot with no ultimate lethal effect and died of another cause as the pathologist indicated (zero effect). The one lead shot threshold was assumed to represent the sublethal threshold because the sublethal effect likely is between a zero effect and a direct lethal effect. In support, hunter-shot chukars (*Alectoris chukar*) in the US [30] and red-legged partridges (*Alectoris rufa*) in Spain [31] had percentages of livers with concentrations exceeding the subclinical threshold of 6 mg/kg dry weight (in Table A of S1 Appendix) that were more than 50% lower than their gizzard percentages containing lead shot (4.0% and 1.6% vs. 8.7% and 4.1%, respectively). Additionally, as expected, the one lead shot threshold always produced higher percentages of possible lead poisoning than the direct diagnosed percentages when both were measured in a study (S1 Table). Therefore, we used this definition; more liver tissue data in the future would reduce the uncertainty of using this gizzard-based threshold.

For the deaths assumed to be ultimately caused by lead ammunition ingestion, we did not include studies that only evaluated one or two found carcasses suspected of poisoning (not representative of all deaths), which could bias the proportion of all deaths due to lead ingestion high (such studies were included in [11]). Nevertheless, many of the studies included still have a sampling bias due to collection methods as discussed above. Some may have focused on reporting results only for found birds and carcasses with less obvious causes of death. Our upper bound is solely based on metrics of tissue concentrations or observed lead shot in the gizzard (not diagnoses), and likely overestimates loss because it uses a very low threshold for mortality occurring that is actually considered subclinical, and lead concentrations at clinical or even severely clinical concentrations are not definitive indicators of death [15, 32], particularly for gallinaceous birds [2]; a necropsy diagnosis may be more informative [33]. We believe this novel approach of bounding the uncertain range in this manner may be superior than assuming exceedance of a clinical or severe clinical tissue threshold results in death as done in other studies [11, 12, 15] or assuming only direct deaths are caused by lead poisoning [13].

To be able to evaluate subclinical reproductive effects, our models assume that not all subclinical exposure results in death [34–36], and that the midpoint of the bounded range (range shown in Figs 1 and 2) best represents the proportion that ultimately die from lead ammunition ingestion, which includes birds dying from either direct or indirect effects. Additionally, if a study identified that ammunition was not the source of elevated lead in some carcasses, those carcasses were not included in the estimates.

Across the reviewed studies, the percentages of directly-caused deaths and possible deaths ultimately caused by lead shot ingestion ranged from 0 to 6.9% for gallinaceous birds (Fig 1). For raptors, the percentage of deaths from lead ammunition ingestion (bullets and lead shot) was 0 to 37% for raptors (Fig 2). The higher percentages generally occurred in the mountainous areas, where big game hunting with rifles and bullets is common. The lower and zero percentages are generally in areas with small-game hunting that utilize lead shot.

These data represent dead birds collected over the years, with most collected before lead shot was banned in any terrestrial areas (some countries have upland bans, as discussed in [12, 14]). Although the percentages may vary depending on timing of when carcasses were collected, the direct and ultimate estimates typically were based on multiple years of data and are assumed for our purposes to represent overall annual percentages. Also, if hunting deaths occurred but were omitted from a study that relied on found carcasses (birds taken home in the bag would not be “found”), we adjusted the percentage of direct deaths from lead shot ingestion with an estimate of percentage of deaths from hunting to ensure the death percentage was based on birds that died of all causes, including hunting (i.e., percentages dying of each cause including hunting must sum to 100%). However, the estimates with indirect death percentages were not adjusted for hunting because hunter-shot birds could have been sublethally weakened and more easily shot, and therefore may have the same indirect death rates from lead as birds that died of other causes. In our analysis, all direct causes of death must sum to 100%. However, the possible ultimate percentage can have lead or other poisoning replace other proximal causes of death if they were the ultimate cause. Details on these methods and our assumptions and exceptions or other adjustments to fill data gaps for a country or species are provided in the S1 Appendix or S2 Appendix.

Exposure to lead ingestion differs by country and species [12]. To obtain an estimate for Europe that proportionately represents contributions of individual country, first we calculated the proportion of direct and ultimate deaths from lead ammunition ingestion by gallinaceous birds and raptors separately for each country or group of countries. Specifically, we summed the number of lead-impacted carcasses in S1 and S2 Tables in each study by country and then divided by total carcasses collected in the country (numbers of carcasses by country are shown in Tables 1 and 2). Although no weighting factor is explicitly applied, this method of pooling all carcass data across studies essentially weights studies with higher sample sizes more heavily (a study with a small number of carcasses evaluated is swamped out by studies with many when all are pooled together). Pooling may also implicitly account for species composition in the wild if it is assumed that carcasses collected that died from any cause in each study are representative of the species composition in the populations. This approach does not emphasize the most sensitive species of the two taxonomic groups that are exposed or restricted to highly local areas in Europe. Sensitive species in more local areas are best evaluated in those areas, rather than using our method of estimating losses across all countries in Europe.

**Table 1:** Gallinaceous bird carcasses (%) that died directly or ultimately of lead shot poisoning by country and across Europe.

Study Country	Carcass Sample Sizes of all Causes of Death and Breeding Pair Abundance used to Weight Lead Percentage for Europe				Direct %^d^	Possible Ultimate %^e^
	Carcasses for Direct %^a^	Carcasses for Ultimate %	Breeding Pairs in Study Country^b^	Breeding Pairs in all European Countries^c^		
United Kingdom	2318 (+413)	1145	2,655,000	2,940,770	0.99	2.06
France	503	0	1,490,049	1,490,109	0	*0*
Denmark	261 (+21)	261	111,000	211,322	0.36	0.38
Spain	231	303	5,003,140	5,756,165	0	1.93
Germany/Switzerland/Hungary	299	0	632,925	1,903,098	0	*0*
Italy	31	4	62,750	78,950	0	0
Norway	0	18	19,228	86,428	*0*	0
**Total Live Breeding Pairs in Europe**				**12,466,841**		
**Weighted mean % of deaths from lead shot ingestion in Europe**					**0.24**	**1.38**

^a^ Carcass sample sizes show number for lead poisoning-caused death, and in parenthesis is the number added as an estimate for those that died from hunting.

^b^ Population estimate of breeding pairs is from the most susceptible gallinaceous species in 2012 in study countries shown. Species included red-legged partridge, grey partridge, Barbary partridge (*Alectoris Barbara*), common pheasant, the UK red grouse (*Lagopus lagopus scotica*), and Norway’s willow grouse (*Lagopus lagopus lagopus*). This column represents study countries (not all European countries) and thus was not used to weight carcass percentages.

^c^ Population estimate is sum of breeding pairs in study country of the susceptible species, plus all other EU27 countries (including UK) plus Switzerland and Norway, that might have similar exposure to the study country, as defined in Table D of S1 Appendix. This column was used to weight death percentages before averaging because it includes all countries (note: weights for each country are country totals in this column divided by 12,466,811).

^d^ Italicized 0 means no direct data found, but the necropsies in the adjacent ultimate column support no lead poisoning from lead shot; thus, direct loss was assumed to be zero.

^e^ Italicized 0 means no tissue concentration data found, but direct necropsies numbering almost 100 to over 500 supported no lead poisoning from lead shot; thus, count of possible deaths ultimately caused by lead shot ingestion was assumed to be zero.

**Table 2:** Percent of diurnal raptor carcasses that died directly or ultimately of lead ammunition ingestion by country and across Europe.

Country	Carcass Sample Sizes of all Causes of Death and Breeding Pair Abundance Used to Weight Lead Percentage for Europe				Direct %	Possible Ultimate %
	No. Carcasses for Direct %	No. Carcasses for Ultimate %	Breeding Pairs in Study Country^a^	Breeding Pairs in all European Countries^b^		
United Kingdom	752	648	107540	121,689	0.80	5.25
Denmark/Netherlands	175	153	23,845	38,783	0	12.42
Spain/Portugal	2000	232	107,578	107,578	0.05	4.74
France	243	311	177,419	178,817	3.70	7.72
Italy/Austria/Switzerland	383	294	63,782	73,744	10.44	15.99
Germany/Poland	61	114	253,688	469,810	1.64	7.02
Greece	336	14	12,144	12,293	0	0
Norway/Sweden	178	264	96,319	114,401	4.49	11.74
**Total Live Breeding Pairs in Europe**				**1,117,112**		
**Weighted mean % of deaths by lead ammunition ingestion in Europe** ^**b**^					**2.52**	**7.90**

^a^ Population estimate in study countries shown of the most-susceptible species in 2012. Species included were red kite, common buzzard, Egyptian vulture (*Neophron percnopterus*, Canary Islands only), bearded vulture, griffon vulture (gyps fulvus), cinereus vulture (*Aegypius monachus*), peregrine falcon (*Falco peregrinus)*, golden eagle (*Aquila chrysaetos*), Bonelli’s eagle (*Aquila fasciata)*, northern goshawk (*Accipiter gentilis*), Eurasian sparrowhawk (*Accipiter nisus)*, hen harrier (*Circus cyaneus)*, and gyrfalcon (*Falco rusticolus)*. This column represents study countries (not all countries) and thus was not used to weight carcass percentages.

^b^ Population estimate is sum of breeding pairs in study country of the susceptible species, plus all other EU27 countries (including UK) plus Switzerland and Norway, that might have similar exposure to the study country, as defined in Table D of S1 Appendix. This column was used to weight death percentages before averaging because it includes all countries (note: weights for each country are country totals in this column divided by 1,117,112).

We then estimated the percentage for all of Europe (Tables 1 and 2), combining the above country estimates into an average estimate after weighting by breeding-pair population size in each country or group of countries reported for Europe (defined as the EU27 including UK but excluding Croatia). We used population-size data available at the time in the European Environment Information and Observation Network (EIONET) bird database [37]. When unavailable in that database, population sizes were from Musgrove et al. [38] for the UK gallinaceous birds, Herzog [39] for Swiss raptors, Heggøy and Øien [40] for Norwegian raptors, Centre for Agriculture and Bioscience International (CABI) database [41] for Swiss gallinaceous birds, and Norway’s Honsefugl Portalen database [42] for gallinaceous bird data. Game birds raised in captivity and released for shooting were not included in the population-size estimates unless the birds survived to be counted as a breeder that contributes to population viability. To be consistent, population sizes from all sources were estimates reported in 2012, the year in which our 50-year raptor modeling period begins. Weighting was applied to ensure lead-based death percentages in countries with the largest susceptible living populations have the strongest influence on the overall European estimate.

For countries without data on proportions of deaths from lead ammunition ingestion, we assigned carcass percentages from a country with studies that likely had similar lead exposure, based on information available about the various countries’ hunting practices and geography. Based on EIONET descriptions and other range maps [43], we included only population sizes of species resident to European countries, thereby excluding species that only breed in a European country and are exposed to lead ammunition during hunting season in non-European countries. We used breeding pairs instead of winter counts unless only winter counts were available, because tracking breeding-pair abundance of residents likely best represents the sustainability of European populations over time.

The final percentages after pooling across countries in Europe for gallinaceous birds were 0.2% for direct and 1.4% for the ultimate estimate of lead-shot-ingestion-caused deaths, with a midpoint estimate of 0.8% (Table 1). The corresponding weighted averages for raptors with bullets included were higher at 2.5% for direct and 7.9% for ultimate percentages of lead-ingestion-caused deaths, with a midpoint estimate of 5.2%.

We used the above midpoint estimates, assuming they represent the percentage of deaths of a typical European bird population exposed to lead ammunition ingestion. The midpoint estimate decreases the annual survival rate in a local population model in the second step of our approach. Specifically, we multiplied the annual survival of juvenile and adult age classes provided in published baseline (observed condition) stochastic population models of representative European species [14] by a factor (survival multiplier, SM) that incorporates the proportion of deaths due to lead ammunition ingestion. The multiplier is based on joint independent survival equations (S1 Appendix). Under the assumption of joint independent survival, some but not all birds that don’t die when lead is removed from their environment might then die of other, non-lead causes (e.g., some might then die of power-line electrocution, collision with an automobile, etc.) This approach accounts for all birds having a specified probability of death even when no lead ammunition is present to ingest in the environment. The following two joint probability equations give the survival multipliers used in the raptor and gallinaceous bird population models (discussed below in step 2), adapted from joint probability survival equations in Meyer et al. [14]:

$$Addition\;of\;Lead\;to\;Environment:\;SM=1-\frac{i∙M}{(1-i)+i∙M}\;$$ (1)

$$Removal\;of\;Lead\;from\;Environment:\;Gallinaceous\;Bird\;SM=\frac{1}{1-i∙M}$$ (2)

where *M* = observed total annual mortality proportion of the modeled baseline population exposed to lead, and *i* = proportion of deaths caused by lead ammunition ingestion. These equations are derived in S1 Appendix and differ because the presence of lead in the published local baseline model differed (absent in small-bodied raptor baseline models, present in gallinaceous bird and large-bodied raptor model; see modeling section). Based on our midpoint percentages estimated for all of Europe (0.8% and 5.2% discussed above), we set *i* to 0.008 for gallinaceous birds and to 0.052 for raptors.

#### Change in reproduction

Less is known about the effects of lead ammunition ingestion on reproduction of gallinaceous birds and raptors than mortality. Step 2 of our process estimates population-level effects with and without reducing reduction in reproduction, due to the uncertainty of estimating such reductions. To estimate the possible reduced reproduction from lead shot ingestion for gallinaceous birds, first the reproductive effects on individual birds that ingest lead shot was estimated. We researched the number of shot that might cause sublethal, but not lethal, effects, which was 3 lead shot (not less nor more as described in S1 Appendix). Based on a red-legged partridge laboratory study in Vallverdú-Coll et al. [44], ingestion of 3 lead shot was estimated to reduce hatching success in gallinaceous birds by 23%, except in pheasants (*Phasianus colchicus*). Pheasants appear to be more tolerant of lead shot ingestion [5] (Fig 1), and a laboratory study [45] supports that pheasant hatching success after ingesting 3 lead shot should be reduced by only 6% (see S1 Appendix for details on these estimates). Because the European population size dataset indicates 36% of the gallinaceous breeding pairs are pheasants, we used that proportion to weight hatching success reduction of pheasants when combined with non-pheasants. Our final weighted mean hatching success reduction for individual gallinaceous birds ingesting 3 lead shot was 17%.

Next, we determined the proportion of the population that would ingest 3 lead shot. Data in Butler et al. [46] indicated that 0.32% of live pheasants had exactly 3 pellets of lead shot in the gizzard when they were shot during hunts on Great Britain shooting estates. Because the 0.32% estimate occurs in the winter when ingestion of pellets is more prevalent, the percentage might be lower during the breeding season. However, some birds may have consumed lead pellets before sampling that were not detected in the gizzard, which would underestimate the proportion having consumed 3 shot. It is uncertain whether the two biases cancel each other out. We applied the 17% reduction to 0.32% of the gallinaceous bird population, reducing the fecundity term in the population model by 0.054.% (17% x 0.0032). For grey partridge populations, the hatching reduction is higher at 0.0736% (23% x 0.0032). Additionally, we reduced survival of gallinaceous chicks directly ingesting lead shot (part of fecundity) by the same relative rate as adults (same value of *i*), based on Potts’ [26] observation of grey partridge chicks ingesting lead shot. The fecundity multiplier (FM) that combines both types of reductions in the baseline model (accounting for joint chick mortality) is:

$$Gallinaceous\;Bird\;FM=\frac{1}{\left(1-H)(1-i∙M_{c}\right)}$$ (3)

where *H* = reduction in the population hatching rate from lead shot ingestion (0.00054 for all, 0.00076 for grey partridges only), *M*_*c*_ = observed chick mortality in the baseline population model, and *i* = proportion of chick deaths caused by lead shot ingestion (0.008 for all, 0.0011 for grey partridges only).

Developing a method to assess effects on raptor reproduction was more challenging because reproductive effects from lead ingestion are not always observed in the laboratory [47], and percentages of populations consuming lead shot and bullets in Europe at sublethal levels is unknown. We found one estimate in the literature of the possible reduction in reproduction of a raptor at sublethal levels, which was a 75% reduction in chicks fledged in the field (from an average of 2 to 0.5) from the Gil- Sánchez et al. [21] Bonelli eagle field study. That reduction percentage is uncertain, because (1) it was a correlative rather than controlled study, and (2) soil may have been a source of lead [36]. However, for demonstration purposes, we applied that reduction to the percentage of the living diurnal raptor population sublethally affected by lead shot and bullet ingestion that were assumed to survive to reproduce.

To estimate the proportion of the living population with reduced reproduction, we extrapolated from the carcass data. As stated previously, we assumed half of the collected dead raptors with sublethal levels of lead in their tissue did not ultimately die of lead ingestion (i.e., assumed when using a midpoint of the bounded range). Therefore, for the scenario with reduced reproduction, we assumed these raptors survived but had reduced reproduction before their death. This assumption might overestimate adverse effects for birds ingesting lead many months before reproducing but is useful for an upper bound of reproductive effects (no lower bound was evaluated). To calculate the percentage of the living birds that are sublethally affected, we assumed the proportion of all bird carcasses that ultimately did not die from lead ammunition ingestion but had sublethal lead concentrations would be similar to the proportion of all living birds that had sublethal but survivable concentrations. Using our European carcass percentages (Table 2), the sublethal percentage of the dead population of birds not dying from the lead when bullets and lead shot were included was calculated to be 2.85%. This percentage was assumed to represent the percentage of living birds that would have reproduction reduced by 75%, resulting in a reduction in fecundity (fledglings produced) by 2.14% (FM = 1–0.0214). This reduction was combined with reduction in juvenile survival to age 1 to obtain a fertility multiplier for the pre-breeding matrix population model (Table J in S1 Appendix).

### Step 2: Estimating change in population size, growth rate, and quasi-extinction rate

The second step of our approach incorporates population models. We entered the calculated survival and fecundity multipliers into four representative population models. We selected population models calibrated to actual population trends in Europe of species whose population dynamics are available and expected to be representative of the two taxonomic groups of interest. We used models of the grey partridge and three raptor species (common buzzard, red kite, and bearded vulture) to estimate change in population size and growth rate from the lead ammunition ingestion. For the raptors, the common buzzard and red kite are the surrogates to represent raptors with smaller body size. The bearded vulture represents the large-body raptors such as eagles and vultures that are long-lived, have delayed sexual maturity, and low reproductive rates and thus are likely more impacted by stressors to adult mortality than the smaller raptors [12, 48].

The common buzzard and red kite baseline raptor models in this demonstration are published models built from data on real, isolated local populations of common buzzards and red kites with very limited lead ammunition ingestion [14]. As such, we multiplied baseline survival rates in those raptor models by the survival multiplier developed from Eq 1, which reduces baseline survival of each juvenile and older age class, creating a scenario that includes lead ingestion. In contrast, the published baseline model for gallinaceous birds that we used is a grey partridge model of the European continental population exposed to lead shot ingestion [14, 49]. Therefore, for gallinaceous bird scenarios, we removed lead shot effects from the baseline population by applying Eq 2 as the survival multiplier in the grey partridge model. For the bearded vulture, we constructed the model from data recently published on the vulture’s population dynamics estimated from 1987 to 2016 in the Pyrenees in Spain, France, and Andorra [48] (details on the model parameters and structure are in S2 Appendix). Because of uncertainty in quantifying reproductive effects [1, 14], we ran a scenario for each model with and without reduced reproduction using the respective fecundity multipliers.

Except for the bearded vulture model, the three population models and their equations used in this analysis are described in detail in [14]. All four population models are available in the Dryad data depository. In brief, De Leo et al. [49] constructed the grey partridge model from life history information on the continental European population, and Meyer et al. [14] modified it to model lead shot ingestion effects. The model begins with the estimated spring population density each year, which is changed by a density-dependent spring growth rate composed of spring survival and fecundity followed by autumn and winter mortality rates, with rates randomly selected from lognormal distributions for each year. The environmental variation represented by the lognormal distributions is modeled stochastically with the standard deviations of spring growth rate and autumn-winter survival provided for the grey partridge [49]. The model was calibrated to grey partridge population trends from 1965 to 1993. Removal of lead shot was superimposed on the trajectory during that time period to evaluate the relative change in population size. The model represents the period when lead shot ingestion in partridges was high compared to earlier in the century [26]. This model was converted from the @RISK (v 7.50) format used in Meyer et al. [14] to be run in R [50] for the current study.

The common buzzard model was parameterized and calibrated on a population in Germany, and the red kite model on a population in Wales. Both areas have had minimal lead ammunition exposure since the 1950s. In contrast, the bearded vulture model was calibrated on a Pyrenees population with lead ammunition exposure. The models were initially developed using Poptools [51] and were converted and run in R for the current study. The models are one-sex stochastic matrix models with an annual time step that incorporates density dependence in fecundity and survival based on a theta-logistic equation fit to observed population growth data. A number of raptor studies support that both survival and fecundity can be density dependent [14, 48]. The bearded vulture model was constructed from Margalida et al. [48] data and is similar in structure to the other two raptors models (described in S2 Appendix).

After 10,000 runs, the output of each population model provides the mean change in the local population size of each age class, not the number dying. The model provides an estimate of the net number of breeding pairs “lost” from a local population from lead ammunition ingestion after accounting for both recruitment of new breeders into the population through reproduction (which included or excluded reductions in reproduction depending on the scenario) and removal of breeders through mortality. The models also predict the change in growth rate and quasi-extinction probability (reduction to low levels of concern), metrics important to evaluating the population sustainability.

Losses from the population in the published model can be scaled up to Europe by multiplying the modeled number lost for that one typical local population by the hypothetical number of local populations estimated to be in Europe, determined by the European total population size of the species that we evaluated and are known to ingest lead ammunition (Tables 1 and 2). For the partridge model, we used breeding birds/km^2^ for the initial population size, which were converted to breeding pairs by dividing by 2. However, when pheasants were reported as number of females, we used sex ratios of 1 male:4.6 females, following Pain et al. [10].

The magnitude of the effect of lead ammunition ingestion varies based on life history of a species, which we accounted for by selecting the four species with different rates of reproduction, longevity, and feeding habits. However, the effect also depends on whether the trend of the species modeled is increasing, decreasing, or stable in size [14]. Of the four calibrated population models, the grey partridge model represents a declining continental population at baseline, the common buzzard model represents a stable population at baseline (decreases with lead ammunition ingestion added), and the red kite and bearded vulture model represent increasing populations at baseline that eventually reach a steady state at equilibrium [14, 48]. Because raptors were evaluated with three different models representing different trends (stable for buzzard, both increasing and stable trends were evaluated for the kite and vulture), the lead ingestion results differed. To combine them for the final estimate for Europe, the results for small-bodied raptors were weighted in proportion to the number of small-bodied populations represented by each model’s trend direction (increasing versus those that were stable or decreasing) to achieve an estimate for small-bodied raptors in Europe. The same was done for large-bodied raptors. To obtain the final European-wide estimate of number of raptors lost to lead ammunition ingestion each year, the small- and large-bodied raptor results were finally weighted in proportion to the number of small and large-bodied raptors in Europe.

The overall direction of the trend of the different species of gallinaceous birds and raptors evaluated in Europe is available in the EIONET database for short- and long-term periods. The last two short-term datasets were used to estimate the percentage of small and large-body populations that are increasing, decreasing, or stable (defined as from 2001 to 2012 and from 2013 to 2018). Almost all (99%) of the EU27 gallinaceous bird populations have been declining in the short and long term (mainly due to intensive farming practices). Therefore, we assumed the model of a declining grey partridge population was appropriate to represent gallinaceous birds as a group. In contrast, few species of raptors were declining in Europe in the long term, with most of the susceptible populations of species by country listed as either increasing (90% of EU27 raptors) or stable (0.1%) since 1980. However, in the first 12-year short term period (2001–2012), this pattern reversed for the small raptors, with 93% decreasing, 3% stable, and 4% increasing. The pattern shifted again in the second short-term period (2013–2018) for small-bodied raptors, with more populations increasing (51% increasing, 49% decreasing, 0.04% stable). The large-bodied raptor trend direction did not change, because 99.5% of the large raptors were increasing in both short-term periods (only 0.5% were stable). Because population sizes used in this paper are based on the data countries provided during the end of the first short-term reporting period of 2012, the two short-term trends before and after that year are used independently to estimate the range of population reductions possible under the two different sets of trend directions.

To match populations with different trends to the population models, the small and large-bodied raptor species that were increasing are represented by the increasing trend results of the red kite and bearded vulture models, respectively. Small raptor species that were stable or decreasing are represented by the mean of the results of the common buzzard model and red kite model when at equilibrium. Large raptor species that were stable are represented by the bearded vulture model when it was at equilibrium. Because fewer individuals are available to be “lost” to lead ingestion in a declining population than a stable one at carrying capacity, use of the population models of a stable population at equilibrium at baseline for a declining species will, if anything, overestimate the magnitude of the numerical reduction in the European population size. The equilibrium models will model declines to a new equilibrium from lead ingestion and the associated change in probability of quasi-extinction. However, they may underestimate the probability of quasi-extinction (decreasing to a critical threshold level of abundance) for a steeply declining species that has not reached an equilibrium. Such individual dwindling populations of species could be addressed with our 2-step method if more data and population models calibrated to the declining trend of such species become available or are created, and we encourage use of our method for that purpose. This particular demonstration, however, is designed to address the objective of evaluating the general effects of typical, larger populations across Europe of a size similar to the population size on which the population models were built (3,000 ha estate for gallinaceous birds, 1750 breeding pairs for small raptors at carrying capacity, and almost 200 breeding territories for large raptors).

An advantage of aggregating species using a weighting method by country to assess lead-poisoned percentages is that it increases sample sizes of carcasses within each country and thus confidence in the estimates. Splitting exposure estimates by individual species produces more data gaps by country, requiring more uncertain assumptions on the similarity of exposure among groups of countries, or the use of a more uncertain surrogate for lead exposure such as hunter density (regressed on a lead-exposed percentage as was done in [12]). A disadvantage is that aggregating may misrepresent losses to key individual species that occur in large areas of Europe. We tested if this occurred by evaluating results for each of the four individual species on which the population models were built. These species, particularly the grey partridge and common buzzard, had enough data on exposure by country to be evaluated individually. The resident red kite and bearded vulture are restricted to fewer countries; but those often had lead ammunition exposure data, and thus were generally assessable.

Our approach assesses bird losses in Europe from lead ammunition ingestion rates that have predominated in the past, mostly before bans on lead shot in upland areas. The survival multiplier applied to the baseline autumn-winter survival in the grey partridge model to remove lead ammunition ingestion effects (including sublethal effects) was 1.0041 (equivalent to 0.41% relative reduction in survival caused by ingestion of lead). The fecundity multiplier was 1.0057 (equivalent to 0.57% reduction in reproduction caused by ingestion of lead). Because the modeled raptor population trends reach equilibrium, which can be projected into the future [14], the period of time modeled for the local small- and large-bodied raptor populations was from the year of most of the European raptor population estimates (i.e., 2012) to 50 years later, although the equilibrium analyses for red kite and bearded vulture also had to be analyzed for 50 years after they reached equilibrium to evaluate the effect of adding lead ingestion when these species are stable. The survival multiplier for lead ingestion applied in the raptor models ranged from 0.984 to 0.993 and from 0.989 to 0.992 for the age classes in the buzzard and red kite models that add lead ingestion (< 1.6% survival reduction), and from 1.00124 to 1.01545 in the bearded vulture model (< 0.16% survival reduction) that removes lead ingestion (Table J in S1 Appendix). The fertility (F_x)_ multipliers were 0.952, 0.966, and ≤ 1.039 for the buzzard, red kite, and bearded vulture matrix models, respectively.

After running the stochastic population models, we estimated and compared the probability of quasi-extinction (defined as population decline to a threshold number of concern after 30 years for the partridge and 50 years for raptors) for all modeling scenarios. We set quasi-extinction thresholds at 5 pairs per km^2^ and at just 350 breeding pairs (representing a population in 10,000 ha) for the partridge and small raptors models, respectively, following Meyer et al. [14]. Because the bearded vulture population was steadily increasing yet still below 350 breeding pairs or territories in the Margalida et al. [48] data in a 60,000 ha area, the quasi-extinction threshold for that model was set lower at 900 total individuals, a level that produced a non-zero probability of quasi-extinction when lead poisoning was included.

### Gallinaceous bird modeling results

**Fig 3:**
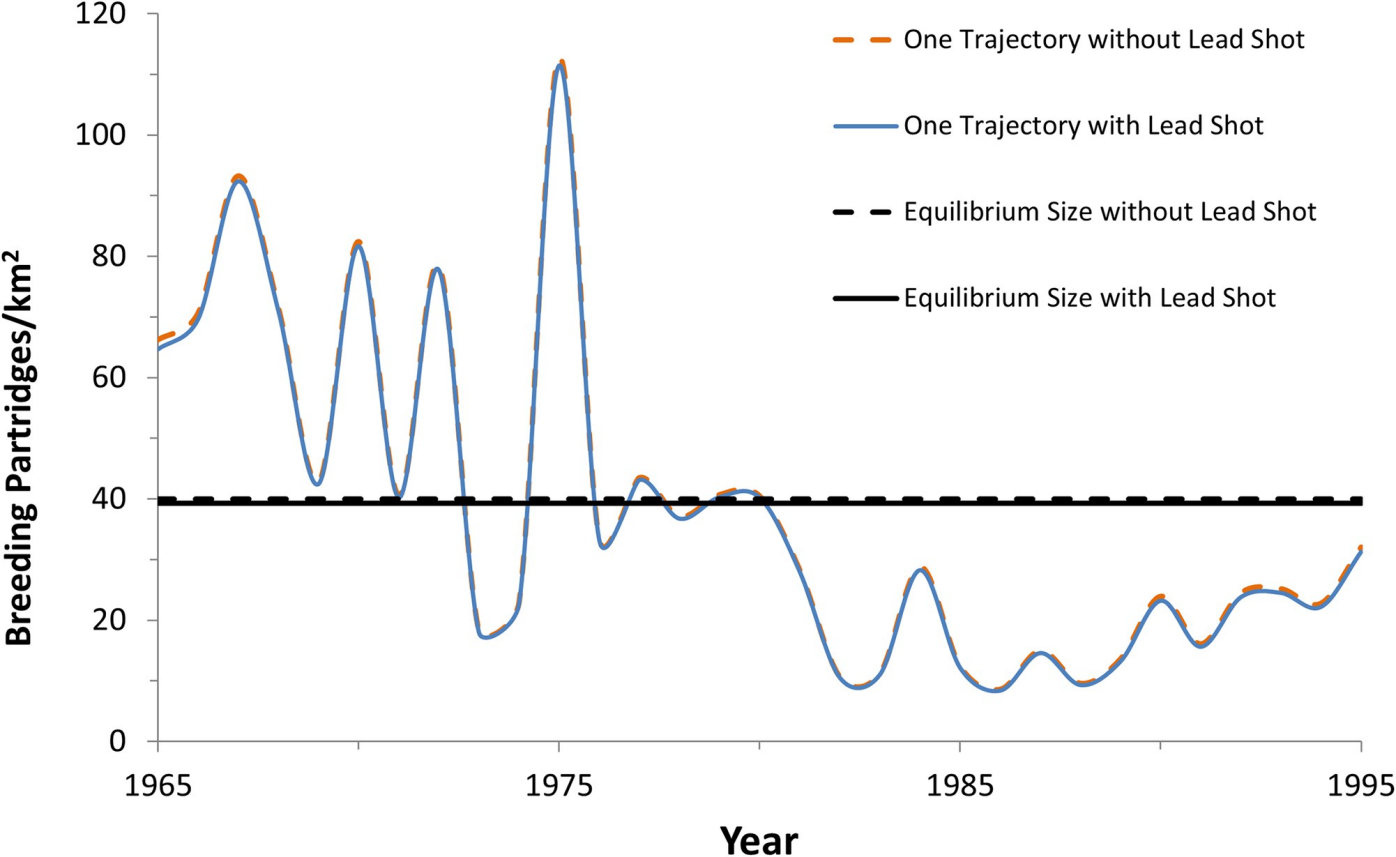
Modeled population trends for grey partridge with and without lead shot ingestion. Horizontal line is mean of 10,000 model iterations, representing the equilibrium population size; and the fluctuating line is an example trajectory similar to the observed declining trajectory in continental Europe [24]. Both show a barely distinguishable difference with and without lead shot. The model included reproductive effects.

The stochastic model output for the grey partridge (representing gallinaceous birds) indicated lead shot ingestion reduced the European gallinaceous breeding pair population size by 1.75% with reproductive effects included and by 1.74% without reproductive effects included (Tables 3 and 4). Not surprisingly, these values are low because the reduction in annual survival (0.41%) and reproduction (0.57%) is low when averaged across Europe. This level of effect, when applied to the continental grey partridge population in the late 1990s, is small relative to the natural variability in this species’ population trend (Fig 3) and likely is not easily discernible using field studies for gallinaceous species, which supports population modeling is needed to identify lead shot ingestion impacts.

**Table 3:** Stochastic population modeling results for lead shot and bullet ingestion with effects on reproduction.

Output Parameter	Without Lead	With Lead	Change in # Due to Lead (and % of pairs lost)
**Gallinaceous Bird Population Model (0.8% poisoned carcasses, 0.32% of birds reproducing less by 17%)**			
**Grey partridge model (representing all declining gallinaceous populations in Europe totaling 12,466,841)**			
Breeding birds/km^2^ in local population at steady state	40.0	39.3	< 1 (1.75%)
Probability of local quasi-extinction to < 5 birds/km^2^	0.13	0.16	0.03
Estimated number gallinaceous breeding pairs "lost" from lead shot ingestion in Europe	**219,936 (1.75% decrease)**		
**Terrestrial Diurnal Raptor Models (5.21% poisoned carcasses, 2.85% of birds reproducing less by 75%)**			
**Common Buzzard Model (representing stable and decreasing small-bodied populations in Europe totaling *404*,*006*–512,606)** ^**a**^			
Breeding pairs/10,000 km^2^ in local population at final steady state	1,656	1,624	32 (1.9%)
Probability of local quasi-extinction to < 350 breeding pairs/10,000 km^2^ in 50 years	0.010	0.019	0.009
**Red Kite Model (representing stable and decreasing small-bodied raptor populations in Europe totaling *404*,*006*–512,606)** ^**a**^			
Breeding pairs/10,000 km^2^ in local population at final steady state	1,693	1,595	97 (5.8%)
Probability of local quasi-extinction to < 350 breeding pairs/10,000 km^2^ in 50 years	0.00	0.00	0.00
**Red Kite Model (representing increasing small-bodied raptor populations in Europe totaling 53,868–*258*,*564*)**			
Breeding pairs/10,000 km^2^ in increasing local population (average of first 50 years)	1,331	1,183	148 (11.1%)
Probability of local quasi-extinction to < 350 breeding pairs/10,000 km^2^ in 50 years	0.0017	0.0064	0.0047
Maximum population growth rate of breeding pairs	1.065	1.050	0.015
**Bearded Vulture Model (representing stable and decreasing large-bodied raptor populations in Europe totaling 39,563)**			
Breeding pairs/60,000 km^2^ in local population at final steady state	194	189	5 (2.7%)
Probability of local quasi-extinction to < 900 birds/60,000 km^2^ in 50 years	0.002	0.004	0.002
**Bearded Vulture Model (representing increasing large-bodied raptor populations in Europe totaling 1,988)**			
Breeding pairs/60,000 km^2^ in increasing local population (average of first 50 years)	190	184	6 (2.9%)
Probability of local quasi-extinction to < 900 birds/60,000 km^2^ in 50 years	0.005	0.031	0.026
Maximum population growth rate	1.080	1.076	0.004
**Raptor Models Combined**			
Estimated raptor breeding pairs/year lost from lead shot and bullet ingestion in Europe	**45,380–*85*,*922* (4.1–7.7% decrease)** ^**b**^		

^a^ Number of buzzards and kites that are stable/decreasing are the total in the trend category divided by 2, which is the reason each species assigned the same number. More recent trends in ranges are *italicized*.

^b^Final range represents weighting by percent of raptor trends in each category (increasing, decreasing, stable) from first period of 2001 to 2012 (lower percent due to fewer increasing populations) to second period of 2013 to 2018 (upper percent due to more increasing populations).

**Table 4:** Stochastic population modeling results for lead shot and bullet ingestion without effects on reproduction.

Output Parameter	Without Lead	With Lead	Change in # Due to Lead (and % of pairs lost)
**Gallinaceous Bird Population Model (0.8% poisoned carcasses)**			
**Grey Partridge Model (representing declining gallinaceous populations in Europe totaling 12,466,841)**			
Breeding birds/km^2^ in local population at steady state	40.0	39.3	< 1 (1.74%)
Probability of local quasi-extinction to <5 birds/km^2^	0.13	0.16	0.03
Estimated number gallinaceous breeding pairs "lost" from lead shot ingestion in Europe	**219,207 (1.74% decrease)**		
**Terrestrial Diurnal Raptor Models (5.21% poisoned carcasses)**			
**Common Buzzard Model (representing stable or decreasing raptor populations in Europe totaling *404*,*006*–512,606)** ^**a**^			
Breeding pairs/10,000 km^2^ in local population at steady state	1,656	1,637	25 (1.1%)
Probability of local quasi-extinction to <350 breeding pairs/10,000 km^2^ in 50 years	0.010	0.016	0.006
**Red Kite Model (representing stable and decreasing small-bodied raptor populations in Europe totaling *404*,*006*–512,606)** ^**a**^			
Breeding pairs/10,000 km^2^ in local population at steady state	1696	1629	67 (4.0%)
Probability of local quasi-extinction to <350 breeding pairs/10,000 km^2^ in 50 years	0.00	0.00	0.00
**Red Kite Model (representing increasing small-bodied raptor populations in Europe totaling 53,868–*258*,*564*)**			
Breeding pairs/10,000 km^2^ in local population when reaches steady state	1,331	1,222	109 (8.1%)
Probability of local quasi-extinction to < 150 breeding pairs/10,000 km^2^ in 50 years	0.0017	0.0056	0.0039
Maximum population growth rate	1.065	1.054	0.011
**Bearded Vulture Model (representing stable and decreasing large-bodied raptor populations in Europe totaling 39,563)**			
Breeding pairs/60,000 km^2^ in local population at final steady state	194	189	5 (2.7%)
Probability of local quasi-extinction to < 900 birds/60,000 km^2^ in 50 years	0.012	0.031	0.019
**Bearded Vulture Model (representing increasing large-bodied raptor populations in Europe totaling 1,988)**			
Breeding pairs/60,000 km^2^ in increasing local population (average of first 50 years)	189	184	5 (2.6%)
Probability of local quasi-extinction to < 900 birds/60,000 km^2^ in 50 years	0.008	0.031	0.021
Maximum population growth rate	1.076	1.077	0.001
**Raptor Models Combined**			
Estimated raptor breeding pairs/year lost from lead shot and bullet ingestion in Europe	**32,655–*59*,*161* (2.9–5.3% decrease)** ^b^		

^a^ Number of buzzards and kites that are stable/decreasing are the total in the trend category divided by 2, which is the reason each species assigned the same number. More recent trends in ranges are *italicized*.

^b^Final range represents weighting by percent of raptor trends in each category (increasing, decreasing, stable) from first period of 2001 to 2012 (lower percent due to fewer increasing populations) to second period of 2013 to 2018 (upper percent due to more increasing populations).

When the 1.75% reduction in population size is applied to a local breeding population size and then scaled up to 676 populations of gallinaceous birds in Europe (not just grey partridges), the gallinaceous bird loss is estimated to be 219,936 breeding pairs or 386,514 breeding birds (Table 3). The reduction was very similar to the loss when reproduction was not reduced (219,207 breeding pairs; Table 4). If only deaths directly caused by lead shot ingestion are included, the estimate is 1.07% with 134,220 breeding pairs lost, which indicates a large proportion of the loss is based on an assumption of sublethal deaths. The upper bound estimate, assuming every sublethal concentration resulted in death (and therefore none could have reduced reproduction), was 2.4% with 307,383 breeding pairs lost. The probability of quasi-extinction of local gallinaceous populations on average in Europe decreased from 16 to 13% when we removed lead shot ingestion.

For the single-species evaluation of the grey partridge, the bounded range of percent of carcasses with direct and possible ultimate losses was 0.06 to 0.15%, with a midpoint of 0.11% (Table 5, S3 Table). This range is lower than the midpoint of 0.8% for the group of gallinaceous birds (Table 2) because most of the grey partridges are in France, which has lower exposure. The modeled reduction in the grey partridge population size in Europe was 0.9% (Table 5). When scaled up to Europe, the grey partridge population loss is estimated to be 14,512 breeding pairs.

**Table 5:** Stochastic population modeling inputs and results for individual species affected by lead shot and bullet ingestion including and excluding potential effects on reproduction.

Species and its Population Trend in Europe from 2000 to 2018	Potential lead-poisoned % of carcasses (midpoint)	% with reproduction reduced	% breeding pair reduction	Estimate of breeding birds lost in Europe in 2012	Increase in quasi-extinction probability
Grey Partridge, Declining	0.11	0.0	0.953	14,512	0.02
	0.11	7.3	0.948	14,589	0.02
Red Kite, Stable	1.37	0.0	0.9	224	0.00
	1.37	0.77	1.4	341	0.00
Red Kite, Increasing	1.37	0.0	1.6	398	0.0005
	1.37	0.77	2.2	547	0.0007
Common Buzzard, Decreasing to Stable	4.85	0.0	1.4	9,409	0.007
	4.85	5.1	2.3	15,458	0.024
Bearded Vulture, Increasing	5.3	0.0	2.7	5	0.003
	5.3	1.9	2.9	5	0.003

### Raptor modeling results

**Fig 4:**
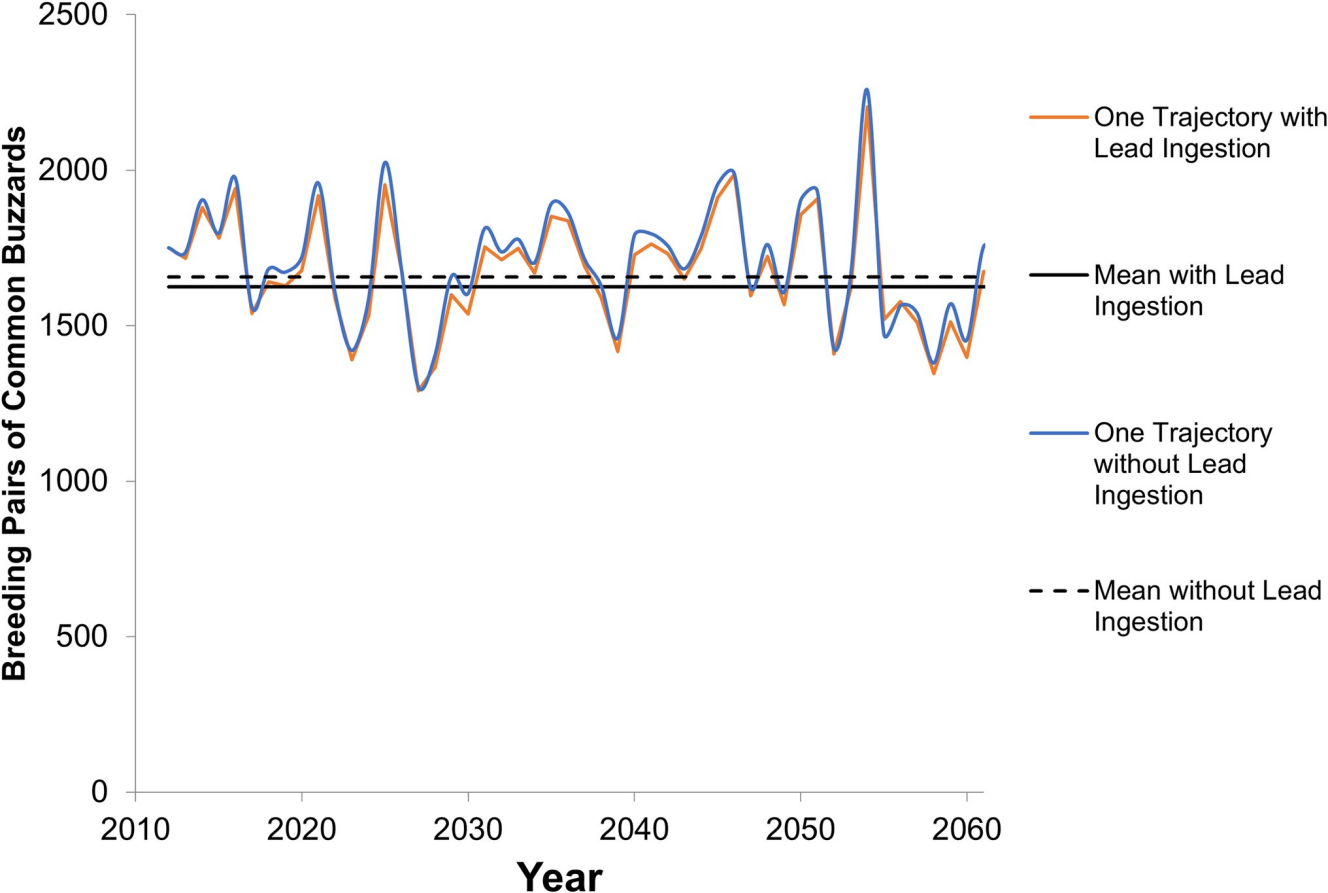
Modeled population trends for common buzzard with and without lead shot and bullet ingestion. Shown with reproductive effects. In addition to the mean, an example iteration of one of the 10,000 runs is shown to demonstrate the trend variability.

**Fig 5:**
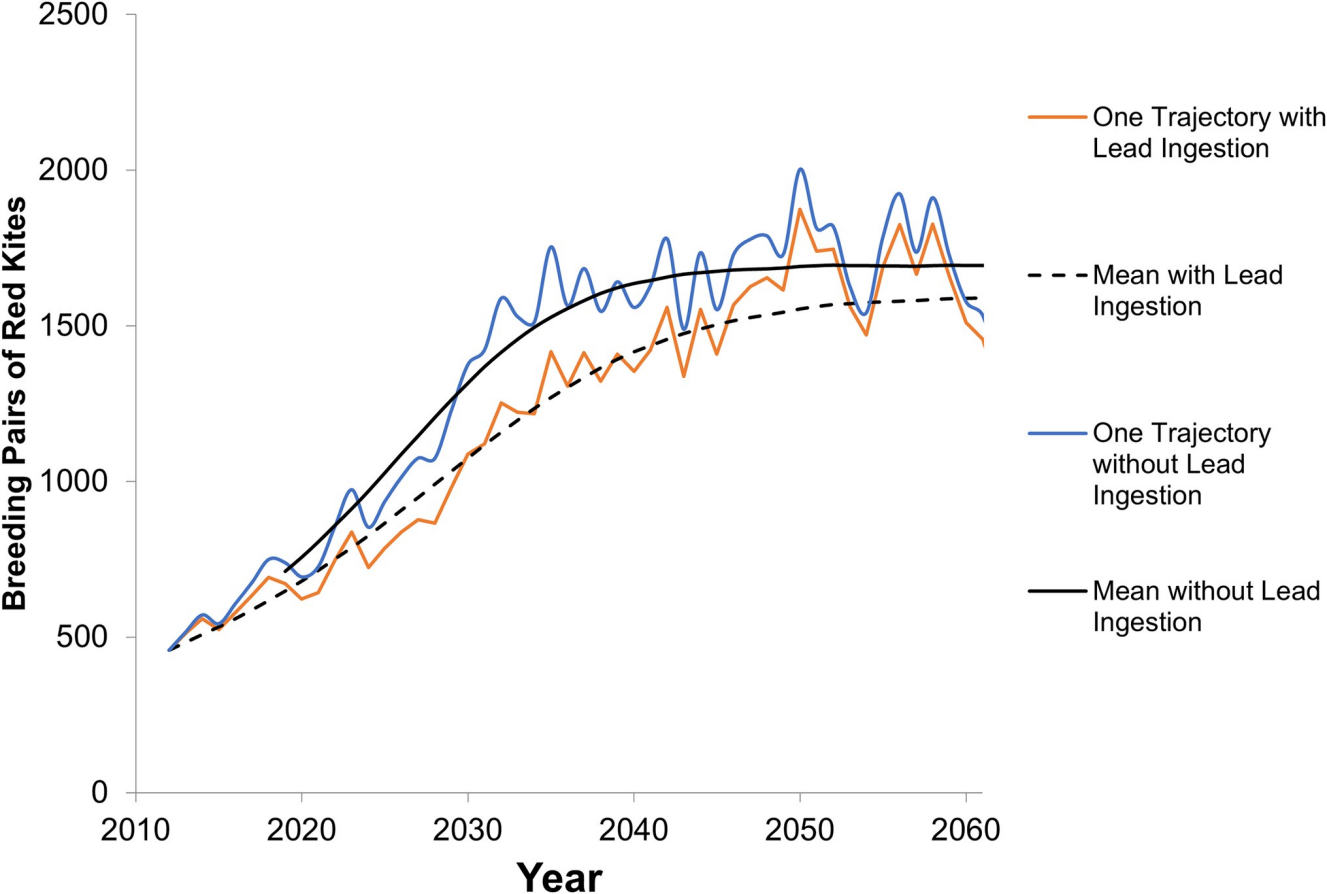
Modeled population trends for red kite with and without lead shot and bullet ingestion. (a) Shown with reproduction effects. In addition to the mean, an example iteration of one of the 10,000 runs is shown in to demonstrate the trend variability.

The stochastic model output indicated that, in the scenario with reproduction reduced, lead shot and bullet ingestion reduced the population steady state equilibrium size by 1.9% for species represented by the common buzzard and by 5.8% for species represented by the red kite population when it reached steady state (Table 3, Figs 4 and 5). During the growth phase for the red kite representatives after 5 years but prior to reaching steady state, the reduction ranged from 6.2 to 18.3%, averaging 11.1% over the first 50 years (Fig 5). The reduction in reproduction comprised 28 to 42% of the loss (Tables 3 and 4). The population size reduction, whether with or without reproductive effects, was smaller than or similar to the natural variability in the population size of these small-bodied raptors (Figs 4 and 5) and may be difficult to detect in field evaluations. These reductions, based on Europe-wide estimates of impacts to small-bodied raptors, are assumed to be applicable to small-bodied raptor populations having the same population trend directions.

**Fig 6:**
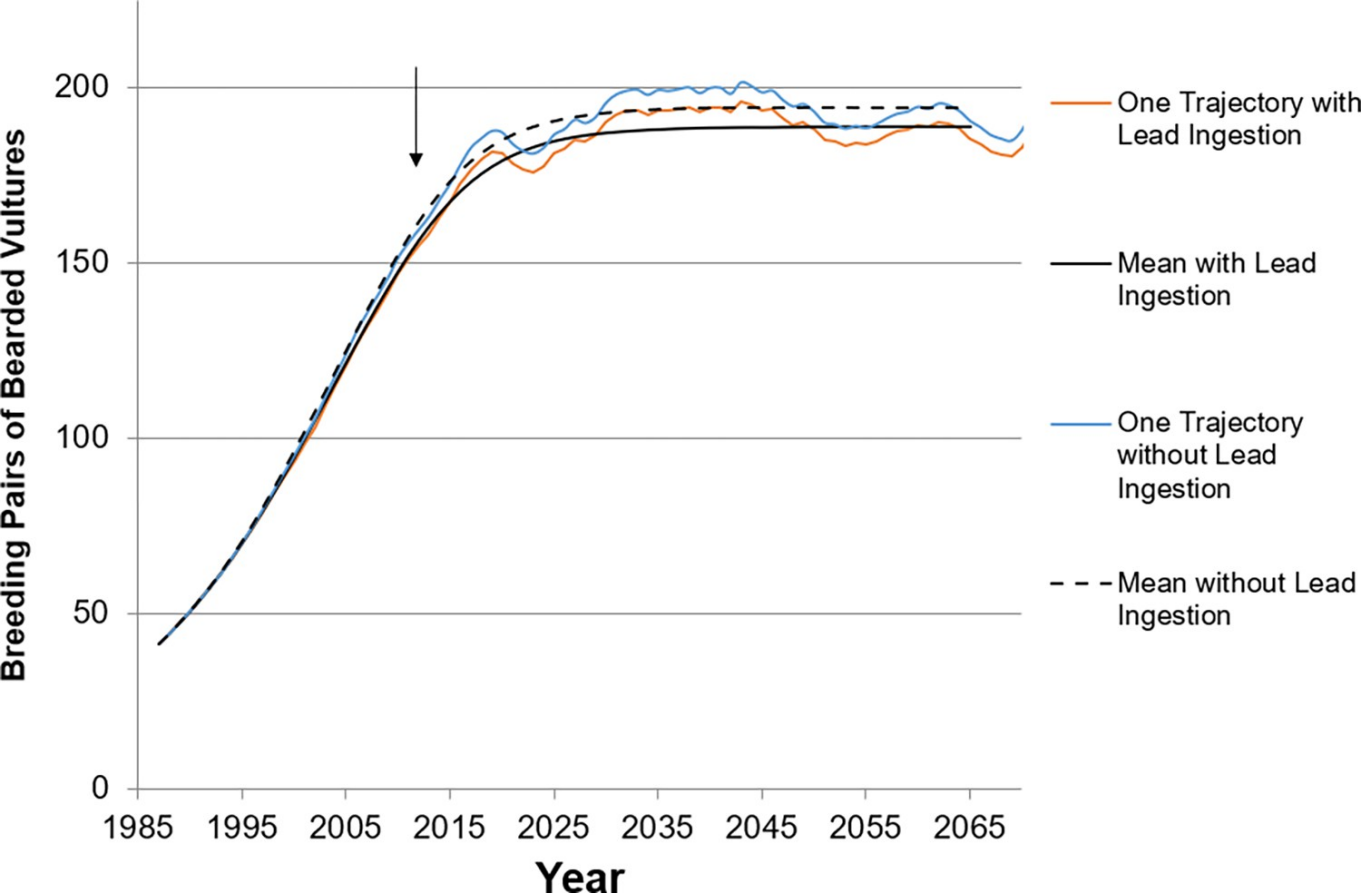
Modeled population trends for bearded vulture with and without lead shot and bullet ingestion. (a) Shown with reproduction effects. In addition to the mean, an example iteration of one of the 10,000 runs is shown in to demonstrate the trend variability. Analysis of lead effects began in 2012 (arrow).

These population reductions were also similar to the natural variability for the bearded vulture also, representing increasing populations of large-bodied raptors (Fig 6). During the population growth phase for the bearded vulture, the average reduction was 2.9%, ranging from 2.7 to 3.2%. After the population reached steady state, the reduction was 2.7% (Table 3). Mortality was the main driver of the reduction, with reproduction effects accounting for ≤ 10% of the reduction, depending on whether the population was stable or increasing (Table 4).

These raptor population size reductions were applied to the total breeding population size for all diurnal raptors susceptible to lead ammunition ingestion in Europe by calculating the weighted average reduction in size for European populations, first assigning a 96% weighting to the mean of the buzzard and red kite reduction estimates for stable or decreasing small-bodied raptor populations and 4% weighting to the red kite reduction estimates for increasing small-bodied populations. These weightings are based on the percentages estimated from the 2000 to 2012 short-term trends. We completed the same process using weightings from the 2013 to 2018 short term trends. The weighted average reduction for lead ammunition ingestion of the two species combined with reproduction reduced was 4.1% and 7.9% for the first and second short term periods, respectively. When combined with large-bodied raptors, weighted by the number of large and small-bodied raptors in Europe, we estimated a 4.1 to 7.7% reduction in breeding pairs lost over 50 years due to lead ammunition ingestion when reproduction was reduced, and 2.9 to 5.3% reduction when reproduction was not affected (Tables 3 and 4). When scaled up to the combined small-bodied (1,077,350) and large-bodied (39,792) European raptor populations, the loss estimate that includes reduced reproduction is 45,380 breeding pairs based on the first period trends and 85,922 for the second period. Excluding reproductive effects, the loss estimate is 32,655 to 59,161 breeding pairs based on the trends during the two time periods (Table 4). Using only direct cause of deaths for lead impacts, the raptor breeding pair loss range is 17,555 to 28,206 (1.6 to 2.5% reduction), which is approximately half of our midpoint-based estimates without reproduction reduced, indicating our estimates are strongly influenced by the assumption of sublethal mortality. The upper bound estimate, assuming all birds with liver concentrations exceeding the subclinical threshold died (and did not reproduce) is a 5 to 8.5% reduction, equal to 55,956 to 94,577 pairs. Notably, as populations became more robust, producing more increasing trends in Europe in the recent near term, numeric bird losses to lead poisoning increase, indicating this metric may not be as important as other metrics evaluating the population viability such as population growth or extinction rates.

When the modeled raptor populations were exposed to lead ammunition ingestion, the probability of quasi-extinction changed very little. The likelihood of lead-impacted populations decreasing to < 350 pairs, or for bearded vultures to < 900 individuals, was less than or equal a few percent, highest for the bearded vulture model (Tables 3 and 4).

For the single species evaluation of common buzzards and red kites, more assumptions were required to estimate losses because of more data gaps on lead exposure by species for country groups. Lead ammunition ingestion data were missing for Norway and Sweden for the common buzzard. The upper and lower bound percent estimates for the German country group (e.g., groups in Table 2) were applied to that group (result is similar if birds in those Scandinavian countries were not included in weighting). The average of France and UK was used for the missing upper bound estimate of the Spain/Portugal group. For the red kite, Denmark and Belgium were grouped with the UK group, and all other countries with red kite as a resident were grouped with the German group (S3 Table).

For the individual raptor species inputs into the population models (with reproduction reduced), the bounded ranges of percentage of carcasses with direct and possible ultimate losses in Europe from lead ammunition ingestion were 0 to 9.7% (with a midpoint of 4.9%%) for the common buzzard and 0.6 to 2.1% (with a midpoint of 1.4%%) for the red kite. The bearded vulture range was 3.4 to 7.1%, with a midpoint of 5.3% (S3 Table). The percentage estimated to have reduced reproduction ranged from 0.8% (red kite) to 5.1% (common buzzard; Table 5).

For individual raptor species, the mean reduction in population size over 50 years was estimated using an increasing population model if the European population was increasing and using the steady state model if the population was stable or decreasing in the short-term periods between 2000 and 2018 (Table 5). The mean reduction in population size was 2.3% for the initially decreasing and more recently stable European common buzzard population and 2.2 and 4.0% for the stable and more recently increasing red kite European populations, respectively. The reduction was 2.9% for the increasing European bearded vulture population. These species-specific percentage reductions were slightly higher for the buzzard, about a third lower for the red kite, and almost the same for the bearded vulture when compared to reductions using the species’ model as a surrogate with input of the weighted, aggregated exposure percentage from all the evaluated raptor species. The red kite differed the most from the Europe-wide estimate, probably because it resides year-round in a subset of countries where the exposure percentage was less than our estimate across Europe. Alternatively the missing data in more countries may be affecting the results.

Overall, estimates of annual reductions in population size of breeding pairs for each raptor individual species ranged from over 15,000 for the most abundant raptor, the common buzzard, down to 5 per year for the recovering bearded vulture. The results from the individual species evaluations suggest that the weighted aggregation of species to estimate European-wide losses may actually overestimate losses of individual species, because the aggregated 5.21% is close to the highest estimate for the four individual species evaluated (5.3%), at least for these species that had enough data to attempt to evaluate on a species level. The high aggregated percentage is driven by the estimates in mountainous regions and in Scandinavian countries (Table 2), where big game hunting is common.

## Discussion

The purpose of this paper is to demonstrate a method to estimate the magnitude of lead ammunition ingestion effects on highly susceptible terrestrial bird populations in Europe. Pain et al. [5] suggest it is too difficult to measure strength and form of density-dependence in birds to analyze lead effect with any precision using population models. We disagree because long-term datasets in Europe demonstrate that gallinaceous and raptor bird populations can increase from low numbers to carrying capacity (especially raptors in Europe), and such trends have been used to develop reasonable density-dependent relationships that can be incorporated into population models (e.g., [14, 49, 52]). These relationships have been used to inform game harvest and are important for conservation management.

Our population modeling approach produces estimates of parameters important for managing stressors that might affect a population’s viability, including percent reduction in population size, probability of quasi-extinction, and change in population growth rate from the stressor, which in this analysis is lead poisoning. The results indicate greater numeric losses when populations are growing (i.e., higher losses resulted using 2013–2018 short term period trends that had more increasing populations) or when a species has higher reproductive rates (gallinaceous birds vs. raptors). The number of birds lost and even the percentage reduction in the population size when a population is growing might be of interest [9, 10 and used in ECHA [1] but is not as crucial as understanding change in population sustainability [13]. A management goal for vulnerable species is to have increasing and sustainable avian populations. Success in that goal will produce higher numbers of birds lost to lead poisoning, which is why numeric losses should be placed in the context of sustainability.

Sustainability is best evaluated using the probability of quasi-extinction or population growth rate [13–15]. If a population reverses from stable to decreasing from lead ingestion, number of birds lost will actually decrease, but the lead poisoning becomes more important to population viability. Due to its life history strategy of long lifespan and delayed sexual maturity (≥ 6 years compared to 2 to 3 years for the smaller-bodied raptors), the bearded vulture and similar large-bodied raptors might be expected to have the lowest population growth rate and highest percentage population reduction due to lead poisoning [12]. However, these populations are still increasing, and our results, when applying the same exposure level to all three species, indicated the species represented by the red kite would have the greatest reduction in population size from lead poisoning. This result is likely because the red kite maximum geometric growth rate (λ = 1.065), based on a higher-elevation habitat in Wales with low food productivity [14], is lower than the estimate for large-bodied raptors based on the bearded vulture population (λ = 1.076). Also, its rate is much lower than the maximum growth rate of species represented by the common buzzard (λ = 1.20) and grey partridge (λ = 1.589) populations. The large-bodied raptor results indicate relatively low numbers of birds are lost to lead poisoning compared to the more abundant smaller bodied hawks and kites, because of small population sizes of eagles and vultures in Europe. However, because of their relatively low population sizes (39,762 large vs. 1,077,350 small-bodied raptors), these larger species have a higher risk of extinction (highest quasi-extinction of the raptor models, Table 3).

Our results indicate that, not only is life history important to consider, but also habitat quality. Interestingly, the red kite population in Wales has recently expanded into more productive habitat and has switched to now having a higher maximum geometric growth rate (1.1) than the maximum growth rate for the bearded vulture population [14, 53], increasing its recovery rate. This underscores the importance of evaluating habitat quality, a factor we did not initially consider that affects the magnitude of lead effects in bird populations across Europe and changes as the populations expand. Our aggregated results for small-bodied raptors actually represent an average for areas with poor (red kite) and good (common buzzard) quality habitat. Our approach could be improved by stratifying by acres of habitat quality when scaling up to Europe, if such information becomes available.

We also evaluated effects of lead by individual species, which, for the raptors, means exposure (% of carcasses with lead poisoning) varied for the three species rather than being held constant. In that analysis, the red kite had a lower reduction in population size than the bearded vulture. The bearded vulture and other raptors that are restricted to feeding in the mountains in big game hunting areas (where bullets are also used) appear to be more exposed to lead ammunition ingestion than species in other areas (Fig 2). For gallinaceous species, the individual grey partridge exposure was lower than the exposure when pooled across species, because data suggest France, where most grey partridges occur, had a lower percentage of carcasses with lead diagnoses. Because of lower sample sizes across countries, exposure estimates for individual species can be less certain than when the objective is to evaluate all species in Europe combined in proportion to their abundance and trends, with the latter only true if the pooled samples are generally in proportion to species abundance and collections are not biased. These criteria are uncertain.

### Comparison to other methods

Our analysis differs from the Bellrose [8] method used by Pain et al. [9] for gallinaceous birds, because we used readily-available carcass data in Europe and we reported key metrics for managing populations. Most importantly, our method requires fewer assumptions based on professional judgment. Pain et al. [9] estimated “hundreds of thousands” of terrestrial game birds may die from lead poisoning annually in the UK. Their estimate is based on the proportion of hunter-shot gallinaceous birds that had ingested at least one lead shot in the gizzard when sampled, corrected for (1) an assumed uncertain hunter bias factor of either 1.5 [9] or 2 [10] to account for lead-weakened birds being easier to shoot, (2) an assumed turnover rate in the gastrointestinal tract of 20 days during the hunting season period that was based on waterfowl, and (3) an added mortality rate arbitrarily assumed to be half that of waterfowl in the USA ingesting one lead shot. They stated their estimate also excluded sublethal effects, although Bellrose’s mortality tracking in the field for waterfowl in the USA included sublethal effects. To validate these assumptions, the Bellrose [8] series of calculations, further evaluated in Green and Pain [54] for validity, require ringing dosed birds that are released and then waiting many years to evaluate the added mortality from direct and indirect effects of lead shot ingestion.

Rather than waiting for results from such field studies, our method can assist in immediately evaluating their assumptions. For terrestrial game birds, Pain et al. [9] estimated the added annual mortality proportion was 0.045, resulting in an estimate of 0.56% and 0.32% of pheasant and red-legged partridge populations dying from lead shot ingestion annually in the UK, respectively [9]. We found that, using the first step of our method, UK red-legged partridge and pheasants annually dying is 0.4%, which falls within the range of the Pain et al. [9] estimates (see S1 Appendix for the calculation equations). This comparison indicates their professional judgment assumptions for added mortality and other parameters of these gallinaceous birds in the UK may be reasonable. This same metric applied to all of Europe is 0.2% (using first step or our method, see S1 Appendix for more discussion of metric differences between methods). This estimate is lower than the ECHA [1] more arbitrarily selected 1% estimate that they used to estimate population losses without accounting for among-country differences in exposure or population modeling. The metric of the proportion dying is actually an input variable to our population model, which incorporates stochastic and density-dependent vital rates that vary relative to whether the population is at or increasing toward its current equilibrium at carrying capacity [14, 49], which in turn can be reduced by stressors such as lead ingestion. We estimated the percentage reduction in the European population size of gallinaceous breeding pairs to be 1.74 to 1.75% of the susceptible gallinaceous birds with and without reducing reproduction, respectively, a result that is higher than the 0.2% dying. This difference demonstrates the importance of using a population model to estimate population size reductions.

The Bellrose [8] method also does not address reproductive effects, nor do most avian lead poison studies that model population-level effects [11, 13, 15]. We are the first to identify a method of evaluating, or at least bounding, such effects for gallinaceous birds and raptors, while recognizing that more field and laboratory studies are needed to assess relationships between reproduction and gizzard lead shot or lead in tissue [14]. Because field data are very limited, our reproductive analysis is provided only as a demonstration. Maternal transfer of lead to bird eggs and chicks occurs [55] but is considered relatively low [47, 56–58], which likely limits occurrence of impacts to egg production and hatching success to the high end of sublethal doses for gallinaceous birds (e.g., 3 lead shot in gizzard [36]). Lead-impacted adults of gallinaceous birds and raptors might also reduce their time incubating and caring for young in the wild, and chick behavior can be altered if chicks directly ingest lead, leading to higher chick mortality [59]. However, lead concentrations in blood of raptors and their nestlings are generally low relative to during the hunting season [60], which is why we excluded the nestlings. A laboratory study indicated effects of high lead concentrations in livers of American kestrels did not affect clutch size or eggshell thickness [55]. Potts [26] reported 13 to 14 lead shot in gizzards of two grey partridge chicks that appeared to be healthy. These findings as well as the limited laboratory studies of raptors create uncertainty on the extent reproduction is affected in the wild populations of these two groups of birds.

As we demonstrated, our approach for evaluating raptor impacts is to use poisoning percentages based on the actual cited and published cause of death as a lower bound and subclinical liver exceedance percentages as an upper bound as inputs to the population model. Very recently (after the ECHA [1] report became public), Green et al. [12] evaluated raptor losses in Europe from lead ingestion using actual or modeled lead liver concentrations based on correlates with hunter density to modify survival in population models. Their method differs from ours in that they used clinical liver concentration exceedances as their best estimate of actual deaths and did not evaluate if birds with exceedances died from another cause. For example, our analysis, using empirical rather than modeled data, indicates common buzzards have not been diagnosed as directly dying of lead poisoning in any reports we could find, which indicates the deaths for buzzards in Green et al. [12] probably are mostly from other causes but could have been ultimately caused by lead poisoning weakening the bird. Our approach more explicitly addresses and bounds direct and indirect (sublethal) causes of deaths. Including necropsy diagnoses in the lower bound estimates can produce different results than the solely tissue-based results in Green et al. [12]. Consequently, unlike the results in Green et al. [12], our midpoint estimates between the lower and upper bound based on liver tissue do not correlate well with hunter density because birds with clinical lead concentrations may die of illegal poisoning or shooting first, and those deaths may be unrelated to hunter density.

Our population modeling approach also differs from Green et al. [12] but is similar to others [11, 13] that focus on models built and calibrated on real population data and trends. However, we then use those models as surrogates for similar species with similar trends, whereas Green et al. [12] developed 10 species-specific but uncalibrated models starting at equilibrium for each terrestrial and wetland-feeding raptor with relatively high lead exposure. They did not incorporate stochasticity. An advantage of our approach is increased realism. A disadvantage is that life histories and trends of some species may not be well-represented by one of the four models we were able to construct. The advantage of using the Green et al. [12] uncalibrated models is that the models are generic enough to apply to many species using assumed relationships based on known life history traits. A disadvantage is that the actual trend direction and growth rate are ignored. Thus, extinction probability and growth rate change are unknown. Additionally, the shape of the density dependence is unknown, which strongly affects the results, as Green et al. [12] demonstrated (e.g., theta > 1 reduces lead impacts and long-lived raptors are likely to have theta > 1, [13]). Green et al. [12] also used population sizes that differ from our estimates from 2012, making it difficult to compare total bird reductions for individual species. However, their equilibrium model percent estimate for common buzzard population size reduction is similar to our estimate (1.5 vs. 1.4%). Their estimates are higher for the red kite (3.2 vs. 0.9%) and bearded vulture (4.0 vs. 2.9%) but are in the same general order of magnitude. Together, both approaches can provide insight into avian lead poisoning from ammunition in Europe, and both identify species with lower maximum population growth rates as the most vulnerable species.

For other individual species that feed only terrestrially that we did not model, Green et al. [12] reported the highest estimates of population reduction from lead poisoning were for the griffon vulture and golden eagle, which appears to primarily be because they have high exposure relative to other species. Over 90% of the griffon vultures are in Spain, and the source of lead is a mixture of soil and ammunition [36], making it difficult to interpret the portion of the mortality from ammunition ingestion alone for this species. Griffon vultures may be more tolerant of high lead blood concentrations than other raptors (asymptomatic despite severe clinical levels in blood [32, 36]). To evaluate this possibility, we calculated the weighted proportion of griffon vulture carcasses that pathologists reported as dying directly of lead poisoning from our dataset, and it was small at 0.01%. The golden eagle may be less tolerant [32]. Unfortunately, half of the golden eagle population of 6,991 breeding pairs in Europe (in 2012) occurred in countries that lack diagnoses of cause of deaths or liver concentrations. If most of the countries without data on this species are assumed to have the same exposure as in the mountainous Alps, this species has a high lead poisoning exposure of 12% (lower bound) to 14% (upper bound), much higher than the bearded vulture.

#### Areas requiring additional research

Our results suggest that future research should focus on developing and calibrating more population models to actual bird population trends to evaluate effects on individual terrestrial species in different habitat quality areas with high exposure to lead shot or bullet fragments. In particular, more carcass data with population modeling of the golden eagle is required to better understand the magnitude and effects of its lead poisoning, and the lead source.

Our method assumes half of the sublethally-affected birds die from ingesting lead ammunition, leaving the other half to experience reduced reproduction. The percentage that actually die from other proximal causes exacerbated by sublethal effects of lead is not known, and the subclinical threshold we used for our analysis of ingestion effects is uncertain. Berny et al. [61] has discussed that healed wounds with embedded lead shot in raptors also may be responsible for some of the sublethal concentrations in the liver, which we did not evaluate (but is included in tissue concentrations in our analysis). Ecke et al. [19] suggest the sublethal threshold for liver is lower than we used, at 0.025 mg/kg dw. Their threshold is based on decreased mean flight height and movement rate of golden eagles with higher lead concentrations in blood that increase during the hunting season. An uncertainty in Ecke et al. [19] that they discuss is that the flight results could reflect a change in foraging patterns to a lower height and shorter distances in order to find the hunter-discarded offal that become more available as the hunting season progresses. In contrast, Poessel et al. [62] did not find that sublethal lead concentrations in blood were correlated with flight behaviors.

For gallinaceous birds, we used a sublethal threshold of at least one lead shot in the gizzard of a dead bird, assuming it corresponds to subclinical levels in the liver. Because this assumption is uncertain, more liver concentrations in necropsied gallinaceous birds are needed to improve estimates. Also, our European dataset of direct deaths, compiled to identify data gaps, includes studies that performed lead toxicology tests on only a subset of birds, usually those suspected of poisoning, or on no birds if no signs of lead toxicosis were obvious during necropsy. The use of this dataset may underestimate deaths directly caused by lead poisoning if pathologists misdiagnosed the proximate cause of death. Future studies and necropsy reports should be more explicit as to the uncertainty in their diagnoses. Lastly, necropsy and liver concentration data are missing or limited for many countries, which are needed to improve the Europe-wide estimates.

In addition to terrestrial birds, our approach can be applied to wetland or waterfowl-feeding raptors and other wetland birds. To identify the effect of the terrestrial contribution to lead poisoning in species such as the white-tailed eagle (*Haliaeetus albicilla*) and Spanish imperial eagle (*Aquila adalberti*), such a study would have to focus on areas where the birds feed on non-wetland prey or discarded big game carcasses and offal. Future research also could apply our proposed method to waterfowl throughout Europe and then compare results to estimates using the Bellrose [8] method. Instead of focusing on lead shot ingestion rates in living waterfowl populations (e.g., sampled by hunting or trapping) and applying hunting and turnover correction factors, this work would require tabulating the percentage of found waterfowl carcasses that were diagnosed as dying from lead shot ingestion based on necropsy reports in different countries. Those percentages would still have to be corrected for the number of carcasses that died from hunting (i.e., unavailable to be found because they were removed from the wild), to ensure all causes of deaths are included in the estimated death percentages. However, to be fully comparable, the Bellrose [8] method would have to be modified by entering the estimated added mortality and potential reproductive losses into waterfowl population models to predict population-level reductions.

The sources of lead poisoning from soil or ammunition, and the contribution of lead shot compared to lead bullet poisoning, in raptors also need further investigation. Some of the sublethal concentrations in liver we used may have been from lead contamination in soil, which currently can only be determined by isotopic analysis, which is not performed in most studies. For the contribution of lead shot only as to compared with bullets, we investigated a preliminary method of estimating the contribution based on hunting practices and food sources in the S1 Appendix. The analysis indicates lead shot ingestion alone might reduce raptor population sizes in Europe on average by 2.1 to 3.3%, which is approximately half the reduction we estimated from all lead ammunition.

Although we apply assumptions to countries where data gaps exist, our suggested method could help refine estimates for decision-making. Although our results using the proposed method are preliminary because of data gaps and uncertainties, they suggest that lead ingestion is not impacting the sustainability of the European terrestrial bird populations as a whole. However, some species such as the golden eagle need further investigation. Lead poisoning is causing decreases in population sizes and is slowing recovery of some raptor populations, particularly populations in poorer-quality habitats. Concern appears less for gallinaceous birds, because the current data suggest that gallinaceous bird loss from lead shot ingestion is relatively low. Finally, other stressors are strongly impacting European gallinaceous and raptor populations (e.g., S2 Appendix and [14]), and we recommend those stressors be analyzed and modeled using the same approach we have proposed herein for lead poisoning.

## Supporting information

S1 Table Collected dead gallinaceous birds estimated to have directly or ultimately died of lead poisoning. Ultimate estimates are based on at least one lead shot in gizzard or sublethal liver lead concentrations and are assumed to be an upper bound of possible deaths.
(DOCX)

S2 Table Collected dead raptors estimated to have directly or ultimately died of lead poisoning. Ultimate estimates are based on liver lead concentrations and are assumed to be an upper bound of possible deaths.
(DOCX)

S3 Table Collected grey partridge, common buzzard, red kite, and bearded vulture carcasses estimated to have directly or ultimately died of lead poisoning. Ultimate estimates are based on liver lead concentrations and are assumed to be an upper bound of possible deaths.
(DOCX)

S1 Appendix Methods for compiling data in the literature. (DOCX)

S2 Appendix Bearded vulture model development. (DOCX)
