# Supplementary material for: Method to assess the potential magnitude of terrestrial European avian population reductions from ingestion of lead ammunition
Source: PLoS One. 2022 Aug 29;17(8):e0273572. doi: 10.1371/journal.pone.0273572 (PMC9423653; doi:10.1371/journal.pone.0273572)
Supplement: S1 Table — Ultimate estimates are based on at least one lead shot in gizzard or sublethal liver lead concentrations and are assumed to be an upper bound of possible deaths. (DOCX) [file pone.0273572.s001.docx]

**S1 Table. Collected dead gallinaceous birds estimated to have directly (cited as cause) or possibly ultimately (exceedance of sublethal lead concentrations) died of lead poisoning.** Ultimate estimates (lethal plus sublethal deaths) are based on at least one lead shot in gizzard or sublethal liver lead concentrations and are assumed to be an upper bound of possible deaths. This table presents data plotted in Fig. 1 and summarized as percentages by country in Table 1. Studies that included telemetry to track cause of mortality are bolded.

| **Country** | **Common Name** | **Species** | **No. Carcasses Evaluated for Cause of Death** | **No. Carcasses Evaluated that had Reported Tissue Concentrations** | **% with Ingested Lead Shot Cited as Cause of Death** | **% that are Potentially Lethal and Sublethal Lead-caused Deaths based on Shot/Tissue** | **Years Collected** | **Source** |
| --- | --- | --- | --- | --- | --- | --- | --- | --- |
| UK (Sussex County) | Gray Partridge | *Perdix perdix* | 177 (+59) | 0 | 3.8 | -- | 1952-2011 | Potts 2012^a^ |
| UK (Sussex County) | Red-legged Partridge | *Alectoris rufa* | 52 (+19) | 0 | 2.8 | -- | 1952-2011 | Potts 2012^a^ |
| UK (southeast) | Gray Partridge | *Perdix perdix* | 446 (+149) | 446 | 2.5 | 4.5 | 1963-1992 | Potts 2005^a^ |
| UK (northern England, Scotland) | Red Grouse | *Lagopus lagopus scotica* | 729 | 196 | 0 | 1.3 | 1999-2001, 2003 | Hudson et al. 1997,  Thomas et al. 2009 |
| UK (south, east England) | Gray Partridge | *Perdix perdix* | 198 | 0 | 0 | -- | 2004-2006 | **Buner et al. 2011** |
| UK (East Anglia) | Common pheasant | *Phasianus colchicus* | 75 | 0 | 0 | -- | 2011-2013 | **Draycott 2013** |
| UK (throughout) | Red-legged Partridge | *Alectoris rufa* | 503 (+186) | 503 | 0.1 | 0.2 | 1963-1992 | **Butler 2005^a^** |
| UK (Scotland) | Gray Partridge | *Perdix perdix* | 138 | 0 | 0 | -- | 1997-2003 | **Parish and Sotherton 2007** |
| France (northcentral) | Gray Partridge | *Perdix perdix* | 80 | 0 | 0 | -- | 1995-1997 | **Bro et al. 2001** |
| France (south - Pyrenees) | Gray Partridge | *Perdix perdix* | 67 | 0 | 0 | -- | 1992-2001 | **Besnard et al. 2010** |
| France (northcentral) | Gray Partridge | *Perdix perdix* | 261 | 0 | 0 | -- | 2010-2011 | **Millot et al. 2015, Bro et al. 2013** |
| France (throughout) | Gray Partridge | *Perdix perdix* | 95 | 0 | 0 | -- | 1995-2014 | Millot et al. 2017 |
| Denmark (pre-ban throughout) | Common pheasant | *Phasianus colchicus* | 199 | 199 | 0 | 0 | 1971-1977 | Clausen & Wolstrup 1979 |
| Denmark (pre-ban throughout) | Gray Partridge | *Perdix perdix* | 62 (+21) | 62 | 1.2 | 1.6 | 1971-1977 | Clausen & Wolstrup 1979^a^ |
| Spain (southern and central) | Red-legged Partridge | *Alectoris rufa* | 151 | 0 | 0 | -- | 2000-2012 | **Buenestado et al. 2009** |
| Spain (northwestern) | Red-legged Partridge | *Alectoris rufa* | 80 | 0 | 0 | -- | 2008-2009 | **Gaudioso et al. 2011** |
| Spain (southwestern) | Red-legged Partridge | *Alectoris rufa* | 0 | 71 | -- | 1.9 | 2000 | Soler-Rodriguez et al. 2004, Ferrandis et al. 2008 |
| Spain | Red-legged Partridge | *Alectoris rufa* | 0 | 219 | -- | 1.6 | 2016-2018 | Romero et al. 2020 |
| Spain | Barbary Partridge | *Alectoris barbara* | 0 | 13 | -- | 6.9 | 2016-2018 | Romero et al. 2020 |
|  |  |  |  |  |  |  |  |  |
| **S1 Table. Continued.** |  |  |  |  |  |  |  |  |
| **Country** | **Common Name** | **Species** | **Carcasses (n, direct)^a^** | **Carcasses**  **(n, ultimate)** | **Reported (direct %)** | **Maximum (ultimate %)** | **Years  Collected** | **Source** |
| Switzerland | Gray Partridge | *Perdix perdix* | 85 | -- | 0 | -- | 1998-2000 | **Buner and Schaub 2008** |
| Germany (northwestern) | Common pheasant | *Phasianus colchicus* | 190 | -- | 0 | -- | 2011-2014 | Curland et al. 2018 |
| Hungary | Common pheasant | *Phasianus colchicus* | 24 | -- | 0 | -- | 1989-1993 | Ákoshegyi 2000 |
| Norway (northern-during ban) | Willow Ptarmigan | *Lagopus lagopus* | 0 | 18 | -- | 0 | 2009-2011 | Reitan 2013 |
| Italy (central) | Gray Partridge | *Perdix perdix* | 31 | 2 | 0 | 0 | 2000 | **Meriggi et al 2002** |
| Italy (north) | Common pheasant | *Phasianus colchicus* | 0 | 2 | -- | 0 | 1994-1995 | Alleva et al. 2006 |
| **EU** | **All birds** | **All birds** | **2403** | **1731** | -- | -- | -- | -- |

^a^ Added birds dying from hunting in parenthesis because missing from estimate. Not added if direct estimate was 0% because it would not change the estimate. See S1 Appendix for details on this table.

Multiplication of total number of carcasses evaluated by percentage/100 provides the number of carcasses with cited or potential lead poisoning.

-- = No data

See S1 Appendix for references.
