# Supplementary material for: Method to assess the potential magnitude of terrestrial European avian population reductions from ingestion of lead ammunition
Source: PLoS One. 2022 Aug 29;17(8):e0273572. doi: 10.1371/journal.pone.0273572 (PMC9423653; doi:10.1371/journal.pone.0273572)
Supplement: S2 Table — Ultimate estimates are based on liver lead concentrations and are assumed to be an upper bound of possible deaths. (DOCX) [file pone.0273572.s002.docx]

**S2 Table. Collected dead raptors estimated to have directly (cited as cause) or possibly ultimately (exceedance of sublethal lead concentrations) died of lead poisoning** Ultimate estimates (lethal plus sublethal deaths) are based on liver lead concentrations and are assumed to be an upper bound of possible deaths. This table presents data plotted in Fig. 2 and summarized as percentages by country in Table 2. Bolded studies used telemetry.

| **Country** | **Common Name** | **Species** | **No. Carcasses Evaluated for Cause of Death** | **No. Carcasses Evaluated that had Reported Tissue Concentrations** | **% with Ingested Lead Shot Cited as cause of death** | **% that are Potentially Lethal and Sublethal Lead-caused Deaths based on Tissue^a^** | **Years Collected** | **Source** |
| --- | --- | --- | --- | --- | --- | --- | --- | --- |
| United Kingdom | Eurasian sparrowhawk | *Accipiter nisus* | 150 | 150 | 0.0 | 0.7 | 1980s to 1990s | Pain et al. 1995 |
| United Kingdom | peregrine falcon | *Falco peregrinus* | 26 | 26 | 0.0 | 15.4 | 1980s to 1990s | Pain et al. 1995 |
| United Kingdom | common buzzard | *Buteo buteo* | 106 | 56 | 0.0 | 5.4 | 1990-1994 | Kenward et al. 2000, Pain et al. 1995 |
| United Kingdom | red kite | *Milvus milvus* | 162 | 44 | 4.1 | 15.9 | 1989-2007, 1995-2003 | **Molenaar et al. 2017,^b^**  Pain et al. 2007 |
| United Kingdom | Eurasian sparrowhawk | *Accipiter nisus* | 87 | 87 | 0.0 | 1.1 | 2010-2012 | Walker et al. 2014 |
| United Kingdom | red kite | *Milvus milvus* | 38 | 38 | 0.0 | 0.0 | 2010-2012 | Walker et al. 2014 |
| United Kingdom | common buzzard | *Buteo buteo* | 60 | 60 | 0.0 | 5.0 | 2008-2012 | Walker et al. 2014 |
| United Kingdom | common buzzard | *Buteo buteo* | 0 | 187 | -- | 8.0 | 2007-2018 | Taggart et al. 2020 |
| United Kingdom (Scotland) | red kite | *Milvus milvus* | 123 | 0 | 0.0 | -- | 1989-2014 | **Sansom et al. 2016** |
| Denmark (pre-ban) | common buzzard | *Buteo buteo* | 30 | 30 | 0.0 | 13.3 | 1976-1977 | Clausen and Wolstrup 1979 |
| Denmark (pre-ban) | 7 Eurasian sparrowhawks, 1 peregrine falcon | *Accipiter nisus, Falco peregrinus* | 8 | 8 | 0.0 | 0.0 | 1976-1977 | Clausen and Wolstrup 1979 |
| Netherlands (pre-ban) | common buzzard | *Buteo buteo* | 0 | 35 | -- | 11.4 | 1991 | Hontelez et al. 1992 |
| Netherlands (pre-ban) | common buzzard | *Buteo buteo* | 137 | 80 | 0.0 | 13.8 | 1992 | Jager et al. 1996 |
| Spain (Canary Islands) | Eurasian sparrowhawk | *Accipiter nisus* | 92 | 0 | 0.0 | -- | 1998-2007 | Rodriguez et al. 2010 |
| Spain (Canary Islands) | common buzzard | *Buteo buteo* | 65 | 0 | 0.0 | -- | 1998-2007 | Rodriguez et al. 2010, Palacios 2004 |
| Spain (Canary Islands) | peregrine falcon | *Falco peregrinus* | 20 | 0 | 0.0 | -- | 1998-2007 | Rodriguez et al. 2010 |
| Spain (Catalonia) | griffon vulture | *Gyps fulvus* | 47 | 0 | 2.1 | -- | 1995-2007 | Molina-López et al. 2011^b^ |
| Spain (Catalonia) | common buzzard | *Buteo buteo* | 905 | 0 | 0.0 | -- | 1995-2007 | Molina-López et al. 2011^b^ |
| **S2 Table. Continued.** | | | | | | | | |
| **Country** | **Common Name** | **Species** | **Carcasses (n, direct)** | **Carcasses (n, ultimate)** | **Reported  (direct %)** | **Maximum  (ultimate %)^a^** | **Years Collected** | **Source** |
| Spain (Catalonia) | Eurasian sparrowhawk | *Accipiter nisus* | 449 | 0 | 0.0 | -- | 1995-2007 | Molina-López et al. 2011^b^ |
| Spain (Catalonia) | northern goshawk | *Accipiter gentilis* | 212 | 0 | 0.0 | -- | 1995-2007 | Molina-López et al. 2011^b^ |
| Spain (Catalonia) | peregrine falcon | *Falco peregrinus* | 95 | 0 | 0.0 | -- | 1995-2007 | Molina-López et al. 2011^b^ |
| Spain (Catalonia) | 6 red kites, 2 bearded vultures, 13 hen harriers, 7 golden eagles | *Milvus milvus, Gypaetus barbatus, Circus cyaneus, Aquila chrysaetos* | 28 | 0 | 0.0 | -- | 1995-2007 | Molina-López et al. 2011^b^ |
| Spain | Egyptian vulture | *Neophron percnopterus* | 22 | 169 | 0.0 | 6.5 | 1998-2007 | **Donazar et al. 2002,**^c^ Gangoso et al. 2009^c^ |
| Spain | griffon vulture | *Gyps fulvus* | 58 | 0 | 0.0 | -- | 2014-2018 | Arrondo et al. 2020 |
| Spain | common buzzard | *Buteo buteo* | 7 | 7 | 0.0 | 0.0 | 1994 | Garcia-Fernandez et al. 1997 |
| Portugal | common buzzard | *Buteo buteo* | 0 | 56 | -- | 0.0 | 2007-2012 | Carneiro et al. 2014 |
| France/Spain (Pyrenees) | bearded vulture | *Gypaetus barbatus* | 20 | 20 | 5.0 | 10.0 | 2008 | Hernández and Margalida 2009b |
| France | red kite | *Milvus milvus* | 62 | 0 | 1.6 | -- | 1992-2002 | Berny and Gaillet 2008 |
| France (Pyrenees) | bearded vulture | *Gypaetus barbatus* | 8 | 8 | 0.0 | 0.0 | 2005-2012 | Berny et al. 2015 |
| France (Pyrenees) | griffon vulture | *Gyps fulvus* | 119 | 119 | 2.5 | 6.7 | 2005-2012 | Berny et al. 2015 |
| France (Pyrenees) | Red Kite | *Milvus milvus* | 34 | 34 | 11.8 | 17.6 | 2005-2012 | Berny et al. 2015 |
| France | Common buzzard | *Buteo buteo* | 0 | 90 | -- | 5.6 | 1998-1990 | Pain and Amiard-Triquet 1993 |
| France | Eurasian Sparrowhawk | *Accipiter nisus* | 0 | 32 | -- | 6.3 | 1998-1990 | Pain and Amiard-Triquet 1993 |
|  |  |  |  |  |  |  |  |  |
| **S2 Table. Continued.** | | | | | | | | |
| **Country** | **Common Name** | **Species** | **Carcasses (n, direct)** | **Carcasses (n, ultimate)** | **Reported  (direct %)** | **Maximum  (ultimate %)^a^** | **Years Collected** | **Source** |
| France | 2 peregrine falcons, 1 northern goshawk, 7 hen harriers | *Falco peregrinus, Accipiter gentilis, Circus cyaneus* | 0 | 8 | -- | 12.5 | 1998-1990 | Pain and Amiard-Triquet, 1993 |
| Italy (eastern) | 5 (+) common buzzards, 5 (+) Eurasian sparrowhawks, 1 peregrine falcon, 1 golden eagle | *Buteo buteo, Accipiter nisus, Falco peregrinus, Aquila chrysaetos* | 0 | 12 | -- | 0.0 | 1994-1995 | Alleva et al. 2006 |
| Italy (southern) | Common buzzard | *Buteo buteo* | 0 | 19 | -- | 10.5 | 1994-1996 | Zaccaroni et al. 2008 |
| Italy (southern) | 3 Eurasian sparrowhawks | *Accipiter nisus* | 0 | 3 | -- | 0.0 | 1994-1996 | Zaccaroni et al. 2008 |
| Austria/Italy/Switzerland Alps/French Pyrenees | bearded vulture | *Gypaetus barbatus* | 29 | 15 | 3.5 | 6.7 | 2005-2019 | Bassi et al. 2021 |
| Austria/Italy/Switzerland Alps/French Pyrenees | griffon vulture | *Gyps fulvus* | 112 | 62 | 10.7 | 19.4 | 2005-2019 | Bassi et al. 2021 |
| Austria/Italy/Switzerland Alps/French Pyrenees | golden eagle | *Aquila chrysaetos* | 92 | 52 | 20.7 | 36.5 | 2005-2019 | Bassi et al. 2021 |
| Austria/Italy/Switzerland Alps/French Pyrenees | cinereous vulture | *Aegypius monachus* | 19 | 12 | 0.0 | 0 | 2005-2019 | Bassi et al. 2021 |
| Italy/France's Alps & other Mountains | common buzzard | *Buteo buteo* | 18 | 18 | 0.0 | 22.2 | 1998-1999 | Battaglia et al. 2005 |
| Austrian, Swiss, German Alps | golden eagle | *Aquila chrysaetos* | 7 | 7 | 28.6 | 28.6 | 2000-2001 | Kenntner et al. 2007 |
| **S2 Table. Continued.** | | | | | | | | |
| **Country** | **Common Name** | **Species** | **Carcasses (n, direct)** | **Carcasses (n, ultimate)** | **Reported  (direct %)** | **Maximum  (ultimate %)^a^** | **Years Collected** | **Source** |
| Switzerland | golden eagle | *Aquila chrysaetos* | 67 | 55 | 9.0 | 9.1 | 2006-2017 | Ganz et al 2018, Jenni et al. 2015,  Madry et al. 2015 |
| Switzerland | red kite | *Milvus milvus* | 34 | 34 | 0.0 | 0 | 2009-2017 | Ganz et al. 2018 |
| Switzerland | bearded vulture | *Gypaetus barbatus* | 5 | 5 | 0.0 | 40 | 2006-2017 | Ganz et al. 2018 |
| Germany | northern goshawk | *Accipiter gentilis* | 61 | 61 | 1.6 | 4.9 | 1995-2001 | Kenntner et al. 2003 |
| Poland | common buzzard | *Buteo buteo* | 0 | 34 | -- | 11.8 | 2010-2014 | Kitowski et al. 2017 |
| Poland | Eurasian sparrowhawk, northern goshawk | *Accipiter nisus, Accipiter gentilis* | 0 | 19 | -- | 0.0 | 2009-2012 | Kitowski et al. 2016 |
| Greece (Crete) | 189 common buzzards, 123 griffon vultures, 9 golden eagles, 8 peregrine falcons, 7 bearded vultures | *Buteo buteo, Gyps fulvus, Aquila chrysaetos, Falco peregrinus, Gypaetus barbatus* | 336 | 14 | 0.0 | 0 | 1990-2002 | Xirouchakis 2004 |
| Sweden | golden eagle | *Aquila chrysaetos* | 111 | 111 | 7.2 | 11.7 | 2003-2011 | Ecke et al. 2017 |
| Sweden | northern goshawk | *Accipiter gentilis* | 67 | -- | 0.0 | -- | 1980-1985 | **Kenward et al. 1993** |
| Norway | golden eagle | *Aquila chrysaetos* | 0 | 116 | -- | 14.7 | 1973-2014 | Madslien et al. 2015 |
| Norway | gyrfalcon | *Falco rusticolus* | 0 | 37 | -- | 2.7 | 1973-2014 | Madslien et al. 2015 |
| **Europe** | **All birds** | **All birds** | **4128** | **2030** |  |  |  | |

^a^Subclinical or greater concentrations in liver were counted to estimate possible percentage of deaths ultimately caused by lead ammunition ingestion.

^b^Study included nestlings or orphans, which were excluded in this table because nestling survival is already incorporated into the fecundity multiplier.

^c^Study sampled blood which was converted to liver concentrations for ultimate death percentage using methods described in S1 Appendix.

Multiplication of total number of carcasses evaluated by percentage/100 provides the number of carcasses with cited or potential lead poisoning.

-- = No data

See S1 Appendix for references.
