# Supplementary material for: Method to assess the potential magnitude of terrestrial European avian population reductions from ingestion of lead ammunition
Source: PLoS One. 2022 Aug 29;17(8):e0273572. doi: 10.1371/journal.pone.0273572 (PMC9423653; doi:10.1371/journal.pone.0273572)
Supplement: S3 Table — Ultimate estimates are based on liver lead concentrations and are assumed to be an upper bound of possible deaths. (DOCX) [file pone.0273572.s003.docx]

**S3 Table. Percent of carcasses of grey partridges, common buzzards, red kites, and bearded vultures that died directly or ultimately of lead ammunition ingestion by country and across Europe.**

| **Study Country Group** | **Carcass Sample Sizes of all Causes of Death and Breeding Pair Abundance Used to Weight Lead Percentage for Europe** | | | |  |
| --- | --- | --- | --- | --- | --- |
|  | **No. Carcasses for Direct %^a^** | **No. Carcasses for Ultimate %** | **Breeding Pairs in all European Countries^b^** | **Direct %** | **Possible Ultimate %** |
| **Grey Partridge** | | | | | |
| United Kingdom | 959 (+208) | 446 | 43,190 | 1.54 | 4.48 |
| France | 503 | 0 | 935,089 | 0.00 | *0.00* |
| Denmark | 62 (+21) | 62 | 24,217 | 1.20 | 1.61 |
| Spain | 0 | 0 | 4,000 | *0.00* | *0.00* |
| Germany/Switzerland/Hungary | 85 | 0 | 6,800 | 0.00 | *0.00* |
| Italy | 31 | 2 | 497,576 | 0.00 | 0.00 |
| Norway | 0 | 0 | 20,000 | *0.00* | 0.00 |
| **Total Live Breeding Pairs of Common Buzzards in Europe** | | | **1,530,872** |  |  |
| **Weighted mean % of deaths by lead shot ingestion in Europe^b^** | | | | **0.06** | **0.15** |
| **Common Buzzard** | | | | | |
| United Kingdom | 166 | 303 | 69,500 | 0.00 | 6.93 |
| Denmark/Netherlands | 167 | 145 | 24,795 | 0.00 | 13.10 |
| Spain/Portugal | 977 | 63 | 40,138 | 0.00 | *6.24* |
| France | 0 | 90 | 131,000 | *0.00* | 5.56 |
| Italy/Austria/Switzerland | 18 | 49 | 50,500 | 0.00 | 12.24 |
| Germany/Poland | 0 | 34 | 313,560 | *0.00* | 11.76 |
| Greece | 189 | 0 | 6,000 | 0.00 | *0.00* |
| Norway/Sweden (use Germany/Poland) | 0 | 0 | 36,600 | *0.00* | *11.76* |
| **Total Live Breeding Pairs of Common Buzzards in Europe** | | | **672,093** |  |  |
| **Weighted mean % of deaths by lead ammunition ingestion in Europe^b^** | | | | **0.00** | **9.71** |
| **Red Kite** | | | | | |
| United Kingdom, Denmark/Netherlands | 323 | 82 | 504 | 1.86 | 8.54 |
| Spain/Portugal | 6 | 0 | 4,055 | 0.00 | *0.00* |
| France | 96 | 34 | 2,743 | 5.21 | 17.65 |
| Italy/Austria/Switzerland/Germany/Poland | 34 | 34 | 17,582 | 0.00 | 0.00 |
| **Total Live Breeding Pairs of Common Buzzards in Europe** | | | **24,884** |  |  |
| **Weighted mean % of deaths by ammunition ingestion in Europe^b^** | | | | **0.61** | **2.12** |
| **Bearded Vulture** | | | | | |
| Spain/France | 28 | 28 | 164 | 3.571 | 7.14 |
| Italy/Austria/Switzerland | 34 | 20 | 7 | 2.941 | 15.00 |
| Greece | 7 | 1 | 7 | 0.00 | 0.00 |
| **Total Live Breeding Pairs of Common Buzzards in Europe** | | | **177** |  |  |
| **Weighted mean % of deaths by lead ammunition ingestion in Europe^b^** | | | | **3.42** | **7.17** |

^a^ Added birds dying from hunting in parenthesis because missing from estimate.

Multiplication of total number of carcasses evaluated by percentage/100 provides the number of carcasses with cited or potential lead poisoning.

^b^Population estimate is used for weighting and is sum of breeding pairs in study country of susceptible species, plus all other EU27 countries (including UK) plus Switzerland and Norway, that might have similar exposure to the study country, as defined in Table D of S1 appendix.
