# Supplementary material for: Method to assess the potential magnitude of terrestrial European avian population reductions from ingestion of lead ammunition
Source: PLoS One. 2022 Aug 29;17(8):e0273572. doi: 10.1371/journal.pone.0273572 (PMC9423653; doi:10.1371/journal.pone.0273572)
Supplement: S1 Appendix — (DOCX) [file pone.0273572.s004.docx]

**S1 Appendix: Methods for Compiling Data in the Literature**

This appendix provides more details on data compilation in the tables and figures in the study, and associated assumptions. The purpose of the paper is to demonstrate a method to estimate lead ammunition ingestion effects on terrestrial bird populations and not to provide a definitive accurate estimate of bird losses in Europe. Data gaps prevent an accurate estimate for all countries and species, and we made some assumptions described below to fill in the gaps. We place these details in the appendix to illustrate how the method can still be used to assist decision-making when data are missing. Other assumptions or approaches that we used can fill the data gaps, as long as the approach uses the basic two steps discussed in the main paper. As more necropsy data and lead tissue concentrations in livers become available across Europe, the method can be used to develop improved Europe-wide estimates of lead-ammunition ingestion effects.

**Additional Information on Estimates in Figs. 1 and 2 in Main Paper (Data in S1 and S2 Tables)**

The literature search included an extensive review of the peer-reviewed literature through 2020 in journals, book chapters, and necropsy and tissue concentration reports in grey literature, as indicated in S1 and S2 Tables (full references of citations in those tables are in this appendix). Monclús et al. (2020) also reviewed raptor literature, and we checked to ensure we had evaluated applicability of all the articles they cited and added some, if missing. Studies selected had to include necropsy or cause of death information needed to assess direct mortality, and tissue concentrations in liver (or lead shot in gizzard for gallinaceous birds) to assess mortality ultimately due to lead ingestion. Some key species or countries did not have data on lead concentrations in livers.

To obtain the estimates of percentage of deaths that possibly ultimately died from lead ingestion for a few key studies that reported tissue lead concentrations other than liver concentrations in S1 and S2 Tables, we converted the percentages based on that tissue data to estimated liver-based percentages using ratios, as described below. Table A provides the thresholds of blood, liver, and bone considered subclinical, toxic, or expected to cause mortality that were used to identify critical thresholds for tissues different than liver (Franson 1996, Franson and Pain 2011, Pain et al. 2019a).

For the gallinaceous birds, we could not find liver lead concentrations for red grouse (*Lagopus lagopus*). Thomas et al. (2009) provided percentages exposed to lead shot for red grouse based on toxic bone concentrations (> 20 ppm), which we converted to liver-based percentages by multiplying their percentage by 0.65, calculated from the ratio of toxic (clinical) bone percentages with liver percentages exceeding toxic (clinical) thresholds from data provided in Pain et al. (2007). Also, Reitan (2013) reported muscle concentrations for lead in willow grouse that were very low, which we converted to liver-based concentrations using general ratios of bird liver and muscle data reported in that study (liver is 3 times that of muscle), and the concentrations were still very low and highly unlikely to exceed any critical lead thresholds.

| **Table A. Tissue Concentration thresholds (ppm) for effects of lead on terrestrial birds** | | | | |
| --- | --- | --- | --- | --- |
| **Tissue** | **Effect** | **unit^a^** | **Galliformes^b^** | **Falconiformes/Accipitriformes^c^** |
| Blood | Subclinical | ww | 0.6-<3^d^ | 0.2 - <0.5 |
|  | Toxic | ww | 3-10 | 0.5 - 1.0 |
|  | Severe Clinical | ww | >10 | >1.0 |
| Liver | Subclinical | ww | 2-<6 | 2 - <6 |
|  |  | dw | 6 - <18.6 | 6 - <18.6 |
|  | Toxic | ww | 6-15 | 6 - 10 |
|  |  | dw | 18.6-44 | 18.6 - 31 |
|  | Mortality | ww | >15 | > 10 |
|  |  | dw | >44 | > 31 |
| Bone | Subclinical | dw | 10-<15 | 10-20 |
|  | Toxic | dw | 15-20 | >20 |
|  | Severe Clinical | dw | >20 | -- |
| ^a^ww = wet weight, units in which studies were reported (except bone), dw = dry weight, dw conversion is C_dry_ = C_wet_ 100/(100-%moisture); 1 ppm ww = 3.1 ppm dw for liver based on mallard (Scanlon 1982, Franson and Pain 2011).  ^b^Franson (1996), Vallverdú-Coll et al. (2015, 2016), modified slightly to remove overlapping ranges  ^c^Franson and Pain (2011) and Pain et al. (2019a), where toxic is clinical poisoning and severe clinical poisoning is almost certain to cause death. | | | | |

The data in S1 and S2 Tables represent birds found dead. However, for gallinaceous birds, we converted some hunter-shot bird data to represent found dead birds, when no other data were available for a country (Spain) or species (red grouse) for direct or ultimate deaths. Three red-legged partridge (*Alectora rufa*) studies (Soler-Rodriguez et al. 2004, Ferrandis et al. 2008, Romero et al. 2020) and the two grouse studies were studies which represented the living populations (i.e., live birds sampled by hunting), not dead populations. Therefore, we converted the possible ultimate percentages (includes indirect deaths) from living to dead populations using the overall ratio of the total dead (1.8) and live population (4) percentages for Europe, obtained from data in Table B total gizzard percentages, by multiplying by 0.45 (= 1.8%/4.0%). Table B lists percentages of (1) gallinaceous birds with at least one lead shot in gizzard or (2) tissue concentrations exceeding toxic thresholds. Unlike S1 Table, this table does not provide reported dead bird percentages based on a lead shot poisoning diagnosis by a pathologist, nor percentages based on sublethal concentrations in liver (only concentrations exceeding toxic, clinical thresholds) and was used only for conversions of percentages from living to dead populations using data readily available in the literature.

| **Table B. Percent of adult and juvenile gallinaceous birds in Europe exposed to at least one lead shot in gizzard/crop or with toxic concentrations of lead in tissue.** | | | | | | | | | | | |
| --- | --- | --- | --- | --- | --- | --- | --- | --- | --- | --- | --- |
| **Species^a^** | **Country** | **Exposure Based on Gizzards with Shot** | | | **Exposure Based on Liver Tissue^b^** | | | **Collection Method** | **Years of collection** | **Lead shot density (no./ha)** | **Source** |
|  |  | **Number with Shot** | **Number birds examined** | **%** | **Toxic Lead** | **Number birds examined** | **%** |  |  |  |  |
| **Live Birds Sampled by Shooting or Sacrifice** | | | | | | | | | | | |
| Red-legged Partridge | Spain | 3 | 76 | 3.9 | 2 | 64 | 3.1 | Hunter shot | 2004, 2006 | 73,600 | Ferrandis et al. 2008 |
| Red-legged Partridge | Spain | 1 | 7 | 14 | 1 | 7 | 14 | Hunter shot | 2000 | NA | Soler-Rodriguez et al. 2004 |
| Common Pheasant | UK | 13 | 437 | 3.0 | -- | -- | -- | Hunter shot | 1996, 1997 1999-2000, 2001-2002 | NA | Butler et al. 2005 |
| Red-legged Partridge | UK | 2 | 144 | 1.4 | -- | -- | -- | Hunter shot | 2001 - 2002 | NA | Butler 2005 |
| Common Pheasant | Hungary | 45 | 923 | 4.9 | 53 | 935 | 5.7 | Hunter shot | 1989-1990s | 6,218 | Ákoshegyi 2000 |
| Willow ptarmigan | Norway | -- | -- | -- | 0 | 18 | 0 | Hunter shot | 2009 - 2011 | NA | Reitan 2013^c^ |
| Red-legged Partridge | Spain | 9 | 219 | 4.1 | 5 | 219 | 2.3 | Hunter shot, some found dead | 2016-2018 | NA | Romero et al. 2020 |
| Barbary Partridge | Spain | 1 | 13 | 0 | 2 | 13 | 15 | Hunter shot, some found dead | 2016-2018 | NA | Romero et al. 2020 |
| Common Quail | Spain | 0 | 31 | 0 | 1 | 31 | 3.2 | Hunter shot, some found dead | 2016-2018 | NA | Romero et al. 2020 |
| **All Birds** | **Europe** | **74** | **1850** | **4.0** | **64** | **1287** | **5.0** | **Various** | **1989 - 2018** | **NA** |  |
| **Dead Birds Found** | | | | | | | | | | | |
| Grey Partridge | UK | 20 | 446 | 4.5 | -- | -- | -- | Found dead | 1963 - 1992 | NA | Potts 2005 |
| Red-legged Partridge | UK | 1 | 503 | 0.2 | -- | -- | -- | Found dead | 1963 - 1992 | NA | Butler 2005 |
| Common Pheasant | Denmark | 0 | 199 | 0 | -- | -- | -- | Found dead or ill, a few were hunter-shot | 1971 - 1979 | NA | Clausen and Wolstrup 1979 |
| Grey Partridge | Denmark | 1 | 62 | 1.6 | -- | -- | -- | Found dead or ill, a few were hunter-shot | 1971 - 1979 | NA | Clausen and Wolstrup 1979 |
| Common Pheasant | Hungary | 0 | 24 | 0 | -- | -- | -- | Found dead | 1989-1990s | 6,218 | Ákoshegyi 2000 |
| **All Birds** | **Europe** | **22** | **1234** | **1.8** | **--** | **--** | **--** | **Various** | **1963 – 1990s** | **NA** |  |
| ^a^ Scientific names of common species are in S1 Table.  ^b^Uses" toxic" tissue thresholds in Table A.  ^c^Based on measured muscle lead in hunter-shot birds, which if tripled to approximate liver concentrations (Table 8 data in Reitan 2013) is still very low and not toxic. Collected during upland lead shot ban. | | | | | | | | | | | |

For raptors, we included some data based on blood from living birds. For the Egyptian vulture (*Neophron percnopterus)* studies (Donazar et al. 2002, Gangoso et al. 2009) in the Canary Islands of Spain (where lead shot embedded in small game occurs), lead concentrations were reported only in blood of the living population. In S2 Table, we included these two studies, assuming the exceedances of sublethal blood concentrations could represent exceedance of sublethal liver concentrations in dead birds (see comparison in Table C). If these two studies were excluded, no information on vultures that includes indirect losses from lead ammunition ingestion would have been included in our analysis for Spain. Although lead concentrations in blood from soil ingestion and bullet ingestion by vultures have been frequently studied in Spain (Espin et al. 2014, Mateo-Tomas et al. 2016, Arrondo et al. 2020; Table C), data on livers of collected dead birds is lacking for vultures in that country. The percentage exceeding toxic thresholds is similar for living birds, based on blood and dead birds, based on liver when summed across studies (Table C); therefore, we simply assumed the sublethal blood percentages for the Egyptian vulture represent sublethal liver percentages. We did not include other studies of blood lead in griffon vultures (*Gyps fulvus*) in Spain because those studies indicated exposure is not from lead ammunition, and a high probability exists that the exposure in Spain for this species is from lead in the soil with some bullet ingestion in big-game hunting areas (Arrondo et al. 2020). Regurgitated raptor pellet data were available (Table C) but are less reliable for evaluating toxicity than tissue data; thus, the pellet data were not used.

We also adjusted direct dead bird percentages in S1 Table so they included hunter-shot birds taken home in the bag when they were omitted in a study. Proportion of hunting deaths was not available for each study area. Although the percentage of living birds shot is variable year to year (e.g., Table 8 in Watson et al. 2007) and in different locations (Buenostado et al. 2009, Besnard et al. 2010), we assumed an average of 25% of grey partridge (*Perdix perdix*) deaths are from hunting (Meyer et al. 2016). This percentage is an estimate for a European continental mixture of released and wild dead birds from grey partridge populations studied in De Leo et al. (2004), where we converted their continental hunting rate of 14.8% to its equivalent of 25% of total deaths based on the annual mortality proportion (0.59) in the partridge population model at carrying capacity. Released grey partridges were assumed to be shot at similar rates to wild birds, because number shot on wild-bird estates is generally similar to number shot on estates with released birds (Robertson and Dowell 1990). For red-legged partridge, we used 27% (based on trapped and radio-tracked bird deaths across a range of hunted and non-hunted habitats; Buenestado et al. 2009). Direct mortality was 0% for other species and therefore no adjustment was needed.

As an example of the hunting adjustment of percentage of direct deaths from lead shot ingestion for grey partridge, Potts (2005) reported 3.4% of 446 grey partridge carcasses (15) were diagnosed as dying of lead shot poisoning from 1963 to 1992. This estimate was converted to the direct percentage in S1 Table by adding 149 more carcasses that represent 25% (hunting deaths) of 595 carcasses (no. hunting carcasses = 0.25*[no. non-hunting carcasses + no. hunting carcasses] or more simply [1-0.25]*original % without hunted carcasses). Adding these carcasses changes the 3.4% to 2.5% (15/595) that died from all causes. This adjustment assumes death of hunted birds was not caused by lead shot ingestion, which is true for direct percentage estimates of lead poisoning but may not be true for indirect percentages in the ultimate estimate, which were unadjusted as discussed in the main paper.

Additionally, ultimate deaths for raptors in Table 2 had some exceptions to the subclinical thresholds in Table A. Pain et al. (2007) used a raptor liver threshold of > 6.6 ppm dw; Madslien et al. (2015) used a liver threshold > 3 ppm dw; and Jager et al. (1996) used a threshold > 20 mg in content (latter assumed equivalent to the reported background/sublethal concentration threshold of 5 mg/kg dw for same study area in a related study, Hontelez et al. 1992).

Birds clearly identified as poisoned by a non-ammunition source were not included (e.g., one gyrfalcon [*Falco rusticolus*] in Madslien et al. 2015). Hontelez et al. (1992) and Jager et al. (1996) did not specifically evaluate lead ammunition effects in the Netherlands, creating uncertainty in the estimates for these studies, particularly because soil concentrations of lead are high in the Netherlands and may be responsible for higher liver lead concentrations, rather than lead shot (Jager et al. 1996). However, the Hontelez et al. (1992) sublethal-based percentage for ultimate deaths was based on the 4 radiographed birds reported as having lead pellets and correspondingly, higher lead concentrations in liver than background. The Jager et al. (1996) estimate in S2 Table is less certain because they did not utilize radiographs to confirm, and our estimate may be biased high if it is not a result of lead ammunition ingestion. However, when the Jager et al. (1996) researchers examined the data of the Hontelez et al. (1992) study that was in the same study area as theirs, lead concentrations in liver were similar between the two studies, and likewise our estimates of sublethal liver lead concentrations exceedances were generally similar between these two studies, which supports the Jager et al. (1996) estimate as being reasonable. For other studies that did not investigate isotopes or use radiographs or regurgitated pellet analysis, the estimates may be biased high if not due to lead ammunition. Monclús et al. (2020) summarize many of the studies we used (103 publications) and identified if those studies evaluated the source of contamination using isotopes.

Because lead shot ingestion in partridges increased after 1963 (Potts 2005), we excluded studies or data collected before 1963 from the exposure estimates in S1 and S2 Tables to represent more recent past and current exposure without lead shot bans in uplands. Exposure in uplands is presently variable among countries not only due to variable hunting intensity and type, but also because some countries banned lead shot in upland areas in the 1990s (Netherlands in 1993, Denmark in 1996, Flemish region of Belgium in 2008, Valencia province in Spain in 2018; see Thomas and Guitart 2010 and Meyer et al. 2016) or for a period of time in the 2000s (Norway ban was from 2005 to early 2015; Arnemo et al. 2016). Mateo and Kanstrup (2019) summarize the status of lead shot bans in Europe. Data in S1 and S2 Tables were predominantly from pre-ban periods even for countries with upland bans (e.g., Denmark and Netherlands study and ¾ of a Norwegian study were pre-ban for raptors, although another Norwegian study was post-ban for willow grouse because no other data were available pre-ban). Additionally, studies that did not appear to attempt to diagnose birds for lead poisoning were not included to estimate direct percentages, although studies with very large necropsy programs were assumed to have professionals that could identify signs of lead poisoning.

| **Table C. Percent of diurnal raptors in Europe with toxic concentrations of lead in tissue or at least one lead shot or bullet fragment in regurgitated pellets.** | | | | | | |
| --- | --- | --- | --- | --- | --- | --- |
| **Species** | **Country** | **Exposure^a^** | | | **Years of collection** | **Source** |
|  |  | **Toxic Lead Level or Pellet contained Shot** | **Number Examined** | **Percent** |  |  |
| **Live Birds Trapped with Cannon Nets or Walk-in Traps and Blood Sampled** | | | | | | |
| Egyptian Vulture | Iberian Peninsula, Spain | 0 | 32 | 0% | 2002 - 2004 | Gangoso et al. 2009 |
| Egyptian Vulture | Canary Islands, Spain | 3 | 137 | 2% | 1999 - 2005 | Gangoso et al. 2009, Donazar et al. 2002 |
| Griffon Vulture | Spain | 2 | 23 | 9% | 2003 | Garcia-Fernandez et al. 2005 |
| Griffon Vulture | Spain (northeastern) | 28 | 691 | 4% | 2008-2012 | Mateo-Tomas et al. 2016^b^ |
| Griffon Vulture | Spain (eastern) | 3 | 66 | 5% | 2011 | Espin et al. 2014 |
| Griffon Vulture | Spain and Portugal | 1 | 121 | 1% | 2011 | Carneiro et al. 2015 |
| Griffon Vulture | Spain | 23 (most from soil) | 58 | 40% | 2014-2018 | Arrondo et al. 2020 |
| **Total of all Birds** | **Spain and Portugal** | **37** | **949** | **4%** | **1999-2004** |  |
| **Dead Birds Collected and Liver Sampled** | | | | | | |
| Common Buzzard | Austria/Italy/Switzerland/France | 2 | 18 | 11% | 1998 - 1999 | Battaglia et al. 2005 |
| Cinereous Vulture | Austria/Italy/Switzerland/France | 0 | 12 | 0% | 2005-2019 | Bassi et al. 2021 |
| Golden Eagle | Austria/Italy/Switzerland/France | 13 | 52 | 25% | 2005-2019 | Bassi et al. 2021 |
| Griffon Vulture | Austria/Italy/Switzerland/France | 7 | 62 | 11.3% | 2005-2019 | Bassi et al. 2021 |
| Bearded Vulture | Austria/France/Italy | 1 | 15 | 6.7% | 2005-2019 | Bassi et al. 2021 |
| Griffon Vulture | France (Pyrenees) | 8 | 119 | 6.7 | 2005-2012 | Berny et al. 2015 |
| Bearded Vulture | France (Pyrenees) | 0 | 8 | 0 | 2005-2012 | Berny et al. 2015 |
| Egyptian Vulture | France (Pyrenees) | 0 | 9 | 0 | 2005-2012 | Berny et al. 2015 |
| Red Kite | France (Pyrenees) | 6 | 34 | 17.6 | 2005-2012 | Berny et al. 2015 |
| Northern Goshawk | Germany | 3 | 61 | 5% | 1995 - 2001 | Kenntner et al. 2003 |
| Common Buzzard | Poland | 0 | 34 | 0% | 2010-2014 | Kitowski et al. 2017 |
| Common Buzzard | Denmark (pre-ban)^d^ | 1 | 30 | 3% | 1976-1979 | Clausen and Wolstrup 1979 |
| Common Buzzard | Netherlands (pre-ban) | 0 | 35 | 0% | 1991 | Hontelez et al. 1992 |
| Bearded Vulture | Spain | 1 | 30 | 3% | 2008 | Hernández and Margalida 2009 |
| Common Buzzard | Spain | 1 | 44 | 2% | before 2006 | Perez-Lopez et al. 2008 |
| Common Buzzard | Portugal | 0 | 56 | 0% | 2007-2012 | Carneiro et al. 2014 |
| Common Buzzard | France | 3 | 90 | 3% | Winter 1988-1990 | Pain and Amiard-Triquet 1993 |
| Peregrine Falcon | France | 0 | 2 | 0% | Winter 1988-1990 | Pain and Amiard-Triquet 1993 |
| Eurasian Sparrowhawk | France | 2 | 32 | 6% | Winter 1988-1990 | Pain and Amiard-Triquet 1993 |
| Red Kite | UK | 6 | 44 | 14% | 1995 - 2003 | Pain et al. 2007 |
| Common Buzzard | UK | 2 | 56 | 7% | 1980s – 1990s | Pain et al. 1995 |
|  |  |  |  |  |  |  |
| **Table C. Continued.** | | | | | | |
| **Species** | **Country** | **Exposure^a^** | | | **Years of collection** | **Source** |
|  |  | **Toxic Lead Level or Pellet contained Shot** | **Number Examined** | **Percent** |  |  |
| **Dead Birds Collected and Liver Sampled** | | | | | | |
| Peregrine Falcon | UK | 2 | 26 | 15% | 1980s – 1990s | Pain et al. 1995 |
| Eurasian Sparrowhawk | UK | 1 | 150 | 1% | 1980s – 1990s | Pain et al. 1995 |
| Red Kite | UK | 0 | 6 | 0% | 1980s – 1990s | Pain et al. 1995 |
| Northern Goshawk | UK | 0 | 6 | 0% | 1980s – 1990s | Pain et al. 1995 |
| Red Kite | UK | 0 | 38 | 0% | 2010-2012 | Walker et al. 2014 |
| Eurasian Sparrowhawk | UK | 0 | 87 | 0% | 2010-2012 | Walker et al. 2014 |
| Common Buzzard | UK | 0 | 60 | 0% | 2010-2012 | Walker et al. 2014 |
| Golden Eagle | European Alps (Switzerland, Austria, Germany) | 2 | 7 | 29% | 2000-2001 | Kenntner et al. 2007 |
| Golden Eagle | Swiss Alps | 2 | 26 | 8% | 2006-2017 | Ganz et al. 2018, Jenni et al. 2015,  Madry et al. 2015 |
| Red Kite | Swiss Alps | 0 | 34 | 0% | 2009-2017 | Ganz et al. 2018 |
| Bearded Vulture | Swiss Alps | 1 | 5 | 20% | 2009-2017 | Ganz et al. 2018 |
| Golden Eagle | Norway | 8 | 116 | 7% | 1973-2014 (lead ban 2005-2014) | Madslien et al. 2015 |
| Gyrfalcon | Norway | 1 | 37 | 3% | 1973-2014 (lead ban 2005-2014) | Madslien et al. 2015 |
| **All Birds** | **Europe** | **50** | **1213** | **4%** | **1976-2017** |  |
| **Regurgitated Pellets from Live Birds Collected and Sampled for Lead Shot^c^** | | | | | | |
| Egyptian Vulture | Iberian Peninsula (Spain) | 0 | 327 | 0% | 1993-2003 | Gangoso et al. 2009 |
| Egyptian Vulture | Canary Islands, Spain | 13 | 424 | 3% | 1998 - 2001 | Donazar et al. 2002 |
| Red Kite | UK | 25 | 264 | 9% | 1995 - 2003 | Pain et al. 2007 |
| Red Kite | Spain | 13 | 1233 | 1% | Unavailable in English | Garcia and Vinuela 1999  (as cited in Mateo 2009) |
| Red Kite | Spain | 21 | 962 | 2% | 1991 - 1996 | Mateo 2009 |
| Peregrine Falcon | Spain | 1 | 117 | 1% | 1997 - 2002 | Mateo et al. 2007 |
| Bonelli's eagle | Spain | 44 | 1535 | 3% | 2004-2015 | Gil Sanchez et al. 2018 |
| **All Birds** | **Europe** | **117** | **4862** | **2%** | **1993-2015** |  |
| **Captured live, killed and liver sampled** | | | | | | |
| Common Buzzard | Spain | 0 | 5 | 0% | 1993 | Garcia-Fernandez et al. 1995 |
| Eurasian Sparrowhawk | Spain | 0 | 3 | 0% | 1993 | Garcia-Fernandez et al. 1995 |
| **All Birds** | **Spain** | **0** | **8** | **0%** | **1993** |  |
| ^a^Uses toxic tissue or blood thresholds in Table A, except for blood in griffon vultures (see footnote c)  ^b^Does not include one high lead concentration sample that did not fall within the isotopic signature of ammunition.  ^c^Authors used threshold of 1 ppm in blood as threshold for toxic level, rather than 0.5 (used for all other studies), which may be appropriate for griffon vultures (captured an apparently healthy bird with 3 ppm).  Kanstrup et al. (2019) collected 48 buzzards from 2013-2016 post-ban and thus is not included. No livers were > 6 mg/kg dw, except 1, which was likely not due to lead ingestion but rather to lab procedure. | | | | | | |

**Additional Information on Estimates in Tables 1 and 2 in Main Paper**

# Based on our review of the literature and discussions with the ammunition industry, we assigned breeding pairs in countries without data based on countries with data assumed to have similar lead ammunition ingestion exposure (Table D) to obtain a breeding-pair-weighted estimate of percentages of dead birds that died from lead ammunition ingestion in Tables 1 and 2 of the main paper. Often the decision was influenced by geographic proximity. The population of willow ptarmigan (same species as willow grouse and red grouse, which are different subspecies) in Europe contains between 1 and 2 million birds but has not been associated with lead ammunition ingestion except in the UK, where it occurs in heather; thus, we included its population size only where it might be susceptible or where lead concentration data were available (UK and Norway; also see Rodrigue et al. 2005).

**Table D. Country assignments.**

| **Countries with Data** | **Countries without Data Assigned to Country with Data** |
| --- | --- |
| **Gallinaceous Birds** |  |
| United Kingdom | Ireland |
| France | Luxembourg |
| Denmark | Belgium, Netherlands |
| Germany/Switzerland/Hungary | Austria, Bulgaria, Estonia, Latvia, Lithuania, Poland, Romania, Slovakia, Czech |
| Italy | Greece, Slovenia |
| Norway | Finland, Sweden |
| **Diurnal Raptors** |  |
| United Kingdom | Ireland |
| France | Luxembourg |
| Denmark/Netherlands | Belgium |
| Spain/Portugal | Azores, Madeira |
| Germany/Poland | Bulgaria, Estonia, Hungary, Latvia, Lithuania, Romania, Slovakia, Czech |
| Italy/Austria/Switzerland^a^ | Slovenia |
| Sweden/Norway | Finland |
| Greece | Cyprus |

^a^One study included in this group, Bassi et al. (2021), studied lead poisoning of raptors in this mountainous region but also included carcasses from the French Pyrenees, which could not be separated out.

Some groups or species were excluded from the final estimates in Tables 1 and 2 in the main paper. Game birds raised in captivity and released for shooting were not included in the population size estimates unless they survived to be counted as a breeder. One gallinaceous bird, the common quail (*Coturnix coturnix*), was not included even though susceptible (Romero et al. 2020), because it is highly migratory and its populations are exposed to lead shot ingestion in Africa, where they are most abundant and breeding (European Commission 2009); thus, they might not reflect European lead shot poisoning levels. Chukar (*Alectoris chukar*) are a sensitive species (Runia and Solem 2017) that has ingested lead shot in the United States (in Utah; Bingham et al. 2015), but we excluded them because of no reports of lead shot ingestion in that species in Europe. Other grouse species not in Table 1 (e.g., black grouse [*Tetrao tetrix*]) are also not included because of no reports of lead shot ingestion, and they occur in forested or other habitats less exposed to small game hunting pressure than agricultural areas. These species could be added in the future if records of lead shot ingestion become available. Alternatively, they could be added to the total susceptible number of birds by applying ingestion rates of more exposed species, as ECHA (2021) did, with the caveat of possible overestimation of effects on these species because their ingestion rates are likely very low but high rates would be applied to their numbers.

Diurnal raptor species with high lead ammunition ingestion rates, such as the white-tailed eagle (*Haliaeetus albicilla*) (Kenntner et al. 2001) and Spanish Imperial eagle (*Aquila adalberti*) (Mateo et al. 2001), were excluded from our analysis of terrestrial raptors because they can also feed on waterfowl and in wetlands or other aquatic habitats and do not represent terrestrial birds with a completely terrestrial diet. However, they can ingest lead ammunition embedded in prey while foraging in uplands. For example, studies on these raptor species indicate the lead ammunition source for terrestrial food sources generally is from lead bullets embedded in ungulates or their offal left behind by hunters (Kenntner et al. 2001, Krone et al. 2009, Nadjafzadeh et al. 2013, Madslien et al. 2015, Isomursu et al. 2018). In order to understand the terrestrial impact, separation of wetland impacts from terrestrial impacts for species feeding in both habitats would be needed but is beyond the scope of this paper. This subject that should be researched in the future.

S3 Table uses the same studies and data in S1 and S2 tables but reduces the studies to four individual species, the grey partridge, common buzzard, red kite, and bearded vulture.

**Effects of Lead Shot Ingestion in Raptors**

It is unknown if the source of lead ammunition is from bullets or lead shot, and the impact of lead shot alone is also unknown. We adjusted carcass death percentages to represent percentages due to lead shot ingestion (as opposed to bullet ingestion) for raptors by country using broad assumptions of main food sources available and proportion of game likely shot with shotguns versus rifles. Taggart et al. (2020) relied on statistics of shot game in the UK (from PACEC 2006) and known food sources of buzzards to report that lead ammunition-based concentrations in buzzard tissue in the UK were from lead shot ingestion rather than lead bullet ingestion. They also used lead isotopic signatures to differentiate lead ammunition from other sources but did not compare lead shot to lead bullet signatures (often difficult to separate), and thus relied on this other information. To approximate the impact of lead shot alone, we used that same method to adjust each country-wide estimate to remove carcasses with lead bullet ingestion, when such data were available. As an example of our calculations, we multiplied the direct and possible ultimate percentages of deaths from ingestion of lead ammunition for Sweden and Norway from the S2 Table by 0.33 (33% attributed to lead shot) to account for the dominance of big game hunting using bullets in Fennoscandia and because the studies in those countries stated large game is the main raptor food source. We derived the 0.33 as follows. Lecocq (1997) reported the number of terrestrial animals harvested of the 6 common game species in 8 European countries in 1995 (Table E), from which we derived the percentage of terrestrial game killed with lead shot (birds, rabbits, and hares) as opposed to bullets (other mammals). Game killed with lead shot averaged 25% in Sweden and Finland. This estimate is based on incomplete information because it omits many game species. To include all game species, we adjusted this percentage further by applying a ratio developed from UK data. The 1995 percentage estimate of lead-shot killed game for the UK (77%) was compared to a more comprehensive 2004 UK percentage estimate (99%) that included other hunted species (data available in PACEC 2006, reported in Table F; more recent PACEC UK estimates were not applied to make sure we match the older data to the older death percentage data in S1 and S2 Tables). The ratio of the two UK estimates (99/77) was 1.28; therefore, we used this ratio as a correction factor needed to include species that were missing in Lecocq estimates. The ratio increased the Fennoscandia estimates by 28%, producing an estimate of 33% of game shot with lead shot in Sweden and Finland. The 1.28 ratio was applied to percentages for each country from the Lecocq (1997) data in Table F, producing the estimates for 8 individual countries in Table G. Countries were then combined to match combinations in Table 2, and we averaged the percentages harvested with lead shot. For countries without easily-accessible animal harvest data (not in Table E) or that do not represent reported bullet use in our compiled lead ammunition studies (France), we relied on food source descriptions, as discussed below.

For Spain, which did not have hunting statistics readily available and few studies of raptor liver concentrations, we assumed 50% lead shot exposure because of the type of ammunition exposure to non-vultures and vultures present in mostly small- and big-game hunting areas, respectively. Arrondo et al. (2020) reported a low probability that lead in the blood of griffon vultures found throughout Spain was from ammunition; however, when it was from ammunition, it likely was from bullets from big game hunting. In contrast, some non-vultures such as common buzzards obtain more lead exposure in small-game than in big-game hunting areas in Spain (Perez-Lopez et al. 2008, Gil-Sanchez et al. 2018). We selected 50% lead shot exposure to represent this mix of sources. Table E also omits Poland and Greece, which were study countries in Table 2. The Polish studies in S2 Table indicated exposure was from lead shot ingestion. In contrast, lead shot exposure (32%) was a smaller percentage than bullet exposure in Germany (Table G). Combining the two countries in Table 2, we assumed 67% was from lead shot ingestion. The lead shot ingestion data for Greece were from Crete, where only small-game hunting exposure occurs; thus, Greece was assigned 100% lead shot exposure. Although Table F suggests all raptor exposure in France is from lead shot ingestion, we changed the percentage to 50% because wild boar hunting has increased substantially over time (Massei et al. 2014), and the studies we relied on for carcass percentages represent a mix of small-game hunting in non-mountainous areas and big-game hunting in more mountainous areas (Berny et al. 2015, Bassi et al. 2021). Our final applied estimates in Table G have uncertainty and can be refined as more information on bullet versus lead shot use by country becomes available.

**Table E. Annual kill of common terrestrial game species in eight European countries, 1995.**

| **Country** | **Roe deer *(Capreolus capreolus)*** | **Wild boar *(Sus scrota)*** | **Elk (Moose in US) *(Alces alces)*** | **Brown hare *(Lepus europaeus)*** | **Grey partridge *(Perdix perdix)*** | **Eurasian Woodcock *(Scolopax rusticola)*** |
| --- | --- | --- | --- | --- | --- | --- |
| Austria | 260,000 | 10,000 | 0 | **170,000** | **10,000** | **3,300** |
| Denmark | 87,000 | 0 | 0 | **167,000** | **97,000** | **34,000** |
| Finland | 5900 | 0 | 46,900 | **14,500** | **1,400** | **4,500** |
| France | 235,000 | 200,000 | 0 | **1,560,000** | **2,180,000** | **1,320,000** |
| Germany | 1,023,000 | 313,000 | 0 | **423,000** | **17,000** | **6,200** |
| Netherlands | 11,000 | 1,900 | 0 | **225,700** | **22,800** | **16,900** |
| Sweden | 382,000 | 1,600 | 94,240 | **100,900** | **6,000** | **36,700** |
| United  Kingdom | 200,000 | 0 | 0 | **396,000** | **187,500** | **100,000** |

From Lecocqu 1997. **Bolded** numbers are assumed to have been generally killed with shot from shotguns.

**Table F. Number of animals shot of game species in the UK in 2004.**

| **Species** | **Number shot** |
| --- | --- |
| Pheasant | **15,000,000** |
| Partridge | **26,000,000** |
| Grouse | **400,000** |
| Woodcock/snipe | **250,000** |
| Deer | 120,000 |
| Pigeon | **3,600,000** |
| Hare | **47,000** |
| Rabbit | **590,000** |
| Corvid | **380,000** |
| Mink | 1,000 |
| Fox | 120,000 |
| Stoat/Weasel | 9,000 |
| Squirrel | 170,000 |
| Feral Cat | 9,000 |
| Rat | 38,000 |
| Other | 8,000 |

From PACEC (2006).

**Bolded** numbers are assumed to have been generally killed with gunshot from shotguns.

**Table G. Percent of carcasses assumed to have died from lead shot ingestion of those that died from all lead ammunition sources.**

| **Country** | **Based on shot game %** | **Final after adjusting for food sources of study areas in S1 and S2 Tables** | |
| --- | --- | --- | --- |
| **Individual Country** | | **Combined Study Countries in Table 2** | |
| Austria | 52% | Italy/Austria/Switzerland | 50% |
| Denmark | 99% | Denmark/Netherlands | 99% |
| Netherlands | 100% |  |  |
| Finland | 36% | Norway/Sweden | 33% |
| Sweden | 30% |  |  |
| France | 100% | France | 50% |
| Germany | 32% | Germany/Poland | 67% |
| United Kingdom | 99% | United Kingdom | 99% |
|  |  | Greece | 100% |

Removal of the approximate lead bullet impacts using our assumptions reduced the raptor midpoint percentage of carcasses with lead poisoning by 2.2% (from 5.2% to 3%). The reduction in population size and other demographic parameters for lead shot alone are uncertain because the above assumptions were necessary but are presented below as preliminary estimates, with Table H showing the lead shot-caused direct and possible ultimate carcass percentage estimates. These were inputs to the population models. Table I provides the resultant estimates of the population model parameters and percent and total breeding pairs lost with lead shot alone. The estimates of bird losses from lead shot ingestion alone across Europe represent approximately half of the estimate when bullets are included.

**Table H. Percent of diurnal raptor carcasses that died directly or ultimately of lead ammunition ingestion by country and across Europe.**

| **Country (% attributed to lead shot)** | **Carcass Sample Sizes of all Causes of Death and Breeding Pair Abundance Used to Weight Lead Percentage for Europe** | | | | **Direct % of lead shot only** | **Possible Ultimate % of lead shot only** |
| --- | --- | --- | --- | --- | --- | --- |
|  | **Carcasses for Direct %** | **Carcasses for Ultimate %** | **Breeding Pairs in Study Country^a^** | **Breeding Pairs in all European Countries^b^** |  |  |
| United Kingdom (99% lead shot) | 752 | 648 | 107540 | 121,689 | 0.79 | 5.19 |
| Denmark/Netherlands (99% lead shot) | 175 | 153 | 23,845 | 38,783 | 0 | 12.29 |
| Spain/Portugal (50% lead shot) | 2000 | 232 | 107,578 | 107,578 | 0.03 | 2.37 |
| France (50% lead shot) | 243 | 311 | 177,419 | 178,817 | 1.85 | 3.86 |
| Italy/Austria/Switzerland (50% lead shot) | 383 | 294 | 63,782 | 73,744 | 5.22 | 7.99 |
| Germany/Poland (67% lead shot) | 61 | 114 | 253,688 | 469,810 | 1.10 | 4.70 |
| Greece (100% lead shot) | 336 | 14 | 12,144 | 12,293 | 0 | 0 |
| Norway/Sweden (33% lead shot) | 178 | 264 | 96,319 | 114,401 | 1.48 | 3.88 |
| **Total Live Breeding Pairs in Europe** | | | | **1,117,112** |  |  |
| **Weighted mean % of deaths by lead shot ingestion in Europe^b^** | | | | | **1.34** | **4.74** |
| ^b^ Population estimate in study countries shown of the most-susceptible species in 2012. Species included were red kite, common buzzard, Egyptian vulture (Canary Islands only), bearded vulture, griffon vulture, cinereus vulture, peregrine falcon, golden eagle, Bonelli's eagle, northern goshawk (*Accipiter gentilis*), Eurasian sparrowhawk (*Accipiter nisus)*, hen harrier (*Circus cyaneus)*, and gyrfalcon (*Falco rusticolus)*. This column represents study countries (not all countries) and thus was not used to weight carcass percentages.  ^b^ Population estimate is sum of breeding pairs in study country of the susceptible species, plus all other EU27 countries (including UK) plus Switzerland and Norway, that might have similar exposure to the study country, as defined in Table D of S1 Appendix. This column was used to weight death percentages before averaging because it includes all countries (note: weights for each country are country totals in this column divided by 1,117,112). | | | | | | |

| **Table I. Stochastic population modeling results for raptor lead shot ingestion alone with effects on reproduction.** | | | |
| --- | --- | --- | --- |
| **Output Parameter** | **Without Lead Shot** | **With Lead Shot** | **Change Due to Lead Shot** |
| **Terrestrial Diurnal Raptor Models (3.1% poisoned carcasses, 1.7% of birds with reproduction reduced by 75%)** | | | |
| **Common Buzzard/Red Kite Model Average (representing stable or decreasing small-bodied raptor populations in Europe)** | | | |
| Breeding pairs/10,000 km^2^ in local population | 1,656 | 1,638 | 18 |
| Probability of local quasi-extinction to < 350 breeding pairs/10,000 km^2^ in 50 years | 0.010 | 0.019 | 0.009 |
| **Red Kite Model (representing stable and decreasing small-bodied raptor populations in Europe)** | | | |
| Breeding pairs/10,000 km^2^ in increasing local population at final steady state | 1,695 | 1,681 | 14 |
| Probability of local quasi-extinction to < 350 breeding pairs/10,000 km^2^ in 50 years | 0.00 | 0.00 | 0.00 |
| **Red Kite Model (representing increasing small-bodied raptor populations in Europe)** | | | |
| Breeding pairs/10,000 km^2^ in increasing local population (average of first 50 years) | 1,695 | 1,642 | 53 |
| Probability of local quasi-extinction to < 350 breeding pairs/10,000 km^2^ in 50 years | 0.0022 | 0.0042 | 0.0020 |
| Maximum population growth rate | 1.065 | 1.057 | 0.008 |
| **Bearded Vulture Model (representing stable and decreasing large-bodied raptor populations in Europe)** | | | |
| Breeding pairs/60,000 km^2^ in increasing local population at final steady state | 192 | 189 | 3 |
| Probability of local quasi-extinction to < 900 birds/60,000 km^2^ in 50 years | 0.001 | 0.005 | 0.004 |
| **Bearded Vulture Model (representing increasing large-bodied raptor populations in Europe)** | | | |
| Breeding pairs/60,000 km^2^ in increasing local population (average of first 50 years) | 188 | 184 | 4 |
| Probability of local quasi-extinction to < 350 breeding pairs/60,000 km^2^ in 50 years | 0.015 | 0.031 | 0.016 |
| Maximum population growth rate | 1.078 | 1.076 | 0.002 |
| **Raptor Models Combined** |  |  |  |
| Estimated raptor breeding pairs "lost" from lead shot ingestion in Europe | **24,313 – 36,900 (2.1 – 3.3% decrease)^a^** | | |

^a^Range represents weighting by percent of raptor trends in each type (increasing, decreasing, stable) in first period of 2001 to 2012 (lower percent) to second period of 2013 to 2018 (upper percent).

**Reproduction Assumptions**

For gallinaceous birds, we assumed 3 lead shot reduced hatching success by 23% for all gallinaceous birds except pheasants (*Phasianus colchicus*). Hatching was reduced by 6% for pheasants. These estimates were derived as follows. In the laboratory, ingestion of 3 lead shot weighing a total of 318 mg reduced red-legged partridge hatching success by 23% (Vallverdú-Coll et al. 2016), whereas ingestion with 6 lead shot per week for 10 weeks weighing a total of 1500 mg each week reduced pheasant hatching success by that same amount (Gasparik et al. 2012). Ingestion of 1 lead shot was not adverse for partridge reproduction because it produced heavier chicks in the partridge study, a positive result for chick survival. The effect of two lead shot, falling between the two doses for a positive and negative result, was not measured in the red-legged partridge study but was assumed to have no effect.

Because too many lead shot in the gizzard (i.e., 4 or more) can lead to high probability of lead poisoning and death (Potts 2005, Kerr et al. 2010), we assumed ingestion of exactly 3 lead shot is required to reduce reproduction of gallinaceous birds, and a higher number would cause death before the bird could reproduce. Data in Butler et al. (2005) indicated that 0.32% of live pheasants before they were shot had exactly 3 pellets of lead shot in the gizzard during hunts on Great Britain shooting estates. Because pheasants may be less sensitive than other gallinaceous birds, we assumed the red-legged partridge result of 23% reduction in reproduction applies to 0.32% of gallinaceous bird populations except pheasants. The actual percentage exposed during the breeding season may be lower than this hunting-season percentage, but we used this value to demonstrate the method.

For pheasants, we also assumed that 0.32% of the population would ingest 3 lead shot (318 mg), which would reduce hatching success. However, the reduction in hatching success was estimated to be less than for partridges because of their higher tolerance, which we estimated by regressing the weekly lead dose in terms of shot weight (SHOTWT, in mg) against hatching success (HS, in %) using the pheasant data in Gasparik et al. (2012). The regression was HS = -0.0108 *SHOTWT+72.6. Using that regression, the 318 mg would reduce pheasant hatching success relative to control pheasants by 6.5%. The actual effect is likely much less because the dose of 1500 mg was per week, and lead shot likely was retained longer than 1 week in pheasants. For example, Kerr et al. (2010) reported lead shot pellet retention rates in bobwhite quail (*Colinus virginianus*) of 21% the first week, 7% the second week, and 0% the third week. However, Runia and Solem (2017) reported retention time varied; but on average, half of the lead shot pellets fed to pheasants were still retained after 21 days.

To calculate the percentage of the living raptors that are sublethally affected, we assumed the proportion of all bird carcasses that ultimately did not die from lead ammunition ingestion but had sublethal lead concentrations would be similar to the proportion of all living birds that had sublethal but survivable concentrations. The difference between direct (2.52%) and ultimate (7.90%) death percentages from lead ammunition for Europe (Table 2 in main paper) is the percentage of dead birds estimated to have sublethal effects that contributed to deaths attributed to other causes (5.38%). We assumed half (2.7%) ultimately died from the sublethal lead poisoning, weakening them before reproducing, and the other half (2.7%) could have reproduced if they had lived. Recalling that 92.1% of European raptor deaths are assumed to have not been associated with any lead ammunition ingestion (100% minus 7.9% possible ultimate deaths in Table 2), we assumed the sublethal percentage of the dead population of birds not dying from lead poisoning was 2.85% (= 100_*_(2.7/[92.1+2.7])), which represents the percentage of living birds with reproduction reduced by 75%. Using the same calculations, but restricted to percentages we approximately estimated for just lead shot, the sublethal percentage of the dead population of birds not proximally dying from lead was 1.7%.

**Vital Rate Multipliers for Raptor Population Models**

The survival terms, S_x_, and reproductive terms, referred to as fertility, F_x_, in the baseline pre-breeding stage matrix of the raptor models are provided in Meyer et al. (2016). Because mortality varies by age class and is a term in Equation 1 of the main paper, the survival multipliers used to decrease these terms to represent effects of lead shot ingestion varied by age class (x) for the representative species of raptors, as shown in Table J. The fertility multiplier, however, is constant across all age classes (Table J).

**Table J. Survival (S_x_) multipliers for age classes and fertility (F_x_) multiplier for raptor model stage matrices**

| **Model** | **Age 1-2** | **Age 2-3** | **Age 3-5** | **Age > 5** | **F_x_^a^** |
| --- | --- | --- | --- | --- | --- |
| Common Buzzard (*Buteo buteo*) | 0.98645 | 0.99318 | 0.98484 | 0.98484 | 0.95245 |
| Red Kite (*Milvus milvus*) | 0.98861 | 0.98861 | 0.99238 | 0.98861 | 0.96588 |
| Bearded Vulture (*Gypaetus barbatus)^b^* | 1.01105-1.01589 | 1.00124-1.00131 | 1.00124-1.00131 | 1.00227-1.00131 | 1.0331-1.0381 |

^a^ Because a pre-breeding stage matrix was used, F_x_ =fecundity * juvenile survival to age 1 in the raptor models (Meyer et al. 2016).

^b^Unlike the common buzzard and red kite, that have relatively consistent growth rates over time (red kite’s is relatively consistent because of high theta in the theta logistic density dependent equation), bearded vulture growth rate changes due to density dependence and a lower theta, which changes the multipliers as density changes, and the multipler shown represents the range from 2012 to 2062, the 50-year period evaluated.

**Definition of Our Terminology vs. Bellrose Method**

Many different terms are used to define mortality from lead shot ingestion, which can cause confusion when attempting to compare our method for gallinaceous birds to the Bellrose (1959) method. To clarify, the proportion of birds in a living population that have ingested lead shot during the short period that shot remains in a bird’s digestive tract is defined as “prevalence” of lead shot ingestion [22] (a metric used in Bellrose method), which can be corrected for hunter bias and turnover time in the digestive tract during the hunting period (assumed to be the period of exposure) to represent “annual prevalence” in the population. In contrast, our method defines the proportion of deaths caused by lead shot ingestion, *i,* as “annual carcass prevalence” of lead shot ingestion (shown in Figs. 1 and 2) based on necropsy diagnoses or liver concentrations (all direct mortality sources within a study must sum to 100%). Using the Bellrose method, Pain et al. (2019b) estimated annual prevalence as 12.4% and 7.2% for pheasants and red-legged partridges in the UK, respectively, which is not the same statistic as our annual carcass prevalence. We estimate annual carcass prevalence of gallinaceous birds in the UK as 1.6%, the midpoint of the range of UK carcass percentages (average of direct and ultimate percent for UK; Table 1), which is twice our midpoint estimate for all susceptible gallinaceous bird species across Europe (0.8%; Table 1).

Also, the percentage of carcasses with ingested lead shot in the gizzard is not the same as percentage of carcasses diagnosed as actually dying of lead shot. The latter represents our direct percentages dying from lead poisoning as a proximal cause (e.g., see Potts 2005) and the former include sublethal deaths. **The Bellrose (1959) method can best be compared to our method with the statistic “proportion of the population dying annually from lead shot ingestion,” as discussed in the main text. In the Bellrose (1959) method, the “added annual mortality” multiplied by annual prevalence produces this statistic. Bellrose (1959) defines added annual mortality as the absolute amount of annual mortality in the presence of lead shot that is above the annual mortality that would occur from all other mortality sources (note: in contrast to relative mortality, absolute mortality sources do not sum to 100%, because some birds survive, see equations below). Unlike our method in which added annual mortality is applied to the entire population in a population model, added annual mortality in the Bellrose (1959) method only applies to those birds in the population that have ingested lead shot, and thus that statistic is not comparable. The added annual mortality in the Bellrose (1959) method also includes sublethal-related mortality when based on Bellrose’s (1959) band returns in the field; thus, use of this method incorporates sublethal mortality. That is an advantage of the Bellrose (1959) method of tracking dosed birds over the long-term, because our method must assume the percent of sublethal mortality is related to tissue concentrations or lead shot in the gizzard.**

**Derivation of Equations 1 to 3 in the Main Paper and Percentage of Population Dying of Lead Shot Ingestion**

**Equation 1 for Small-bodied Raptor Survival Multiplier**

The basic model of joint, independent survival in the presence of lead shot ingestion and all other causes of mortality for small-bodied raptors combined is as follows (Bliss 1939, Borgert et al. 2001, Meyer et al. 2015):

S_total_ = S_other,alone_∙S_lead,alone_ (Eqn. S1)

where:

S_total_ = total annual survival proportion in the presence of all causes of death,

S_other,alone_ = annual survival proportion in the presence of all causes of death except lead shot ingestion, and

S_lead, alone_ = annual survival proportion in the presence of only lead shot ingestion (no deaths otherwise).

For example, if survival in a non-lead shot environment (S_other,alone_) is 0.6 (40% mortality) and lead ingestion reduces survival by 10% (S_lead,alone_ is 0.9), then the lead-reduced survival (S_total_) is 0.54. Restated in terms of mortality, Equation S1 becomes:

(1 - M_total_) = (1 – M_other,alone_)∙(1 - M_lead,alone_) (Eqn. S2)

where:

M_total_ = total annual mortality proportion in the presence of all causes of death,

M_other,alone_ = annual mortality proportion in the presence of all causes of death except lead shot ingestion, and

M_lead,alone_ = annual mortality proportion in the presence of only lead shot ingestion (no deaths otherwise).

S_lead, alone_ is the relative (rather than absolute) annual survival proportion, similar to survival standardized to controls in laboratory toxicity studies (in which controls have negligible or no mortality). The field baseline absolute annual survival of the population without lead shot ingestion (S_other,alone_) is multiplied by this relative survival (1 minus the relative mortality [M_lead,alone_]) to obtain the resultant survival with lead shot ingestion present (S_total_). In the example above, M_lead,alone_ = 0.1 and M_other,alone_ = 0.4. The relative mortality (M_lead,alone_) that reduces population survival due to lead shot ingestion must include the relative reduction in survival of birds ingesting lead shot and the proportion of the population that ingested lead shot (multiply the proportion of the population ingesting lead shot by the proportional decrease from the lead shot). For the case with raptors, relative mortality (M_lead,alone_) is not known and must be solved for with the carcass data.

Equation S2 expands to:

(1 – M_total_) = 1 – M_other,alone_ – M_lead,alone_ + M_other,alone_∙M_lead,alone_ (Eqn. S3)

Then, Equation S3 simplifies to:

M_total_ = M_other,alone_ + M_lead,alone_ – M_other,alone_∙M_lead,alone_ (Eqn. S4)

Equation S4 shows the sum of mortality from an environment with only lead shot-ingestion mortality and mortality from an environment with only all other non-lead mortalities is not the total mortality in a population with both types of mortalities**.** For example, if lead is not present in an environment (called scenario 1), each bird still has a certain probability of death from non-lead factors. If lead ingestion is added to scenario 1 (called scenario 2), some birds that otherwise would have died from non-lead factors may now die of lead shot ingestion before dying of the non-lead factors. Because those birds died in either scenario, lead did not cause additional deaths of those birds. This overlap between the two scenarios is calculated as the mathematical product of the two independent mortality factors and is then subtracted from the sum of the two independent mortality proportions to avoid double-counting.

To continue the derivation of the raptor survival multiplier, the baseline raptor population is assumed to have no lead shot ingestion. Thus, M_other,alone_ is known. M_total_ is unknown but can be calculated once M_lead,alone_ is solved for. For simplicity, we define M = M_other,alone_ as the total annual mortality proportion of the baseline population for raptors. This definition is specific to the situation with raptor populations and differs from the situation for gallinaceous-bird populations (see below).

Using that definition of M for raptors, Equation S4 can be rewritten as:

M_total_ = M + M_lead,alone_ – M ∙M_lead,alone_ (Eqn. S5)

Because we base the amount of mortality attributed to lead shot on necropsy information or on the concentration of lead in liver tissue, the proportion of deaths (carcasses) attributed to lead shot ingestion (i) is equivalent to the annual mortality proportion of the living population that would occur in the presence of only lead shot ingestion as a mortality factor (M_lead,alone_) divided by total annual mortality of the living population from all mortality sources. Thus, the proportion of the total mortality that we attribute to lead shot (i) is:

$i = \frac{M_{lead, alone}}{M_{\mathrm{total}}}$ (Eqn. S6)

Note that i is the incidence of lead shot as cause of death in a set of carcasses from a population, and is represented by the percentages in S1 and S2 Tables.

Equation S6 rearranges to:

M_lead,alone_ = i∙M_total_ (Eqn. S7)

Substitution of Equation S7 into Equation S5 produces:

M_total_ = M + i∙M_total_ - M∙i∙M_total_ (Eqn. S8)

Equation S8 rearranges to:

$M_{\mathrm{total}} = \frac{M}{1 - i + i\cdot M}$ (Eqn. S9)

Substitution of Equation S9 into Equation S7 produces M_lead,alone_:

$M_{lead,alone} = \frac{i\cdot M}{1 - i + i\cdot M}$ (Eqn. S10)

To obtain the survival multiplier, conversion of Equation S10 to a survival proportion produces:

${S_{lead,alone}=1- M}_{lead,alone} = 1- \frac{i\cdot M}{1 - i + i\cdot M}$ (Eqn. S11)

Substitution of Equation S11 into Equation S1 produces:

$S_{\mathrm{total}} = S_{other,alone}\cdot\left( 1- \frac{i\cdot M}{1 - i + i\cdot M} \right)$ (Eqn. S12)

Thus, the survival multiplier (SM) to convert from survival proportion in the absence of lead shot (S_other,alone_ , which equals S_total_ in the absence of lead shot) to total survival in the presence of lead shot is

$SM=\left( 1- \frac{i\cdot M}{1 - i + i\cdot M} \right)$ (Eqn. S13)

This multiplier is Equation 1 in the main paper. 1 - SM is relative mortality (M_lead,alone_)_._

For calculating the percent of the population dying of lead shot ingestion relative to a population with no lead shot ingestion mortality, the equation is:

M_lead,dying_prop_ = M_lead,alone_ – M_other,alone_∙M_lead,alone_  (Eqn. S14)

M_lead,dying_prop_ is the absolute, rather than the relative annual mortality in a living population from lead shot ingestion.

**Equation 2 for Gallinaceous Bird and Large-Bodied Raptor Survival Multiplier**

For gallinaceous birds, the baseline population already has lead shot exposure. Thus, i and M_total_ are known, but M_other,alone_ is unknown (i.e., the pre-lead-shot-ingestion condition is not known). For simplicity in this situation, we define M = M_total_ as the total annual mortality proportion of the baseline gallinaceous-bird population. This definition of M is specific to the situation with gallinaceous-bird and large-bodied raptor population models and differs from the situation for small-bodied raptor populations (see above).

Using that definition of M for gallinaceous birds, Equation S4 can be combined with Equation S7 to produce:

M = M_other,alone_ + i∙M – M_other,alone_∙i∙M (Eqn. S15)

Equation S15 rearranges to:

$M_{other,alone} = \frac{M - i\cdot M}{1- i\cdot M}$ (Eqn. S16)

Conversion of Equation S16 to a survival proportion produces:

$S_{other,alone}=1- M_{other,alone} = 1- \frac{M - i\cdot M}{1- i\cdot M}= \frac{1-M}{1-i\cdot M}$ = $\frac{S_{\mathrm{total}}}{1-i\cdot M}$ (Eqn. S17)

Therefore, the survival multiplier (SM) to convert from total survival in the presence of lead shot ingestion (S_total_) to survival in the absence of lead shot (S_other,aone_, which equals total survival in the absence of lead shot ingestion) is

SM = $\frac{1}{1- i\cdot M}$ (Eqn. S18)

This multiplier is Equation 2 in the main paper. Rearrangement and substitution produce:

(SM-1)/SM = M_lead,alone_ (Eqn. S19)

which is the relative mortality from lead.

To calculate the percentage of the population dying of lead shot ingestion, the equation is the same as Equation S14 (M_lead,dying_prop_ = M_lead,alone_ – M_other,alone_∙M_lead,alone_), representing the absolute annual mortality due to the lead shot ingestion.

**Equation 3 for Gallinaceous Bird Fecundity Multiplier**

By analogy for gallinaceous-bird chicks, the multiplier to convert from total chick survival in the presence of lead shot to chick survival in the absence of lead shot is 1/(1 – i_c_∙M_c_), where i_c_ = proportion of total chick deaths caused by ingestion of lead shot, and M_c_ = annual mortality proportion for chicks in the baseline population (i.e., in the absence of lead shot ingestion). If H is the proportional reduction in egg-hatching rate caused by lead shot ingestion by hens, the multiplier (FM) to convert from fecundity in the presence of lead shot ingestion to fecundity in the absence of lead shot ingestion requires inserting (1-H) into the denominator of the equation:

$FM = \frac{1}{(1 - H)\cdot(1 - i_{c}{\cdot M}_{c})}$ (Eqn. S20)

This fecundity multiplier is Equation 3 in the main paper and was used to modify F_x_ in the population model stage matrix to remove lead shot ingestion effects on reproduction.

**Comparison of Derived Equations to Other Studies**

Comparison of a graph of Equation 2 in Green et al. (2022) to our partridge/bearded vulture equation is shown in Fig. A. The comparison indicates where the two approaches differ when the objective is to estimate a population modeling scenario without lead (designated as the counterfactual scenario in Green et al. 2022). As long as the observed survival (S_total_) exceeds 0.6 and i (proportion of carcasses exceeding threshold for lead poisoning) is low (0.1 or lower), both methods produce similar results. Those two criteria fit the Green et al. (2022) models for raptors, and thus results from the two equations, if given the same data, should be almost identical. However, if survival is very low for a species in the field or even zero due to non-lead causes, we can find no obvious reason why survival in an environment with no other non-lead mortality (S_lead,alone_, such as occurs in most laboratory studies,) should be correspondingly low or zero, especially if i is low. Our equation more correctly has the y intercept equal to i, indicating that if survival in the field is 0, the portion attributable to lead is not 100% but rather is determined by the proportion of deaths attributable to lead and is independent of other, non-lead causes. Equation 3 in Green et al. (2022) is the same as ours.


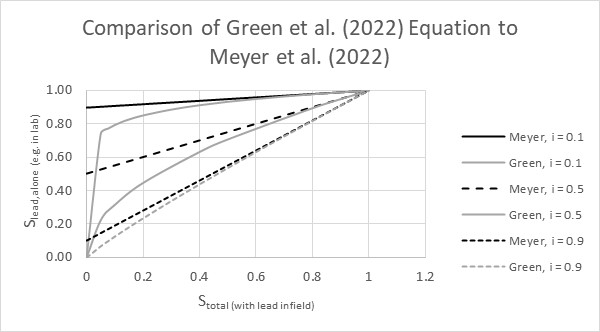


Fig. A. Relationship between survival in the field with lead ingestion (S_total_) to survival when no other mortality factors are present except lead poisoning (S_lead,alone_).

Other authors have used different methods not requiring such equations. Hanley et al. (2022) added their recorded bald eagle deaths assumed to occur from lead ingestion (based on lead concentrations exceeding clinical thresholds) back to the population to estimate losses based on the difference in the population trends. Their approach requires tracking and knowing every possible lead-based death in the studied population. Slabe et al. (2022) used the percentage of bald eagle carcasses in the USA with exceedance of severe clinical lead thresholds to reduce annual mortality (e.g., 14.1% baseline mortality was reduced to 12.3% if 12.8% of dead birds had severe clinical poisoning). Millsap et al. (2022) avoided using equations to convert carcass percentages by evaluating and modeling the western USA golden eagle population dynamics and trend and compared the trend to causes of death without attempting to model impacts of individual causes. Their purpose was different, however, focused on assessing if additional take of eagles, not lead poisoning, would cause the population to become unsustainable.

**References**

**S1 Appendix References**

Ákoshegyi I. Poisoning in waterfowl and pheasants due to lead shot in Hungary. Aquila. 2000;105–106: 47–58.

Arnemo JM, Andersen O, Stokke S, Thomas VG, Krone O, Pain DJ, et al. Health and Environmental Risks from Lead-based Ammunition: Science Versus Socio-Politics. Ecohealth. 2016;13: 618–622. doi:10.1007/s10393-016-1177-x

Arrondo E, Navarro J, Perez-García JM, Mateo R, Camarero PR, Martin-Doimeadios RCR, et al. Dust and bullets: Stable isotopes and GPS tracking disentangle lead sources for a large avian scavenger. Environ Pollut. 2020;266: 1–9. doi:10.1016/j.envpol.2020.115022

Bassi E, Facoetti R, Ferloni M, Pastorino A, Bianchi A, Fedrizzi G, et al. Lead contamination in tissues of large avian scavengers in South-Central Europe. Sci Total Environ. 2021;778: 1–13. doi:10.1016/j.scitotenv.2021.146130

Battaglia A, Ghidini S, Campanini G, Spaggiari R. Heavy metal contamination in little owl (*Athene noctua*) and common buzzard (*Buteo buteo*) from northern Italy. Ecotoxicol Environ Saf. 2005;60: 61–66. doi:10.1016/j.ecoenv.2003.12.019

Bellrose FC. Lead poisoning as a mortality factor in waterfowl populations. Ill Nat Hist Surv Bull. 1959;27: 235–288.

Berny P, Vilagines L, Cugnasse JM, Mastain O, Chollet JY, Joncour G, et al. Vigilance Poison: Illegal poisoning and lead intoxication are the main factors affecting avian scavenger survival in the Pyrenees (France). Ecotoxicol Environ Saf. 2015;118: 71–82. doi:10.1016/j.ecoenv.2015.04.003

Besnard A, Novoa C, Gimenez O. Hunting impact on the population dynamics of Pyrenean grey partridge *Perdix perdix hispaniensis*. Wildl Biol. 2010;16: 135–143. doi:10.2981/08-077

Bingham RJ, Larsen RT, Bissonette JA, Hall JO. Widespread ingestion of lead pellets by wild chukars in Northwestern Utah. Wildl Soc Bull. 2015;39: 94–102. doi:10.1002/wsb.527

Bliss CI. The toxicity of poisons applied jointly. Ann Appl Biol. 1939;26:585-615.

Borgert, CJ, Bertram P, Wells, CS, Simon, GS. Evaluating chemical interaction studies for mixture risk assessment. Hum Ecol Risk Assess. 2001;7:.259-306.

Buenestado FJ, Ferreras P, Blanco-Aguiar JA, Tortosa FS, Villafuerte R. Survival and causes of mortality among wild red‐legged partridges *Alectoris rufa* in southern Spain: Implications for conservation. Ibis (Lond 1859). 2009;151: 720–730.

Bunnefeld N, Reuman DC, Bains D, Milner-Gulland EJ. Impact of unintentional selective harvesting on the population dynamics of red grouse. J Anim Ecol. 2011;80:1258-1268.

Butler DA, Sage RB, Draycott RAH, Carroll JP, Potts D. Lead exposure in ring-necked pheasants on shooting estates in Great Britain. Wildl Soc Bull. 2005;35: 585–589.

Butler DA. Incidence of lead shot ingestion in red-legged partridges (*Alectoris rufa*) in Great Britain. Vet Rec. 2005;157: 661–662.

Carneiro M, Colaço B, Brandão R, Azorín B, Nicolas O, Colaço J, et al. Assessment of the exposure to heavy metals in Griffon vultures (*Gyps fulvus*) from the Iberian Peninsula. Ecotoxicol Environ Saf. 2015;113: 295–301. doi:10.1016/j.ecoenv.2014.12.016

Carneiro M, Colaço B, Brandão R, Ferreira C, Santos N, Soeiro V, et al. Biomonitoring of heavy metals (Cd, Hg, and Pb) and metalloid (As) with the Portuguese common buzzard (*Buteo buteo*). Environ Monit Assess. 2014;186: 7011–7021. doi:10.1007/s10661-014-3906-3

Clausen B, Wolstrup C. Lead poisoning in game from Denmark. Danish Review of Game Biology. 1979. pp. 1–22.

De Leo GA, Focardi S, Gatto M, Cattadori IM. The decline of the grey partridge in Europe: comparing demographies in traditional and modern agricultural landscapes. Ecol Modell. 2004;177: 313–335. doi:10.1016/j.ecolmodel.2003.11.017

Donazar JA, Palacios CJ, Gangoso L, Ceballos O, Gonzaíez MJ, Hiraldo F. Conservation status and limiting factors in the endangered population of Egyptian vulture (*Neophron percnopterus*) in the Canary Islands. Biol Conserv. 2002;107: 89–97. Available: www.elsevier.com/locate/biocon

Espín S, Martínez-López E, Jiménez P, María-Mojica P, García-Fernández AJ. Effects of heavy metals on biomarkers for oxidative stress in Griffon vulture (*Gyps fulvus*). Environ Res. 2014;129: 59–68. doi:10.1016/j.envres.2013.11.008

European Commission. Association of European Manufacturers of Sporting Ammunition (AFEMS) Expert Working Group (EWG). 2009. Available: https://www.afems.org/who-we-are/

Ferrandis P, Mateo R, López-Serrano FR, Martínez-Haro M, Martínez-Duro E. Lead-shot exposure in red-legged partridge (*Alectoris rufa*) on a driven shooting estate. Environ Sci Technol. 2008;42: 6271–6277. doi:10.1021/es800215y

Franson JC, Pain DJ. Lead in Birds. In: Byer WN, editor. Environmental Contaminants in Biota: Interpreting Tissue Concentrations. Boca Raton, FL: CRC Press; 2011. pp. 563–593. Available: https://digitalcommons.unl.edu/usgsstaffpub/

Franson JC. Interpretation of tissue lead residues in birds other than waterfowl. In: Beyer WN, Heinz GH, Redmon-Norwood AW, editors. Environmental Contaminants in Wildlife: Interpreting Tissue Concentrations. CRC Press; 1996. pp. 265–279.

Gangoso L, Álvarez-Lloret P, Rodríguez-Navarro AAB, Mateo R, Hiraldo F, Donázar JA. Long-term effects of lead poisoning on bone mineralization in vultures exposed to ammunition sources. Environ Pollut. 2009;157: 569–574. doi:10.1016/j.envpol.2008.09.015

Ganz K, Jenni L, Madry MM, Kraemer T, Jenny H, Jenny D. Acute and chronic lead exposure in four avian scavenger species in Switzerland. Arch Environ Contam Toxicol. 2018;75: 566–575. doi:10.1007/s00244-018-0561-7

Garcia JT, Vinuela J. El plumbismo: una primera aproximación en el caso del Milano Real. In: Viñuela J, Martí R, Ruiz A, editors. Ardeola. Madrid, Spain: El Milano Real en España; 1999. pp. 213–220.

Garcia-Fernandez AJ, Martinez-Lopez E, Romero D, Maria-Mojica P, Godino A, Jimenez P. High levels of blood lead in griffon vultures (*Gyps fulvus*) from Cazorla Natural Park (southern Spain). Environ Toxicol. 2005;20: 459–463. doi:10.1002/tox.20132

Garcia‐Fernandez AJ, Sanchez‐Garcia JA, Jimenez‐Montalban P, Luna A. Lead and cadmium in wild birds in Southeastern Spain. Environ Toxicol Chem. 1995;14: 2049–2058. doi:10.1002/etc.5620141207

Gasparik J, Venglarcik J, Slamecka J, Kropil R, Smehyl P, Kopecky J. Distribution of lead in selected organs and its effect on reproduction parameters of pheasants (*Phasianus colchicus*) after an experimental per oral administration. J Environ Sci Health A Tox Hazard Subst Environ Eng. 2012;47: 1267–1271. doi:10.1080/10934529.2012.672127

Gil-Sánchez JM, Molleda S, Sánchez-Zapata JA, Bautista J, Navas I, Godinho R, et al. From sport hunting to breeding success: Patterns of lead ammunition ingestion and its effects on an endangered raptor. Sci Total Environ. 2018;613–614: 483–491. doi:10.1016/j.scitotenv.2017.09.069

Green RE, Pain DJ, Krone O. 2022. The impact of lead poisoning from ammunition sources on raptor populations in Europe. Sci Total Environ. 2022; 1-9; doi: 10.1016/j.scitotenv.2022.154017.

Hanley BJ, Dhont AA, Forzan MJ, Bunting EM, Pokras MA, Hynes KP, Dominguez-Villegas E, Shuler KL. 2022. Environmental lead reduces the resilience of bald eagle populations. J Wild Manage. 2022;1:18.

Hernández M, Margalida A. Assessing the risk of lead exposure for the conservation of the endangered Pyrenean bearded vulture (*Gypaetus barbatus*) population. Environ Res. 2009a;109: 837–842. doi:10.1016/j.envres.2009.05.001

Hontelez LCMP, van den Dungen HM, Baars AJ. Lead and cadmium in birds in the Netherlands: A preliminary survey. Arch Environ Contam Toxicol. 1992;23: 453–456.

Isomursu M, Koivusaari J, Stjernberg T, Hirvelä-Koski V, Venäläinen ER. Lead poisoning and other human-related factors cause significant mortality in white-tailed eagles. Ambio. 2018;47: 858–868. doi:10.1007/s13280-018-1052-9

Jager LP, Rijnierse FVJ, Esselink H, Baars AJ. Biomonitoring with the buzzard Buteo buteo in the Netherlands: heavy metals and sources of variation. Journal für Ornithologie. 1996;137: 295–318.

Jenni L, Madry MM, Kraemer T, Kupper J, Naegeli H, Jenny H, et al. The frequency distribution of lead concentration in feathers, blood, bone, kidney and liver of golden eagles *Aquila chrysaetos*: Insights into the modes of uptake. J Ornithol. 2015;156: 1095–1103. doi:10.1007/s10336-015-1220-7

Kanstrup N, Chriél M, Dietz R, Søndergaard J, Balsby TJS, Sonne C. Lead and other trace elements in Danish birds of prey. Arch Environ Contam Toxicol. 2019;77: 359–367. doi:10.1007/s00244-019-00646-5

Kenntner N, Crettenand Y, Fünfstück HJ, Janovsky M, Tataruch F. Lead poisoning and heavy metal exposure of golden eagles (*Aquila chrysaetos*) from the European Alps. J Ornithol. 2007;148: 173–177.

Kenntner N, Krone O, Altenkamp R, Tataruch F. Environmental contaminants in liver and kidney of free-ranging northern goshawks (*Accipiter gentilis*) from three regions of Germany. Arch Environ Contam Toxicol. 2003;45: 128–135. doi:10.1007/s00244-002-2100-8

Kenntner N, Tataruch F, Krone O. Heavy metals in soft tissue of white-tailed eagles found dead or moribund in Germany and Austria from 1993 to 2000. Environ Toxicol Chem. 2001;20: 1831–1837.

Kerr R, Holladay S, Jarrett T, Selcer B, Meldrum B, Williams S, et al. Lead pellet retention time and associated toxicity in northern bobwhite quail (*Colinus virginianus*). Environ Toxicol Chem. 2010;29: 2869–2874. doi:10.1002/etc.355

Kitowski I, Jakubas D, Wiącek D, Sujak A, Pitucha G. Trace element concentrations in livers of common buzzards *Buteo buteo* from Eastern Poland. Environ Monit Assess. 2017;189. doi:10.1007/s10661-017-6135-8

Krone O, Kenntner N, Trinogga A, Nadjafzadeh M, Scholz F, Sulawa J, et al. Lead poisoning in White-tailed Sea Eagles: causes and approaches to solutions in Germany. In: Watson RT, Fuller M, Pokras M, Hunt WG, editors. Ingestion of Lead from Spent Ammunition: Implications for Wildlife and Humans. Boise, Idaho: The Peregrine Fund; 2009. doi:10.4080/ilsa.2009.0207

Lecocq YA. European perspective on wild game meat and public health. Rev Sci Tech . 1997;16: 579–585. doi:10.20506/rst.16.2.1041

Madry MM, Kraemer T, Kupper J, Naegeli H, Jenny H, Jenni L, et al. Excessive lead burden among golden eagles in the Swiss Alps. Environ Res Lett. 2015;10. doi:10.1088/1748-9326/10/3/034003

Madslien K, Vikoren T, Bernhoft A, Salbu B, Teien HC. Determination of lead in livers of Norwegian birds of prey during the period 1973-2014. Norwegian Veterinary Institute, Olso (in Norwegian). Oslo; 2015. Available: https://www.nmbu.no/aktuelt/node/19814

Massei G, Kindberg J, Licoppe A, Gačić D, Šprem N, Kamler J, et al. Wild boar populations up, numbers of hunters down? A review of trends and implications for Europe. Pest Manag Sci. 2015;71: 492–500. doi:10.1002/ps.3965

Mateo R. Lead Poisoning in Wild Birds in Europe and the Regulations Adopted by Different Countries. In: RT Watson MFMP and WH, editor. Ingestion of Lead from Spent Ammunition: Implications for Wildlife and Humans. Boise, Idaho, USA.: The Peregrine Fund; 2009. pp. 71–98. doi:10.4080/ilsa.2009.0107

Mateo R, Cadenas R, Máñez M, Guitart R. Lead shot ingestion in two raptor species from Doñana, Spain. Ecotoxicol Environ Saf. 2001;48: 6–10. doi:10.1006/eesa.2000.1996

Mateo R, Kanstrup N. Regulations on lead ammunition adopted in Europe and evidence of compliance. Ambio. 2019;48: 989–998. doi:10.1007/s13280-019-01170-5

Mateo R, Rodríguez-de la Cruz M, Vidal D, Reglero M, Camarero P. Transfer of lead from shot pellets to game meat during cooking. Sci Total Environ. 2007;372: 480–485. doi:10.1016/j.scitotenv.2006.10.022

Mateo-Tomás P, Olea PP, Jiménez-Moreno M, Camarero PR, Sánchez-Barbudo IS, Rodríguez Martín-Doimeadios RC, et al. Mapping the spatio-temporal risk of lead exposure in apex species for more effective mitigation. Proceedings of the Royal Society B: Biological Sciences. 2016;283: 201–262. doi:10.1098/rspb.2016.0662

Mateo-Tomás P, Olea PP, Jiménez-Moreno M, Camarero PR, Sánchez-Barbudo IS, Rodríguez Martín-Doimeadios RC, et al. Mapping the spatio-temporal risk of lead exposure in apex species for more effective mitigation. Proceedings of the Royal Society B: Biological Sciences. 2016;283. doi:10.1098/rspb.2016.0662

Meyer CB, Meyer JS, Francisco AB, Holder J, Verdonck F. Can ingestion of lead shot and poisons change population trends of three European birds: Grey partridge, common buzzard, and red kite? PLoS ONE. 2016;11. doi:10.1371/journal.pone.0147189

Meyer JS, Farley, KJ, Garman, ER. Metal mixture modeling evaluation: Metal mixtures modeling evaluation project: 1. Background. Environ Toxicol Chem. 2015; 34:726-740.

Millsap BA, Zimmerman GS, Kendall WL, Barnes JG, Braham MA, Bedrosian BE, et al. A. Age-specific survival rates, causes of death, and allowable take of golden eagles in the western United States. Ecol Appl. 2022;Apr;32(3):e2544. doi: 10.1002/eap.2544.

Monclús L, Shore RF, Krone O. Lead contamination in raptors in Europe: A systematic review and meta-analysis. Sci Total Environ. 2020;748. doi:10.1016/j.scitotenv.2020.141437

Nadjafzadeh M, Hofer H, Krone O. The link between feeding ecology and lead poisoning in white-tailed eagles. J Wildl Manage. 2013;77: 48–57. doi:10.1002/jwmg.440

PACEC. Economic and Environmental Impact of Sporting Shooting in the UK. Cambridge, UK; 2006 Aug. Available: www.pacec.co.uk

Pain DJ, Amiard-Triquet C. Lead poisoning of raptors in France and elsewhere. Ecotoxicol Environ Saf. 1993;25: 183–192.

Pain DJ, Carter I, Sainsbury AW, Shore RF, Eden P, Taggart MA, et al. Lead contamination and associated disease in captive and reintroduced red kites *Milvus milvus* in England. Sci Total Environ. 2007;376: 116–127. doi:10.1016/j.scitotenv.2007.01.062

Pain DJ, Mateo R, Green RE. Effects of lead from ammunition on birds and other wildlife: A review and update. Ambio. 2019a;48: 935–953. doi:10.1007/s13280-019-01159-0

Pain DJ, Dickie I, Green RE, Kanstrup N, Cromie R. Wildlife, human and environmental costs of using lead ammunition: an economic review and analysis. Ambio 2019b;48:969-988. doi:10.1007/s13280-019-01157-2

Pain DJ, Sears J, Newton I. Lead concentrations in birds of prey in Britain. Environ Pollut. 1995;87: 173–180.

Pérez-López M, Hermoso de Mendoza M, López Beceiro A, Soler Rodríguez F. Heavy metal (Cd, Pb, Zn) and metalloid (As) content in raptor species from Galicia (NW Spain). Ecotoxicol Environ Saf. 2008;70: 154–162. doi:10.1016/j.ecoenv.2007.04.016

Potts GR. Incidence of ingested lead gunshot in wild grey partridges (*Perdix perdix*) from the UK. Eur J Wildl Res. 2005;51: 31–34. doi:10.1007/s10344-004-0071-y

Reitan M, Munro Jenssen B, Nygård T. Assessing the diet of the golden eagle (*Aquila chrysaetos*) and the biomagnification of metals by use of stable isotope analysis and ICP-MS. 2013.

Robertson PA, Dowell Simon. The effects of hand-rearing on wild gamebird populations. The Future of Wild Galliformes in the Netherlands. 1990. pp. 158–171.

Rodrigue J, McNicoll R, Leclair D, Duchesne JF. Lead concentrations in ruffed grouse, rock ptarmigan, and willow ptarmigan in Quebec. Arch Environ Contam Toxicol. 2005;49: 97–104.

Romero D, de José A, Theureau JM, Ferrer A, Raigón MD, Torregrosa JB. Lead in terrestrial game birds from Spain. Environ Sci Pollut Res Int. 2020;27: 1585–1597. doi:10.1007/s11356-019-06827-y

Runia TJ, Solem AJ. Pheasant response to lead ingestion. The Prairie Naturalist. 2017;49: 13–17. Available: https://digitalcommons.unl.edu/tpn

Scanlon PF. Wet and dry weight relationships of Mallard (*Anas platyrhynchos*) tissues. Bull Environ Contam Toxicol. 1982;29: 615–617. doi:10.1007/bf01669630

Slabe VA, Anderson JT, Millsap BA, Cooper JL, Harmata AR, Restani M, et al. Implications of lead poisoning for eagles across North America. Science. 2022; 375:779-782.

Soler-Rodriguez F, Oropesa-Jimenez AL, Garcia-Cambero JP, Perez-Lopez M. Lead exposition by gunshot ingestion in red-legged partridge (*Alectoris rufa*). Vet Hum Toxicol. 2004;46: 133–134.

Taggart MA, Shore RF, Pain DJ, Peniche G, Martinez-Haro M, Mateo R, et al. Concentration and origin of lead (Pb) in liver and bone of Eurasian buzzards (*Buteo buteo*) in the United Kingdom. Environ Pollut. 2020;267. doi:10.1016/j.envpol.2020.115629

Thomas VG, Guitart R. Limitations of European Union policy and law for regulating use of lead shot and sinkers: Comparisons with North American regulation. Environmental Policy and Governance. 2009;20: 57–72. doi:10.1002/eet.527

Thomas VG, Scheuhammer AM, Bond DE. Bone lead levels and lead isotope ratios in red grouse from Scottish and Yorkshire moors. Sci Total Environ. 2009;407: 3494–3502. doi:10.1016/j.scitotenv.2009.02.003

Vallverdú-Coll N, Mougeot F, Ortiz-Santaliestra ME, Castaño C, Santiago-Moreno J, Mateo R. Effects of lead exposure on sperm quality and reproductive success in an avian model. Environ Sci Technol. 2016;50: 12484–12492. doi:10.1021/acs.est.6b04231

Vallverdú-Coll N, Ortiz-Santaliestra ME, Mougeot F, Vidal D, Mateo R. Sublethal Pb exposure produces season-dependent effects on immune response, oxidative balance and investment in carotenoid-based coloration in red-legged partridges. Environ Sci Technol. 2015;49: 3839–3850. doi:10.1021/es505148d

Walker LA, Chaplow JS, Lawlor AJ, Pereira MG, Potter ED, Sainsbury AW, et al. Lead (Pb) and Mercury (Hg) concentrations in predatory bird livers 2012: A predatory bird monitoring scheme (PBMS) report. Lancaster, UK; 2014.

Warren P, Baines D. Dispersal distances of juvenile radiotagged Red Grouse *Lagopus lagopus scoticus* on moors in northern England. Ibis (Lond 1859). 2007;149: 758–762. doi:10.1111/j.1474-919X.2007.00705.x

Watson M, Aebischer NJ, Potts GR, Ewald JA. The relative effects of raptor predation and shooting on overwinter mortality of grey partridges in the United Kingdom. J Appl Ecol. 2007;44: 972–982.

**S1 Table References**

Ákoshegyi I. Poisoning in waterfowl and pheasants due to lead shot in Hungary. Aquila. 2000;105–106: 47–58.

Alleva E, Francia N, Pandolfi M, de Marinis AM, Chiarotti F, Santucci D. Organochlorine and heavy-metal contaminants in wild mammals and birds of Urbino-Pesaro Province, Italy: An analytic overview for potential bioindicators. Arch Environ Contam Toxicol. 2006;51: 123–134. doi:10.1007/s00244-005-0218-1

Besnard A, Novoa C, Gimenez O. Hunting impact on the population dynamics of Pyrenean grey partridge Perdix perdix hispaniensis. Wildl Biol. 2010;16: 135–143. doi:10.2981/08-077

Bro E, Reitz F, Clobert J, Migot P, Massot M. Diagnosing the environmental causes of the decline in Grey Partridge Perdix perdix survival in France. Ibis (Lond 1859). 2001;143: 120–132.

Buenestado FJ, Ferreras P, Blanco-Aguiar JA, Tortosa FS, Villafuerte R. Survival and causes of mortality among wild red‐legged partridges Alectoris rufa in southern Spain: Implications for conservation. Ibis (Lond 1859). 2009;151: 720–730.

Buner F, Schaub M. How do different releasing techniques affect the survival of reintroduced grey partridges Perdix perdix? Wildl Biol. 2008;14: 26–35. doi:10.2981/0909-6396

Buner FD, Browne SJ, Aebischer NJ. Experimental assessment of release methods for the re-establishment of a red-listed galliform, the grey partridge (Perdix perdix). Biol Conserv. 2011;144: 593–601. doi:10.1016/j.biocon.2010.10.017

Butler DA. Incidence of lead shot ingestion in red-legged partridges (Alectoris rufa) in Great Britain. Vet Rec. 2005;157: 661–662.

Clausen B, Wolstrup C. Lead poisoning in game from Denmark. Danish Review of Game Biology. 1979. pp. 1–22.

Curland N, Gethöffer F, van Neer A, Ziegler L, Heffels-Redmann U, Lierz M, et al. Investigation into diseases in free-ranging ring-necked pheasants (Phasianus colchicus) in northwestern Germany during population decline with special reference to infectious pathogens. Eur J Wildl Res. 2018;64. doi:10.1007/s10344-018-1173-2

Draycott R. Game & Wildlife Management. Fordingbridge, Hampshire; 2013. Available: www.gwct.org.uk/advisory

Ferrandis P, Mateo R, López-Serrano FR, Martínez-Haro M, Martínez-Duro E. Lead-shot exposure in red-legged partridge (Alectoris rufa) on a driven shooting estate. Environ Sci Technol. 2008;42: 6271–6277. doi:10.1021/es800215y

Gaudioso VR, Sánchez-García C, Pérez JA, Rodríguez PL, Armenteros JA, Alonso ME. Does early antipredator training increase the suitability of captive red-legged partridges (Alectoris rufa) for releasing? Poult Sci. 2011;90: 1900–1908. doi:10.3382/ps.2011-01430

Hudson PJ, Newborn David, Robertson PA. Geographical and seasonal patterns of mortality in red grouse Lagopus lagopus scoticus populations. Wildl Biol. 1997;3: 79–87.

Meriggi A, Brangi A, Cuccus P, Stella RMD. High mortality rate in a re‐introduced grey partridge population in central Italy. Ital J Zool (Modena). 2002;69: 19–24.

Millot F, Berny P, Decors A, Bro E. Little field evidence of direct acute and short-term effects of current pesticides on the grey partridge. Ecotoxicol Environ Saf. 2015;117: 41–61. doi:10.1016/j.ecoenv.2015.03.017

Millot F, Decors A, Mastain O, Quintaine T, Berny P, Vey D, et al. Field evidence of bird poisonings by imidacloprid-treated seeds: a review of incidents reported by the French SAGIR network from 1995 to 2014. Environ Sci Pollut Res. 2017;24: 5469–5485. doi:10.1007/s11356-016-8272-y

Parish DMB, Sotherton NW. The fate of released captive-reared grey partridges Perdix perdix: implications for reintroduction programmes. Wildl Biol. 2007;13: 140–149.

Potts G. Partridges: Countryside Barometer. New Naturalist Series, editor. London, UK: Harper Collins Publishers Ltd.; 2012.

Potts GR. Incidence of ingested lead gunshot in wild grey partridges (Perdix perdix) from the UK. Eur J Wildl Res. 2005;51: 31–34. doi:10.1007/s10344-004-0071-y

Reiten M. Assessing the diet of the golden eagle (Aquila chrysaetos) and the biomagnification of metals by use of stable isotope analysis and ICP-MS. 2013.

Romero D, de José A, Theureau JM, Ferrer A, Raigón MD, Torregrosa JB. Lead in terrestrial game birds from Spain. Environ Sci Pollut Res Int. 2020;27: 1585–1597. doi:10.1007/s11356-019-06827-y

Soler-Rodriguez F, Oropesa-Jimenez AL, Garcia-Cambero JP, Perez-Lopez M. Lead exposition by gunshot ingestion in red-legged partridge (Alectoris rufa). Vet Hum Toxicol. 2004;46: 133–134.

Thomas VG, Scheuhammer AM, Bond DE. Bone lead levels and lead isotope ratios in red grouse from Scottish and Yorkshire moors. Sci Total Environ. 2009;407: 3494–3502. doi:10.1016/j.scitotenv.2009.02.003

**S2 Table References**

Alleva E, Francia N, Pandolfi M, de Marinis AM, Chiarotti F, Santucci D. Organochlorine and heavy-metal contaminants in wild mammals and birds of Urbino-Pesaro Province, Italy: An analytic overview for potential bioindicators. Arch Environ Contam Toxicol. 2006;51: 123–134. doi:10.1007/s00244-005-0218-1

Arrondo E, Navarro J, Perez-García JM, Mateo R, Camarero PR, Martin-Doimeadios RCR, et al. Dust and bullets: Stable isotopes and GPS tracking disentangle lead sources for a large avian scavenger. Environ Pollut. 2020;266: 1–9. doi:10.1016/j.envpol.2020.115022

Bassi E, Facoetti R, Ferloni M, Pastorino A, Bianchi A, Fedrizzi G, et al. Lead contamination in tissues of large avian scavengers in South-Central Europe. Sci Total Environ. 2021;778: 1–13. doi:10.1016/j.scitotenv.2021.146130

Battaglia A, Ghidini S, Campanini G, Spaggiari R. Heavy metal contamination in little owl (*Athene noctua*) and common buzzard (*Buteo buteo*) from northern Italy. Ecotoxicol Environ Saf. 2005;60: 61–66. doi:10.1016/j.ecoenv.2003.12.019

Bro E., Millot F., Delorme F, Polve C, Mangin E, Godard A, et al. PeGASE, bilan synthetique d’une etude perdrix grise <<population-environment>>. Faune Sauvag. 298:17-48.

Berny P, Gaillet J-R. Acute poisoning of red kites (*Milvus milvus*) in France: Data from the sagir network. J Wildl Dis. 2008;44: 417–426. Available: www.srpv-auvergne.com

Berny P, Vilagines L, Cugnasse JM, Mastain O, Chollet JY, Joncour G, et al. Vigilance Poison: Illegal poisoning and lead intoxication are the main factors affecting avian scavenger survival in the Pyrenees (France). Ecotoxicol Environ Saf. 2015;118: 71–82. doi:10.1016/j.ecoenv.2015.04.003

Carneiro M, Colaço B, Brandão R, Ferreira C, Santos N, Soeiro V, et al. Biomonitoring of heavy metals (Cd, Hg, and Pb) and metalloid (As) with the Portuguese common buzzard (*Buteo buteo*). Environ Monit Assess. 2014;186: 7011–7021. doi:10.1007/s10661-014-3906-3

Clausen B, Wolstrup C. Lead poisoning in game from Denmark. Danish Review of Game Biology. 1979. pp. 1–22.

Donazar JA, Palacios CJ, Gangoso L, Ceballos O, Gonzaíez MJ, Hiraldo F. Conservation status and limiting factors in the endangered population of Egyptian vulture (*Neophron percnopterus*) in the Canary Islands. Biol Conserv. 2002;107: 89–97. Available: www.elsevier.com/locate/biocon

Ecke F, Singh NJ, Arnemo JM, Bignert A, Helander B, Berglund ÅMM, et al. Sublethal lead exposure alters movement behavior in free-ranging golden eagles. Environ Sci Technol. 2017;51: 5729–5736. doi:10.1021/acs.est.6b06024

Gangoso L, Álvarez-Lloret P, Rodríguez-Navarro AAB, Mateo R, Hiraldo F, Donázar JA. Long-term effects of lead poisoning on bone mineralization in vultures exposed to ammunition sources. Environ Pollut. 2009;157: 569–574. doi:10.1016/j.envpol.2008.09.015

Ganz K, Jenni L, Madry MM, Kraemer T, Jenny H, Jenny D. Acute and chronic lead exposure in four avian scavenger species in Switzerland. Arch Environ Contam Toxicol. 2018;75: 566–575. doi:10.1007/s00244-018-0561-7

García-Fernández AJ, Motas-Guzmán M, Navas I, María-Mojica P, Luna A, Sánchez-García JA. Environmental exposure and distribution of lead in four species of raptors in Southeastern Spain. Arch Environ Contam Toxicol. 1997;33: 76–82.

Hernández M, Margalida A. Assessing the risk of lead exposure for the conservation of the endangered Pyrenean bearded vulture (*Gypaetus barbatus*) population. Environ Res. 2009;109: 837–842. doi:10.1016/j.envres.2009.05.001

Hontelez LCMP, van den Dungen HM, Baars AJ. Lead and cadmium in birds in the Netherlands: A preliminary survey. Arch Environ Contam Toxicol. 1992;23: 453–456.

Jager LP, Rijnierse FVJ, Esselink H, Baars AJ. Biomonitoring with the buzzard Buteo buteo in the Netherlands: heavy metals and sources of variation. Journal für Ornithologie. 1996;137: 295–318.

Jenni L, Madry MM, Kraemer T, Kupper J, Naegeli H, Jenny H, et al. The frequency distribution of lead concentration in feathers, blood, bone, kidney and liver of golden eagles *Aquila chrysaetos*: Insights into the modes of uptake. J Ornithol. 2015;156: 1095–1103. doi:10.1007/s10336-015-1220-7

Kenntner N, Crettenand Y, Fünfstück HJ, Janovsky M, Tataruch F. Lead poisoning and heavy metal exposure of golden eagles (*Aquila chrysaetos*) from the European Alps. J Ornithol. 2007;148: 173–177.

Kenntner N, Krone O, Altenkamp R, Tataruch F. Environmental contaminants in liver and kidney of free-ranging northern goshawks (*Accipiter gentilis*) from three regions of Germany. Arch Environ Contam Toxicol. 2003;45: 128–135. doi:10.1007/s00244-002-2100-8

Kenward RE, Marcström V, Karlbom M. Causes of death in radio-tagged northern goshawks. Raptor Biomedicine. Redig P.T., Cooper J.E., Remple D.J., Hunter D.B., editors. Minneapolis, MN, USA: University Press; 1993.

Kenward RE, Walls SS, Hodder KH, Pahkala M, Freeman SN, Simpson VR. The prevalence of non-breeders in raptor populations: evidence from rings, radio-tags and transect surveys. Oikos. 2000;91: 271–279.

Kitowski I, Jakubas D, Wiącek D, Sujak A, Pitucha G. Trace element concentrations in livers of common buzzards *Buteo buteo* from Eastern Poland. Environ Monit Assess. 2017;189. doi:10.1007/s10661-017-6135-8

Kitowski I, Sujak A, Wiącek D, Strobel W, Komosa A, Stobiński M. Heavy metals in livers of raptors from Eastern Poland-the importance of diet composition. Belg J Zool. 2016;146: 3–13.

Madry MM, Kraemer T, Kupper J, Naegeli H, Jenny H, Jenni L, et al. Excessive lead burden among golden eagles in the Swiss Alps. Environ Res Lett. 2015;10. doi:10.1088/1748-9326/10/3/034003

Madslien K, Vikoren T, Bernhoft A, Salbu B, Teien HC. Determination of lead in livers of Norwegian birds of prey during the period 1973-2014. Norwegian Veterinary Institute, Olso (in Norwegian). Oslo; 2015. Available: https://www.nmbu.no/aktuelt/node/19814

Molenaar FM, Jaffe JE, Carter I, Barnett EA, Shore RF, Marcus Rowcliffe J, et al. Poisoning of reintroduced red kites (*Milvus Milvus*) in England. Eur J Wildl Res. 2017;63: 94: 1–8. doi:10.1007/s10344-017-1152-z

Molina-López RA, Casal J, Darwich L. Causes of morbidity in wild raptor populations admitted at a wildlife rehabilitation centre in Spain from 1995-2007: A long term retrospective study. PLoS ONE. 2011;6: e24603. doi:10.1371/journal.pone.0024603

Pain DJ, Amiard-Triquet C. Lead poisoning of raptors in France and elsewhere. Ecotoxicol Environ Saf. 1993;25: 183–192.

Pain DJ, Carter I, Sainsbury AW, Shore RF, Eden P, Taggart MA, et al. Lead contamination and associated disease in captive and reintroduced red kites *Milvus milvus* in England. Sci Total Environ. 2007;376: 116–127. doi:10.1016/j.scitotenv.2007.01.062

Pain DJ, Sears J, Newton I. Lead concentrations in birds of prey in Britain. Environ Pollut. 1995;87: 173–180.

Palacios C-J. Current status and distribution of birds of prey in the Canary Islands. Bird Conserv Int. 2004;14: 203–213. doi:10.1017/S0959270904000255

Rodriguez B, Rodriguez A, Siverio F, Siverio M. Causes of raptor admissions to a wildlife rehabilitation center in Tenerife (Canary Islands). J Raptor Res. 2010;44: 30–39.

Sansom A, Etheridge B, Smart J, Roos S. Population modelling of North Scotland red kites in relation to the cumulative impacts of wildlife crime and wind farm mortality. Inverness; 2016.

Taggart MA, Shore RF, Pain DJ, Peniche G, Martinez-Haro M, Mateo R, et al. Concentration and origin of lead (Pb) in liver and bone of Eurasian buzzards (*Buteo buteo*) in the United Kingdom. Environ Pollut. 2020;267. doi:10.1016/j.envpol.2020.115629

Walker LA, Chaplow JS, Lawlor AJ, Pereira MG, Potter ED, Sainsbury AW, et al. Lead (Pb) and Mercury (Hg) concentrations in predatory bird livers 2012: A predatory bird monitoring scheme (PBMS) report. Lancaster, UK; 2014.

Xirouchakis S. Causes of Raptor Mortality in Crete. Heraklion, Crete, Greece: Natural History Museum of Crete, University of Crete; 2004.

Zaccaroni A, Andreani G, Ferrante MC, Carpenè E, Isani G, Lucisano A. Metal concentrations in the liver and kidney of raptor species from the Calabria region, Italy. Acta Vet Brno. 2008;58: 315–324. doi:10.2298/AVB0804315Z
