# Supplementary material for: Method to assess the potential magnitude of terrestrial European avian population reductions from ingestion of lead ammunition
Source: PLoS One. 2022 Aug 29;17(8):e0273572. doi: 10.1371/journal.pone.0273572 (PMC9423653; doi:10.1371/journal.pone.0273572)
Supplement: S2 Appendix — (DOCX) [file pone.0273572.s005.docx]

**S2 Appendix: Bearded Vulture Population Model**

This appendix describes development and application of the bearded vulture population model. We modified the buzzard model we had developed in Poptools and R in Meyer et al. (2016) for the bearded vulture. We incorporated age-specific vital rates (survival and reproduction), standard deviation on the vital rates, density dependence, and carrying capacity reported in Margalida et al. (2020) for the Pyrenees. The majority of the European population is in the Pyrenees, which has exposure to lead ammunition (S3 Table). Therefore, the equations for the multipliers were those that remove lead poisoning. We calibrated the model on the actual population trend in the Pyrenees from 1987 to 2016.

We entered mean survival and fertility into a one-sex, pre-breeding Leslie transition matrix [49]. The matrix was multiplied by a vector of age-class-specific population sizes:

$\left[ \begin{matrix} N_{1} \\ N_{2} \\ N_{3} \\ N_{4} \\ N_{5} \\ N_{6} \\ N_{7} \\ N_{8} \\ N_{9} \\ N_{10} \\ N_{11} \\ N_{12} \\ N_{13} \\ N_{14} \\ N_{15} \\ N_{16+} \end{matrix} \right]_{t+1}$=$\left[ \begin{matrix} F_{1} & F_{2} & F_{3} & F_{4} & F_{5} & F_{6} & F_{7} & F_{8} & F_{9} & F_{10} & F_{11} & F_{12} & F_{13} & F_{14} & F_{15} & F_{16} \\ S_{1} & 0 & 0 & 0 & 0 & 0 & 0 & 0 & 0 & 0 & 0 & 0 & 0 & 0 & 0 & 0 \\ 0 & S_{2} & 0 & 0 & 0 & 0 & 0 & 0 & 0 & 0 & 0 & 0 & 0 & 0 & 0 & 0 \\ 0 & 0 & S_{3} & 0 & 0 & 0 & 0 & 0 & 0 & 0 & 0 & 0 & 0 & 0 & 0 & 0 \\ 0 & 0 & 0 & S_{4} & 0 & 0 & 0 & 0 & 0 & 0 & 0 & 0 & 0 & 0 & 0 & 0 \\ 0 & 0 & 0 & 0 & S_{5} & 0 & 0 & 0 & 0 & 0 & 0 & 0 & 0 & 0 & 0 & 0 \\ 0 & 0 & 0 & 0 & 0 & S_{6} & 0 & 0 & 0 & 0 & 0 & 0 & 0 & 0 & 0 & 0 \\ 0 & 0 & 0 & 0 & 0 & 0 & S_{7} & 0 & 0 & 0 & 0 & 0 & 0 & 0 & 0 & 0 \\ 0 & 0 & 0 & 0 & 0 & 0 & 0 & S_{8} & 0 & 0 & 0 & 0 & 0 & 0 & 0 & 0 \\ 0 & 0 & 0 & 0 & 0 & 0 & 0 & 0 & S_{9} & 0 & 0 & 0 & 0 & 0 & 0 & 0 \\ 0 & 0 & 0 & 0 & 0 & 0 & 0 & 0 & 0 & S_{10} & 0 & 0 & 0 & 0 & 0 & 0 \\ 0 & 0 & 0 & 0 & 0 & 0 & 0 & 0 & 0 & 0 & S_{11} & 0 & 0 & 0 & 0 & 0 \\ 0 & 0 & 0 & 0 & 0 & 0 & 0 & 0 & 0 & 0 & 0 & S_{12} & 0 & 0 & 0 & 0 \\ 0 & 0 & 0 & 0 & 0 & 0 & 0 & 0 & 0 & 0 & 0 & 0 & S_{13} & 0 & 0 & 0 \\ 0 & 0 & 0 & 0 & 0 & 0 & 0 & 0 & 0 & 0 & 0 & 0 & 0 & S_{14} & 0 & 0 \\ 0 & 0 & 0 & 0 & 0 & 0 & 0 & 0 & 0 & 0 & 0 & 0 & 0 & 0 & S_{15} & S_{16+} \end{matrix} \right]$$\left[ \begin{matrix} N_{1} \\ N_{2} \\ N_{3} \\ N_{4} \\ N_{5} \\ N_{6} \\ N_{7} \\ N_{8} \\ N_{9} \\ N_{10} \\ N_{11} \\ N_{12} \\ N_{13} \\ N_{14} \\ N_{15} \\ N_{16+} \end{matrix} \right]_{t}$

where N_x,t_ is number of x-year-old females at time t (i.e., N_1,t_ is number of 1-year-old females at time t). Survival probabilities of a female of age x between time t and t+1 are denoted as S_x_, and fertility (females at time t+1 that were produced by females at time t) as F_x_. For the oldest year class, we combined all adults age 16 and older, which technically converts the Leslie matrix to a 16 x 16 Lefkovitch matrix [49]. Using an integrated population model (IPM) on the field-collected data that tracked birds and nests through time, Margalida et al. (2020) calculated the following parameters for each age class: mean reproductive success (number of fledglings produced per breeding pair or territory), mean probability of a subadult becoming a breeder (recruitment), mean probability of an experienced breeder breeding, and mean annual survival (Table 1). Age of first breeding is 6 and all subadults have recruited to become breeders by age 16. We calculated the cumulative probability of a subadult breeding in each age class to estimate the proportion of subadults and adults breeding and then applied the subadult recruitment rate to the subadult proportion and the adult experienced breeding probability to the adult portion to obtain the breeding probability for each age class (Table 1). To obtain fecundity (m_x_), we multiplied the breeding probability by reproductive success x 0.5 (x 0.5 to convert fledglings to female fledglings for one sex model). Then we multiplied fecundity by survival of juveniles to age 1 to obtain fertility for each age class (F_x_). We entered the age-specific mean fertility (F_x_) and survival (S_x_) rates into the matrix.

Because the matrix has mean values, the matrix approximates a mean growth rate over the 25 years of monitoring, rather than the initial growth rate in 1987. 1987 was the first year of the monitoring and population trend data and the starting year of the model to be calibrated. To adjust the matrix to represent vital rates in 1987 as the starting matrix, we applied the density-dependent equations on reproductive success and survival provided in Margalida et al. (2020) to the corresponding vital rate, after first converting their standardized total population size (normalized by subtracting the mean and dividing by the standard deviation) used in their equations to actual population size. Fig. A shows the standardized version provided in the paper and our conversion. We used the total population size reported for 1987 in these equations to develop the model’s initial stage matrix and applied the stable age distribution of the matrix to the total population size to estimate starting sizes in each age class in 1987. We added stochasticity by modeling variability around the density-dependent coefficients, using the standard deviations for those coefficients with a normal distribution around the mean. Standard deviations were used that produced the variability observed in annual survival and reproductive success shown in the graphs in Margalida et al. (2020). The coefficients of the density-dependent equations were applied each year as the population changed to adjust the vital rates in the matrix. We ran this baseline model that includes lead exposure 10,000 times to obtain the mean modeled population trajectory for 50 years. We modeled total population size and breeding territory trajectories (1 territory = 1 breeding female). To estimate total breeders, the number of territories are multiplied by 2.28 to account for the number of polyandrous triplets in territories observed in the Pyrenees on average.

Because the vital rates estimated in Margalida et al. (2020) do not account for any immigration or emigration, they need to be calibrated to the actual observed population trend. We calibrated the vital rates in the model by comparing the mean trajectory and its 95% confidence intervals to the actual population trajectory produced by the IPM (which removes sampling error) from 1987 to 2016. We also compared it to typical single run trajectories to evaluate the variability. The density-dependent vital rates in Margalida et al. (2020) caused the trajectory to reach equilibrium earlier than observed (the population had not reached equilibrium even in 2016), and therefore we decreased the slope of the equation for reproductive success, changing the coefficient from -0.00191 to -0.00141. The standard deviations on the coefficients replicated the observed total population size variability. Fig. B shows the final calibrated model results compared to the observed total and breeding pair/triplet population trend. For simplicity, the main text refers to a pair/triplet as a pair for the bearded vulture (e.g., occupied breeding territory).

After calibrating, we ran the baseline model 10,000 times for 50 years, starting in 2012. Then we applied survival and fertility multipliers (S1 Appendix) to remove lead poisoning effects (increases the vital rates) and re-ran the model 10,000 times. We estimated the change in the mean population size in the first 50 years of a growing population and the proportion of runs in which the population size dropped below our quasi-extinction threshold, set to a total population size of 900 birds. We used the Sibly et al. (2005) theta-logistic method to estimate the maximum population growth rate, carrying capacity, and shape of the density-dependence (deterministic baseline maximum growth rate = 1.077, baseline theta = 2.2) of the mean modeled sigmoid growth curve for both the baseline and without lead (counterfactual) scenarios.

To evaluate lead poisoning losses to the population when at equilibrium, we applied the multipliers in the year 2050 and ran the model with and without lead ingestion effects to evaluate change over the 50-year period starting in 2050. Multipliers were developed by inserting the midpoint, lower and upper bound proportion of carcasses with potential lead poisoning, *i*, in the equations in the S1 Appendix for large-bodied raptors.

To clarify how our approach of focusing on cause of death must sum to 100%, we provide an example of the possible estimated upper and lower bound on the percentage of bearded vulture deaths that is from lead poisoning relative to other causes for the individual bearded vulture analysis in Europe in Fig. C. The upper bound proportion includes sublethal deaths and therefore deaths from other causes must be reduced (e.g., we removed it from collisions in this case as an example, after assigning part of unintentional poisoning to our estimates of lead poisoning percentages in carcasses). In Fig. C, the range in the proportion of deaths from lead poisoning is for bearded vultures in Europe (i = 3.42 to 7.17%), which is not that different than the estimate when all susceptible raptor species are combined (i = 2.52 to 7.9%).

Table 1. Parameters used to calculate the stage matrix with mean values of survival (F_x_) and fertility (S_x_) for the bearded vulture in the Pyrenees.

| **Age Class** | **Mean Survival (S_x_)^a^** | **Mean Recruitment Probability** | **Mean Cumulative Recruitment Transition Probability** | **Mean Breeding Probability** | **Mean Reproductive Success** | **Mean Fecundity (m_x_)** | **Mean Fertility  (F_x_ = S_1_ _*_ m_x_)** |
| --- | --- | --- | --- | --- | --- | --- | --- |
| 1 | 0.947 | 0.000 | 0.00 | 0.000 | 0.000 | 0.000 | 0.000 |
| 2 | 0.947 | 0.000 | 0.00 | 0.000 | 0.000 | 0.000 | 0.000 |
| 3 | 0.966 | 0.000 | 0.00 | 0.000 | 0.000 | 0.000 | 0.000 |
| 4 | 0.966 | 0.000 | 0.00 | 0.000 | 0.000 | 0.000 | 0.000 |
| 5 | 0.966 | 0.000 | 0.00 | 0.000 | 0.000 | 0.000 | 0.000 |
| 6 | 0.966 | 0.067 | 0.06 | 0.040 | 0.413 | 0.008 | 0.008 |
| 7 | 0.968 | 0.058 | 0.12 | 0.094 | 0.413 | 0.019 | 0.018 |
| 8 | 0.968 | 0.052 | 0.17 | 0.121 | 0.413 | 0.025 | 0.024 |
| 9 | 0.968 | 0.255 | 0.41 | 0.344 | 0.413 | 0.071 | 0.067 |
| 10 | 0.968 | 0.196 | 0.58 | 0.430 | 0.413 | 0.089 | 0.084 |
| 11 | 0.968 | 0.108 | 0.67 | 0.449 | 0.413 | 0.093 | 0.088 |
| 12 | 0.968 | 0.153 | 0.80 | 0.543 | 0.413 | 0.112 | 0.106 |
| 13 | 0.968 | 0.081 | 0.85 | 0.548 | 0.413 | 0.113 | 0.107 |
| 14 | 0.968 | 0.108 | 0.93 | 0.606 | 0.413 | 0.125 | 0.118 |
| 15 | 0.968 | 0.108 | 1.00 | 0.650 | 0.413 | 0.134 | 0.127 |
| 16+ | 0.968 | 0.108 | 1.00 | 0.622 | 0.413 | 0.128 | 0.122 |


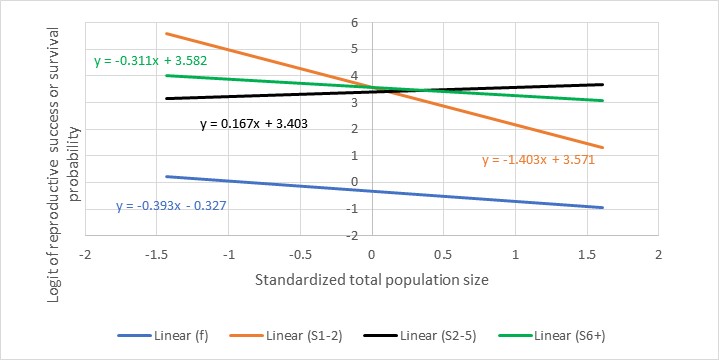


**a**


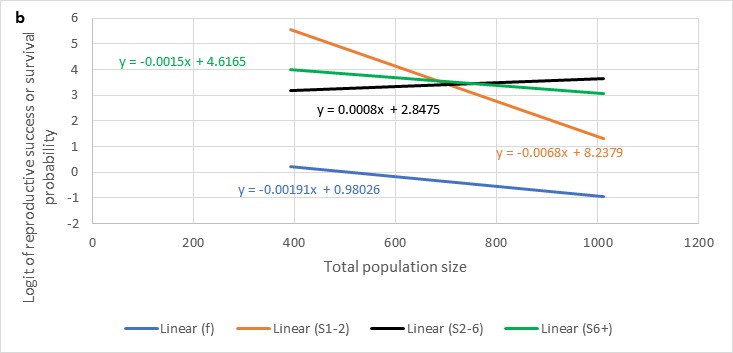


**Fig. A. Plots of density-dependent equations for survival (Sx) and reproductive success (f).** The top (a) has equations reported in standardized total population size units (Margalida et al. 2020) and in the bottom (b) they are converted to total population size units. Equations are in logit form for survival and reproduction (maximum number of fledglings per female is 1).


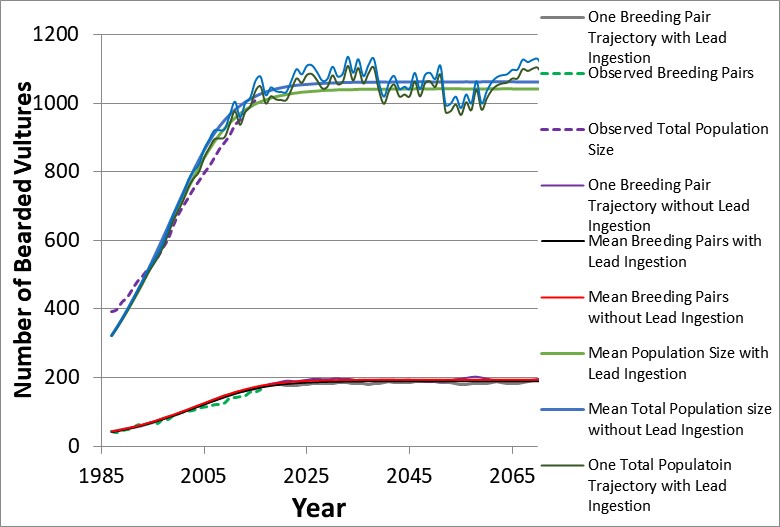


**Fig. B. Comparison of observed trend (dashed lines from 1987 to 2016) to modeled trend (solid lines from 1987 to 2070) of breeding pair (includes triplets) and total population size.**


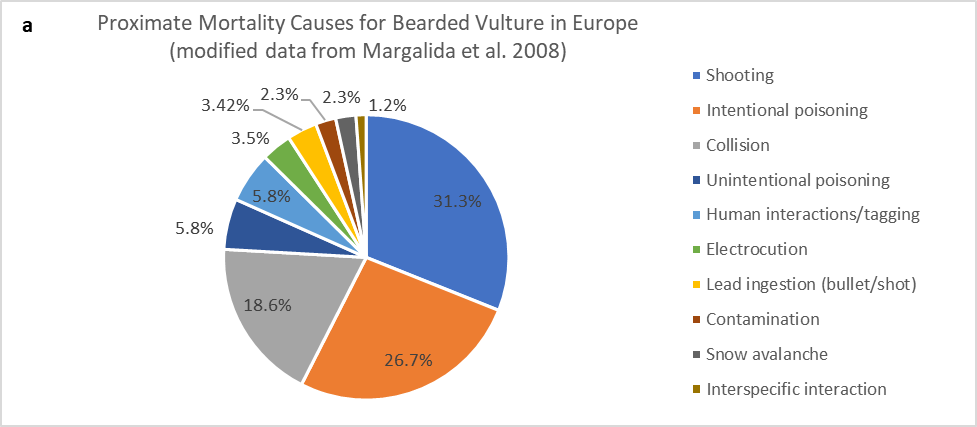


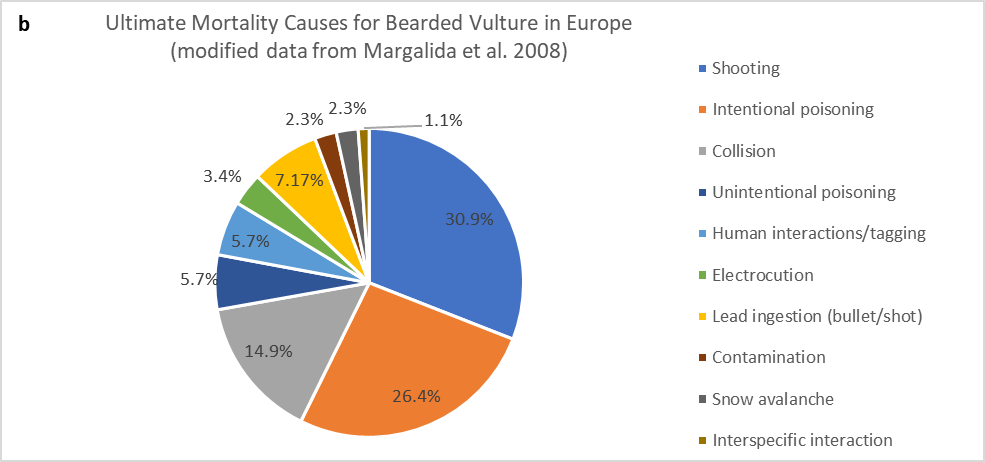


**Fig. C. The lower (a) and upper (b) bound of the percent of carcasses with potential lead poisoning from ammunition ingestion for the bearded vulture in Europe used in model.** The other causes are shown to indicate how adjusting the lead estimate for sublethal deaths in (b) must reduce the proportions of other causes (must sum to 100%); in this case we arbitrarily removed it from collision in (b), assuming lead-weakened birds were more susceptible to collisions (no information was given on ultimate cause). Data are from Margalida et al. (2008), but they did not separate lead poisoning from other unintentional sources of poisoning and indicated they included birds in the unintentional poisoned category even if died of another cause (thus their unintentional poisoning in [a] is an overestimate for proximate cause as it includes some ultimate cause deaths). We modified their unintentional poisoning (was 8%) in (a) and (b) by splitting out lead poisoning based on our analysis of percent with a proximate and ultimate cause by lead, respectively. The reduction to bearded vultures could be estimated for each cause, but this paper is focused on lead poisoning, which was ranked 7^th^ of the 10 proximal sources.

**References**

Margalida A, Jimenez J, Martinez JM, Sese JA, Garcia-Ferre D, Llamas A, et al. An assessment of population size and demographic drivers of the bearded vulture using integrated population models. Ecol Monogr. 2020; 90: e01414.

Meyer CB, Meyer JS, Francisco AB, Holder J, Verdonck F. Can ingestion of lead shot and poisons change population trends of three European birds: Grey partridge, common buzzard, and red kite? PLoS ONE. 2016;11. doi:10.1371/journal.pone.0147189

Sibly RM, Barker D, Denham NC, Hone J, Pagel M. On the regulation of populations of mammals, birds, fish and insects. Science. 2005; 309:607–610. PMID: 16040705
